# Resolving Oxygenation Pathways in Manganese-Catalyzed C(sp^3^)–H Functionalization via Radical and Cationic Intermediates

**DOI:** 10.1021/jacs.2c01466

**Published:** 2022-04-13

**Authors:** Marco Galeotti, Laia Vicens, Michela Salamone, Miquel Costas, Massimo Bietti

**Affiliations:** † Dipartimento di Scienze e Tecnologie Chimiche, Università“Tor Vergata”, Via della Ricerca Scientifica, 1, I-00133 Rome, Italy; ‡ QBIS Research Group, Institut de Química Computacional i Catàlisi (IQCC) and Departament de Química, Universitat de Girona, Campus Montilivi, Girona E-17071, Catalonia, Spain

## Abstract

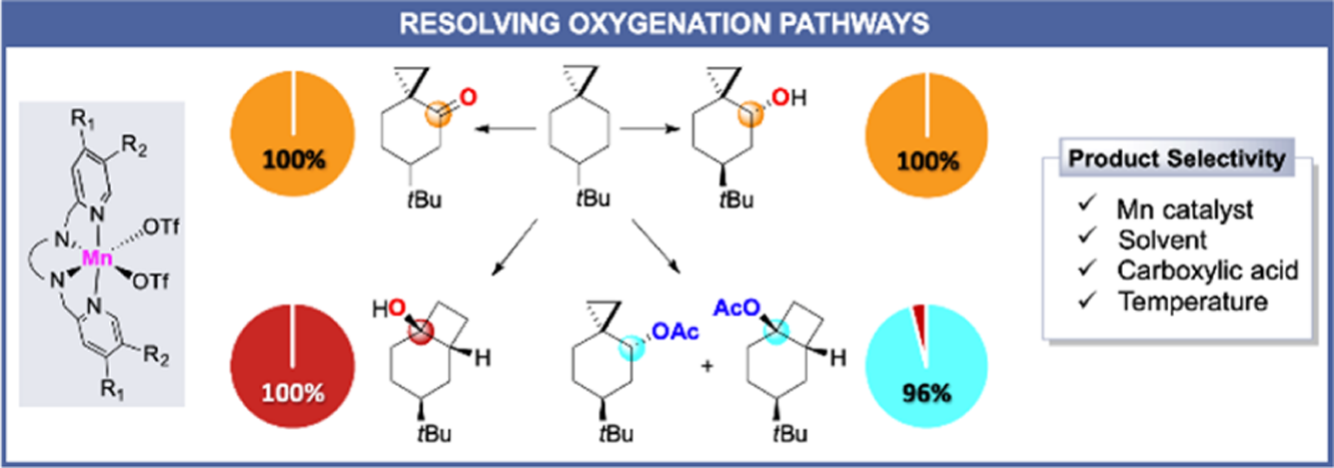

The C(sp^3^)–H bond oxygenation of the cyclopropane-containing mechanistic probes 6-*tert*-butylspiro[2.5]octane and spiro[2.5]octane with hydrogen peroxide catalyzed by manganese complexes bearing aminopyridine tetradentate ligands has been studied. Mixtures of unrearranged and rearranged oxygenation products (alcohols, ketones, and esters) are obtained, suggesting the involvement of cationic intermediates and the contribution of different pathways following the initial hydrogen atom transfer-based C–H bond cleavage step. Despite such a complex mechanistic scenario, a judicious choice of the catalyst structure and reaction conditions (solvent, temperature, and carboxylic acid) could be employed to resolve these oxygenation pathways, leading, with the former substrate, to conditions where a single unrearranged or rearranged product is obtained in good isolated yield. Taken together, the work demonstrates an unprecedented ability to precisely direct the chemoselectivity of the C–H oxidation reaction, discriminating among multiple pathways. In addition, these results conclusively demonstrate that stereospecific C(sp^3^)–H oxidation can take place via a cationic intermediate and that this path can become exclusive in governing product formation, expanding the available toolbox of aliphatic C–H bond oxygenations. The implications of these findings are discussed in the framework of the development of synthetically useful C–H functionalization procedures and the associated mechanistic features.

## Introduction

**Scheme 1:**
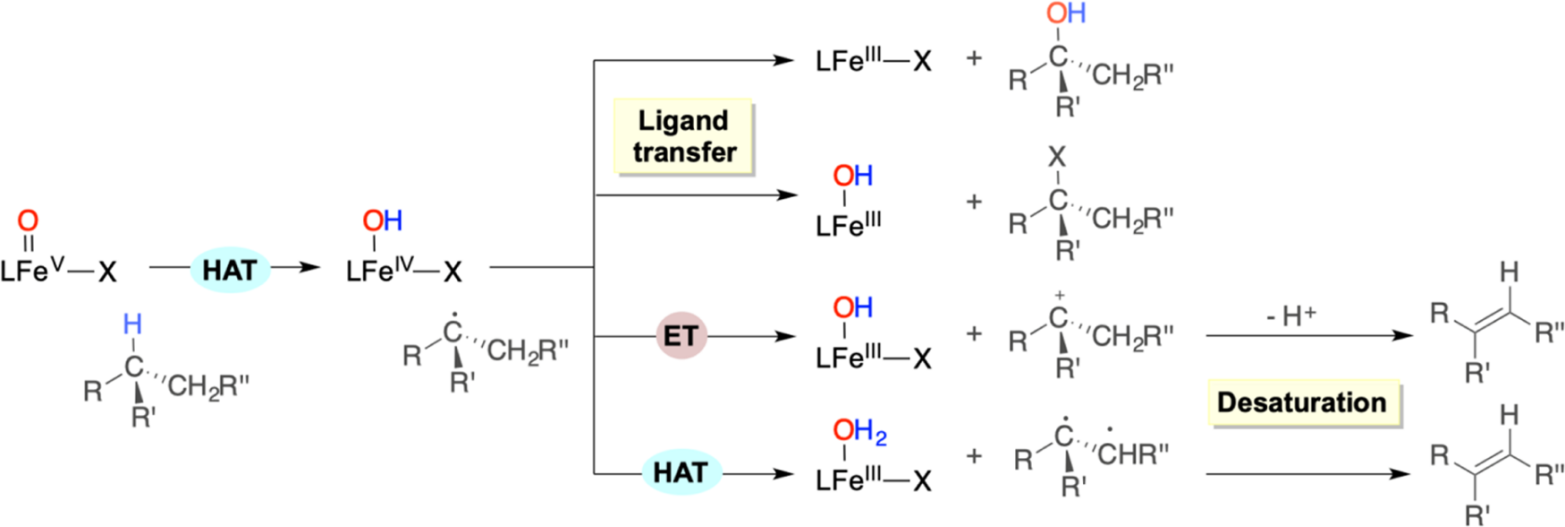
Mechanisms of Enzymatic C–H Oxidation by High-Valent Fe(V)=O Species

C(sp^3^)–H bond oxygenation is an important class of C–H functionalization reactions that is attracting increasing interest because of the ubiquity of oxidized aliphatic frameworks in molecules of biological and pharmaceutical interest and of the rich chemistry associated to C–O bond elaboration, amenable for broad product diversification.^1−4^ C(sp^3^)–H bond oxidation is performed by numerous iron-dependent enzymes that operate via high valent iron-oxo species.^5−8^ The structures of these enzymes and the associated reaction mechanisms have served as inspiration motifs for the development of C–H oxidation catalysts.^9−13^ Investigated for long, the current mechanistic consensus is that heme and non-heme monoiron-dependent oxygenases hydroxylate C–H bonds by a similar rebound mechanism (Scheme 1).^6,14−18^ The reaction is initiated by a hydrogen atom transfer (HAT) from a substrate C–H bond to generate a carbon radical that is then trapped by hydroxyl ligand transfer to form the hydroxylated product. In monoiron-dependent non-heme enzymes, the structural versatility of the metal coordination sphere enables alternative reactivity patterns;^19^ for example, the transfer of halide and pseudohalide ligands adjacent to the hydroxyl defines the reactivity of iron-dependent halogenases in the ligand transfer step.^20−22^ On the other hand, desaturation pathways initiated by HAT from C(sp^3^)–H bonds are seldom observed, for example, in the non-heme Fe^II^/α-ketoglutarate-dependent dioxygenase AsqJ,^23^ with two divergent mechanisms, which may account for this reactivity pattern, currently under debate. The first one entails an electron transfer (ET) from the carbon radical to the metal to form a carbocation that then evolves via proton loss to the desaturation product. Alternatively, a second HAT from the C–H bond adjacent to the carbon radical can also account for desaturation. In perspective, this complex mechanistic landscape represents an opportunity to diversify chemoselectivity in C(sp^3^)–H oxidation.

Iron and manganese complexes containing tetradentate aminopyridine ligands are powerful C–H oxidation catalysts that are able to promote selective aliphatic C–H bond hydroxylation. Mechanistic studies point toward a common mechanistic scenario for iron and manganese catalysts where C–H hydroxylation proceeds via an enzymatic-like HAT/rebound mechanism executed by high-valent metal-oxo species.^11−13,16^ Alternative reactions entailing the transfer of the ligand cis to oxo^24−27^ and desaturation^28^ have been observed only in selected cases where, however, these pathways are always accompanied by the canonical hydroxylation reaction, which typically dominates the reactivity. A general understanding of the factors that govern these divergent reactivities is lacking and evidence in favor of cationic paths is scarce. As a consequence, catalytic C–H oxidation reactions where product chemoselectivity can be reliably manipulated among multiple reaction paths in a predictable manner remain a standing challenge.

Among the substrates that are amenable for this purpose, cyclopropane-containing hydrocarbons are particularly appealing. The presence of the cyclopropyl group has been shown to activate adjacent sites toward HAT via hyperconjugative overlap between a cyclopropane C–C bonding orbital and the α-C–H σ* antibonding orbital,^29^ providing a powerful handle to implement site-selectivity in these reactions.^30,31^ In addition, because hyperconjugative effects also account for the stabilization of cyclopropylcarbinyl cations,^32^ these substrates can offer the opportunity to access cationic intermediates via sequential HAT–ET steps. However, because the intermediate α-cyclopropyl carbon radicals formed in the HAT step are known to undergo rapid rearrangement,^33^ access to unrearranged functionalized products or to products deriving from cationic intermediates is limited to the use of reagents that ensure very fast capture or one-electron oxidation of the radical intermediate, preventing competitive unimolecular radical pathways. Examples of reagents that are known to promote stereoretentive C(sp^3^)–H oxygenations are represented by metal-oxo species and dioxiranes.^4,16,34^

**Figure 1:**
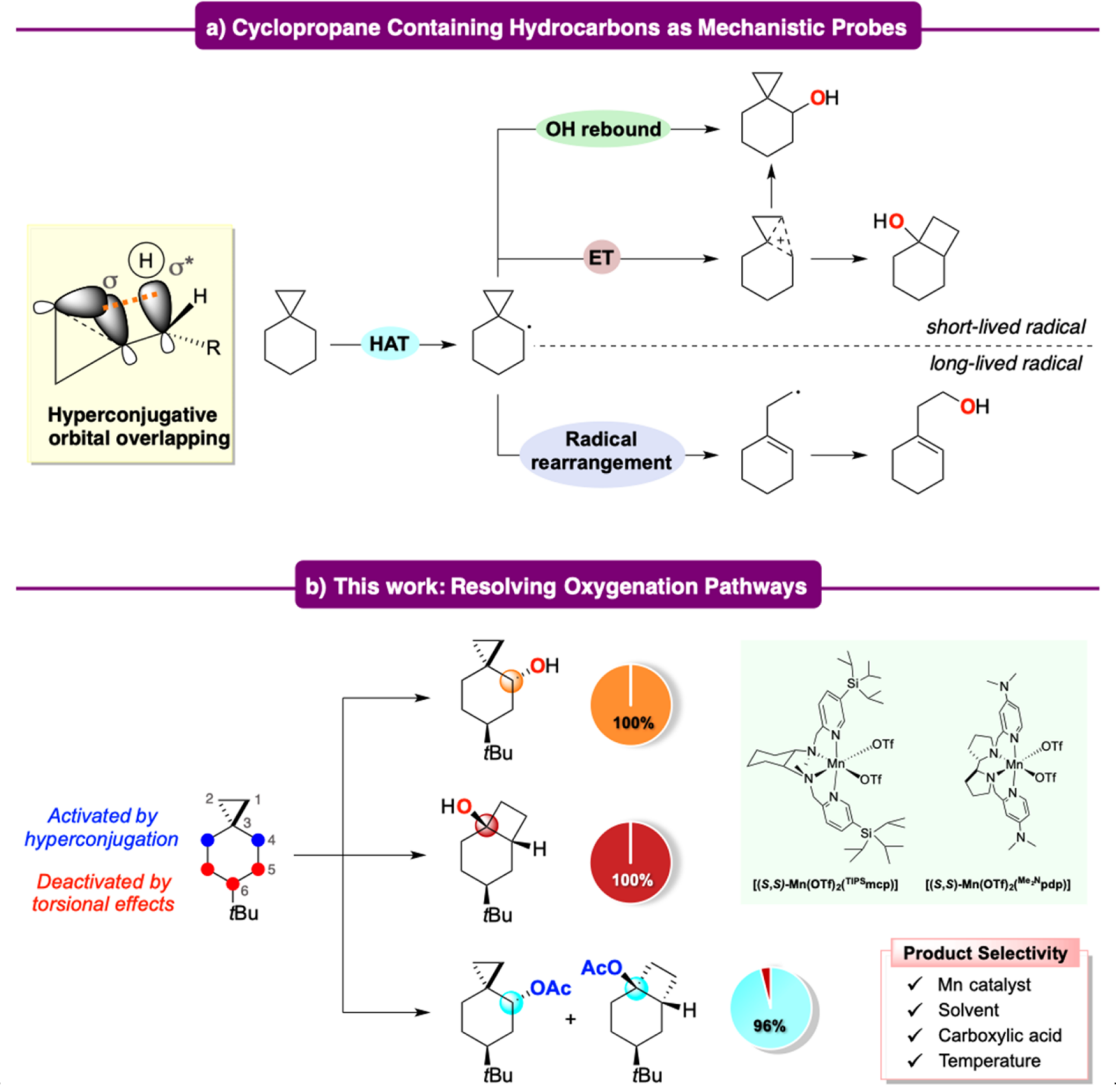
(a) Use of cyclopropane-containing hydrocarbons as mechanistic probes; (b) use of 6-*tert*-butylspiro[2.5]octane as a substrate to resolve the oxygenation pathways of Mn-catalyzed C–H oxygenation.

Because of their characteristic structural and bonding features,^29^ cyclopropane-containing substrates are customarily employed to probe the involvement of radical intermediates in a reaction^35−41^ to assess the concerted, radical, and/or cationic nature of enzymatic and biomimetic reaction mechanisms^6,42,43^ as well as to calibrate the rates of competing radical reactions (Figure 1a).^44^

With a substrate such as spiro[2.5]octane (Figure 1a), the corresponding α-cyclopropyl carbon radical undergoes cyclopropane ring-opening with *k*_r_ = 5 × 10^7^ s^–1^.^45^ In the framework of the oxygenation of this substrate promoted by metal-oxo species, dioxiranes, ozone, and cytochrome P450 enzymes,^30,45−47^ no evidence for the formation of products deriving from a radical rearrangement has been observed, in line with the relatively low value of *k*_r_ that prevents the competition of this pathway with the radical capture or radical recombination steps.

The product distributions observed in the reactions of this substrate and of bicyclo[4.1.0]heptane (norcarane) can provide moreover information on the involvement of cationic intermediates, revealing the occurrence of competitive ET steps.^45^ In the specific case of spiro[2.5]octane (Figure 1a), formation of bicyclo[4.2.0]octan-1-ol can provide conclusive evidence on the involvement of a cationic intermediate. To the best of our knowledge however, no evidence for the formation of bicyclo[4.2.0]octan-1-ol has been obtained in the oxygenations of spiro[2.5]octane discussed above.^30,45−47^

However, a typical drawback associated with the use of probes such as spiro[2.5]octane and norcarane is represented by the presence of several methylene sites on the cyclohexane ring that, although less activated than those adjacent to the cyclopropyl group, can lead nevertheless to the formation of isomeric oxygenation products. Along these lines, we reasoned that by introducing a *tert*-butyl group on the cyclohexane ring of spiro[2.5]octane as in 6-*tert*-butylspiro[2.5]octane,^48^ this group may impose torsional and steric deactivation at the tertiary and secondary C–H bonds at C-5 and C-6,^49^ directing HAT toward the C–H bonds at C-4 that benefit from hyperconjugative activation imparted by the cyclopropyl group (Figure 1b). The presence of this group would allow moreover to discriminate between the axial and equatorial C–H bonds at this site.

With these concepts in mind, we report herein a detailed mechanistic study on the C–H bond oxidation of 6-*tert*-butylspiro[2.5]octane with hydrogen peroxide catalyzed by manganese complexes. Unprecedented evidence for the formation of a cationic intermediate is provided, showing moreover that despite a complex mechanistic scenario, a careful choice of the catalyst and fine tuning of the reaction conditions (solvent, temperature, and carboxylic acid) have allowed the development of experimental conditions for a distinction between radical and cationic pathways, leading to a set of single product reactions where the unrearranged or rearranged products are obtained in high yield and outstanding selectivity.

## Results

**Scheme 2:**
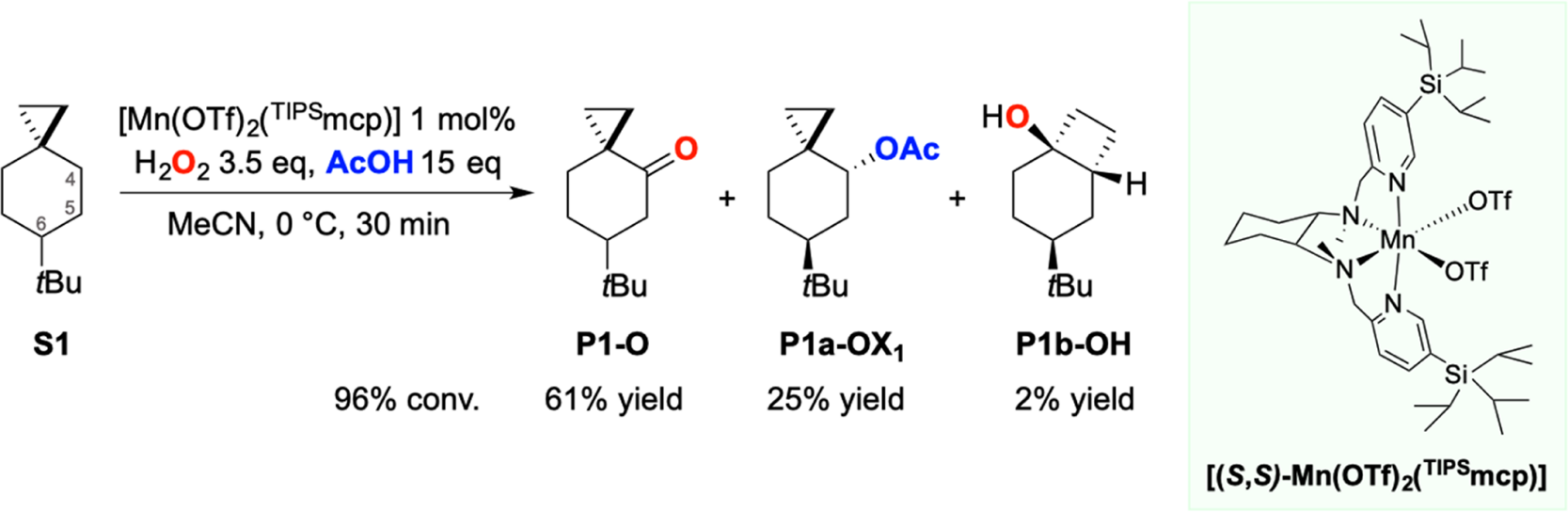
Oxidation of **S1** Reaction conditions: [Mn(OTf)_2_(^TIPS^mcp)] 1 mol %, H_2_O_2_ 3.5 equiv, AcOH 15 equiv, MeCN, 0 °C, 30 min. Catalyst enantiomers were used interchangeably.

The oxidation of 6-*tert*-butylspiro[2.5]octane (**S1**) was initially performed using 3.5 equiv of H_2_O_2_ delivered over 30 min using a syringe pump in the presence of 15 equiv of carboxylic acid and 1 mol % of catalyst at 0 °C in MeCN as the solvent (0.125 M substrate concentration). Under these conditions, reaction optimization identified [Mn(OTf)_2_(^TIPS^mcp)] as the best performing catalyst (see Table S3 in the Supporting Information). Previous studies have shown the ability of this catalyst to efficiently promote aliphatic C–H bond oxygenation at methylene sites.^50,51^ The oxidation of **S1** was thus performed under the optimized conditions in the presence of 15 equiv of acetic acid and 1 mol % [Mn(OTf)_2_(^TIPS^mcp)]. The formation of 6-*tert*-butylspiro[2.5]octan-4-one (**P1-O**) in 61% yield was observed, accompanied by *trans*-6-*tert*-butylspiro[2.5]octan-4-yl acetate (**P1a-OX**_**1**_) in 25% yield. Trace amounts (2%) of *cis*-4-(*tert*-butyl)-bicyclo[4.2.0]octan-1-ol (**P1b-OH**) were also observed (Scheme 2). No product deriving from C–H bond oxidation at C-5 and C-6 was observed.

**Scheme 3:**
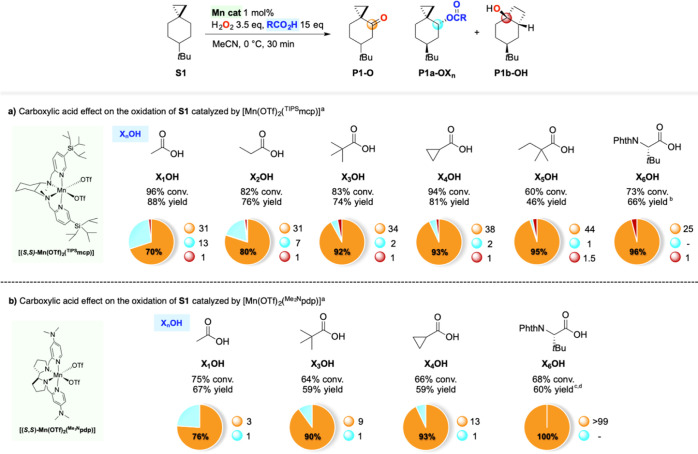
Oxidation of **S1** Using Different Carboxylic Acids Pie charts refer to product selectivities and adjacent small circles to normalized product ratios. Reaction conditions: (a) [Mn(OTf)_2_(^TIPS^mcp)] 1 mol %, H_2_O_2_ 3.5 equiv, RCO_2_H 15 equiv, MeCN, 0 °C, 30 min. (b) [Mn(OTf)_2_(^Me2N^pdp)] 1 mol %, H_2_O_2_ 2.5 equiv, RCO_2_H 15 equiv, MeCN, 0 °C, 30 min. ^a^Catalyst enantiomers were used interchangeably. ^b^[Mn(OTf)_2_(^TIPS^mcp)] 5 mol %, H_2_O_2_ 5 equiv, Phth-Tle-OH 1.0 equiv. Addition of [Mn(OTf)_2_(^TIPS^mcp)] 5 mol % and Phth-Tle-OH 1.0 equiv after 10 and 20 min. ^c^Phth-Tle-OH 1.0 equiv, H_2_O_2_ 3.5 equiv. ^d^Isolated yield.

By changing the carboxylic acid additive, **P1-O** was always the major product, accompanied by varying amounts of the corresponding ester **P1a-OX**_***n***_, and by the rearranged alcohol product **P1b-OH**, which was formed in all cases in ≤2.5% yield (Scheme 3a). Very interestingly, the **P1-O**/**P1a-OX**_***n***_ ratio increases with increasing carboxylic acid steric bulk, changing from 2.4 to 44 on going from acetic acid to 2,2-dimethylbutanoic acid, leading, in the latter case, to the formation of product **P1-O** in 95% selectivity over **P1a-OX**_**5**_ and **P1b-OH**. When the same reaction was carried out in the presence of phtalimido-protected *tert*-leucine (Phth-Tle-OH) as the acid (3.0 equiv), the formation of **P1-O** in 63% yield and 96% selectivity over **P1b-OH** was observed, without detection among the reaction products of the corresponding ester **P1a-OX**_**6**_.

By changing the catalyst to the more electron-rich [Mn(OTf)_2_(^Me2N^pdp)], oxidation of **S1** in the presence of acetic acid, pivalic acid, or cyclopropanecarboxylic acid afforded **P1-O** as the major product (51–55% yield), accompanied in all cases by the corresponding ester **P1a-OX**_***n***_, in a **P1-O**/**P1a-OX**_***n***_ ratio of 3.2, 8.8, and 14, respectively (Scheme 3b), in line with the trend observed with the [Mn(OTf)_2_(^TIPS^mcp)] catalyst. In all three cases however, no trace of the rearranged alcohol **P1b-OH** was detected among the reaction products. Most interestingly, when Phth-Tle-OH was employed as the acid co-catalyst, the oxidation of **S1** with H_2_O_2_ catalyzed by [Mn(OTf)_2_(^Me2N^pdp)] led to the exclusive formation of **P1-O** in 60% isolated yield.

**Scheme 4:**
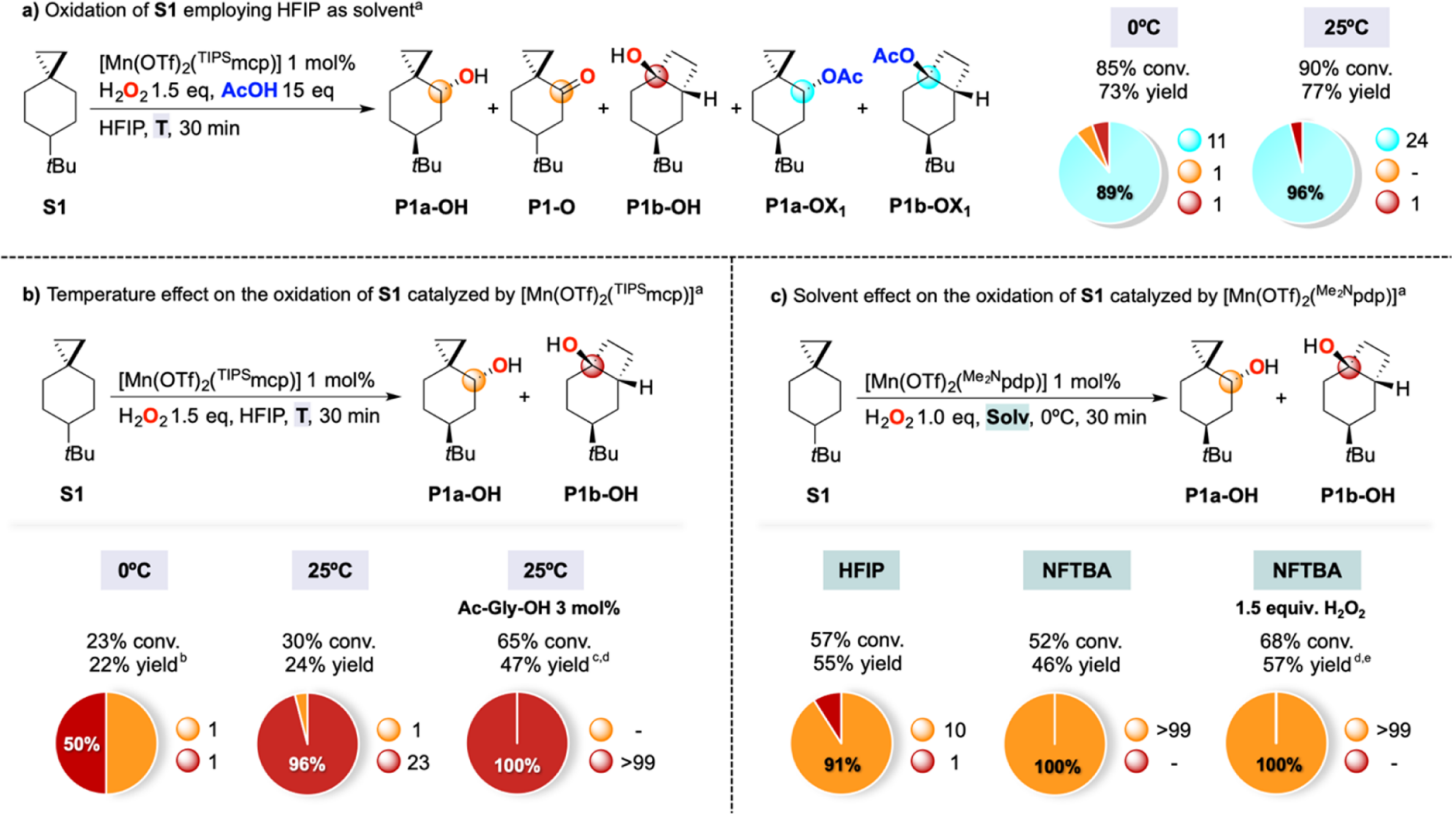
Oxidation of **S1** at Different Temperatures and in Different Solvents Pie charts refer to product selectivities and adjacent small circles to normalized product ratios. Reaction conditions: (a) [Mn(OTf)_2_(^TIPS^mcp)] 1 mol %, H_2_O_2_ 1.5 equiv, AcOH 15 equiv, HFIP, 0 or 25 °C, 30 min. (b) [Mn(OTf)_2_(^TIPS^mcp)] 1 mol %, H_2_O_2_ 1.5 equiv, HFIP, 0 or 25 °C, 30 min. (c) [Mn(OTf)_2_(^Me2N^pdp)] 1 mol %, H_2_O_2_ 1.0 equiv, HFIP or NFTBA, 0 °C, 30 min. ^a^Catalyst enantiomers were used interchangeably. ^b^8% yield of **P1a-OH** and 3% yield of **P1-O**. ^c^Ac-Gly-OH 3 mol %. ^d^Isolated yield. ^e^1.5 equiv of H_2_O_2_.

1,1,1,3,3,3-Hexafluoro-2-propanol (HFIP) was then chosen as the solvent in order to obtain information on the role of medium effects on the reaction outcome.^52^ When the oxidation of **S1** was performed in this solvent (0.125 M substrate concentration) at 0 °C, using 1.5 equiv of H_2_O_2_ in the presence of 15 equiv of acetic acid and 1 mol % of [Mn(OTf)_2_(^TIPS^mcp)], a predominant formation of the unrearranged and rearranged acetate ester products **P1a-OX**_**1**_ and **P1b-OX**_**1**_ in 44 and 21% yields, respectively, was observed, accompanied by smaller amounts of **P1a-OH**, **P1-O**, and **P1b-OH** (overall 7.5% yield, Scheme 4a). When the same reaction was performed at 25 °C, the acetate esters were formed in 74% combined yield (**P1a-OX**_**1**_/**P1b-OX**_**1**_ = 1.2) and 96% selectivity over the rearranged alcohol **P1b-OH**, while **P1a-OH** and **P1-O** were not detected among the reaction products. A similar outcome was observed when other carboxylic acids were used, where however the **P1a-OX**_***n***_/**P1b-OX**_***n***_ ratio was observed to increase with increasing carboxylic acid steric bulk, approaching a value of 3.3 when employing pivalic acid (see the Supporting Information for full details).

With these results in hand, we envisioned the possibility of carrying out the oxidation of **S1** in the absence of carboxylic acid, taking advantage of the ability of fluorinated alcohol solvents to assist in hydrogen peroxide activation (Scheme 4b).^53,54^ When the oxidation of **S1** was performed in HFIP at 0 °C, using 1.5 equiv of H_2_O_2_ and 1 mol % of [Mn(OTf)_2_(^TIPS^mcp)], the formation of the unrearranged alcohol and ketone products **P1a-OH** and **P1-O** in 11% total yield was observed, accompanied by 11% of the rearranged alcohol **P1b-OH**. When the same reaction was performed at 25 °C, **P1b-OH** was formed in excellent selectivity (>96%) over **P1a-OH** albeit in low yield (23%). An increase in substrate conversion was observed when HFIP was replaced by nonafluoro-*tert*-butyl alcohol (NFTBA), without improvements in terms of selectivity (see the Supporting Information for full details). Under the same conditions (HFIP, *T* = 25 °C), the addition of 3 mol % of *N*-acetyl glycine (Ac-Gly-OH) was shown to increase the efficiency of the oxidation reaction, leading to the exclusive formation of **P1b-OH** in 47% isolated yield.

By changing the catalyst to [Mn(OTf)_2_(^Me2N^pdp)],^55^ the oxidation of **S1** in HFIP at 0 °C led to the formation of **P1a-OH** in 50% yield and 91% selectivity over **P1b-OH**, in significantly higher combined yield (55%) and selectivity (Scheme 4c) as compared to those observed when employing the [Mn(OTf)_2_(^TIPS^mcp)] catalyst (22% combined yield, 50% selectivity, Scheme 4b). The exclusive formation of **P1a-OH** in 46% yield (100% selectivity) was observed when HFIP was replaced by NFTBA, approaching 57% isolated yield without affecting selectivity when increasing H_2_O_2_ loading from 1.0 to 1.5 equiv. Identical results were obtained when the latter reaction was carried out on a larger scale (see Supporting Information, Table S12).

**Scheme 5:**
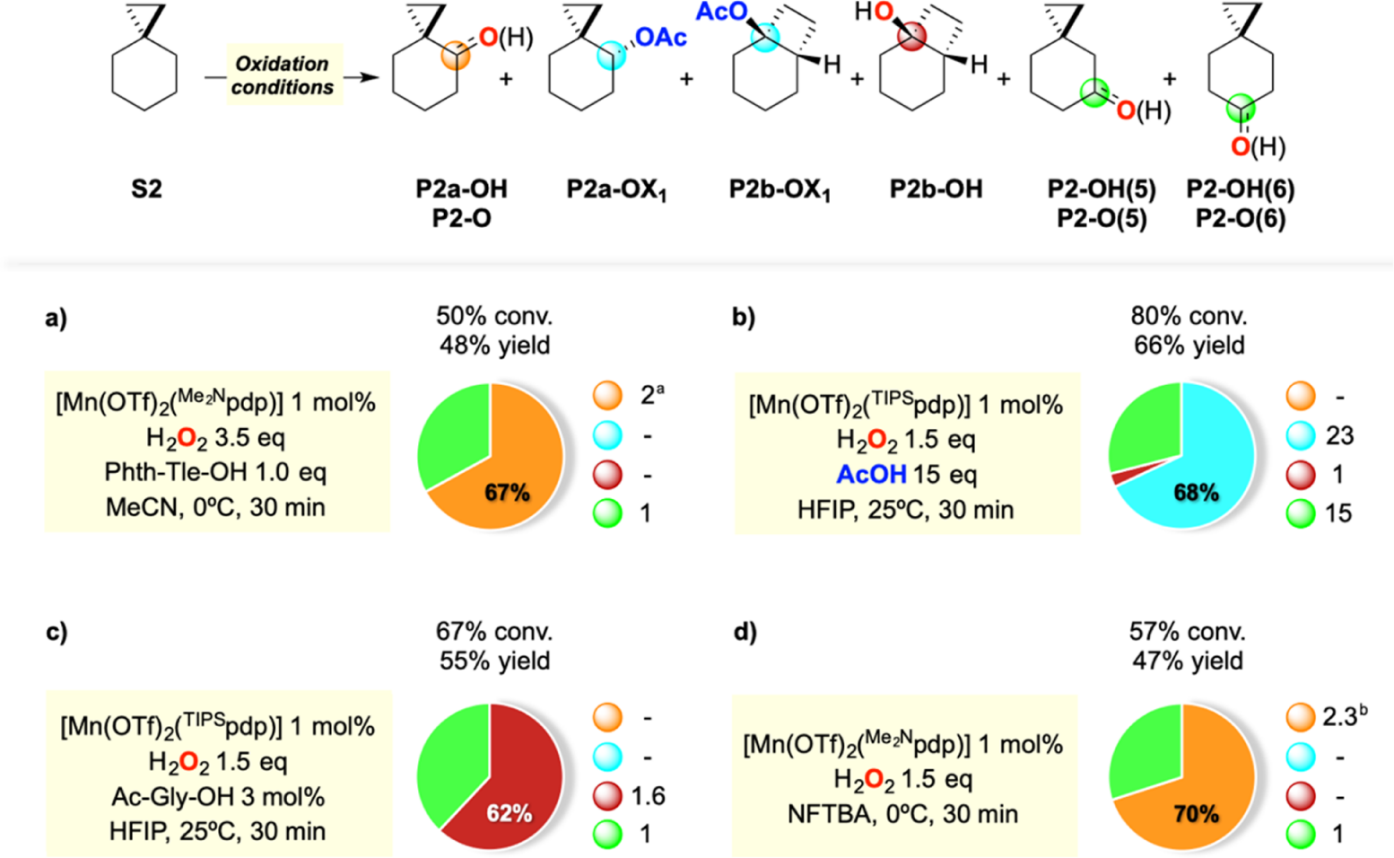
Oxidation of **S2** Pie charts refer to product selectivities and adjacent small circles to normalized product ratios. Reaction conditions: (a) [Mn(OTf)_2_(^Me2N^pdp)] 1 mol %, H_2_O_2_ 3.5 equiv, Phth-Tle-OH 1.0 equiv, MeCN, 0 °C, 30 min. (b) [Mn(OTf)_2_(^TIPS^pdp)] 1 mol %, H_2_O_2_ 1.5 equiv, AcOH 15 equiv, HFIP, 25 °C, 30 min. (c) [Mn(OTf)_2_(^TIPS^pdp)] 1 mol %, H_2_O_2_ 1.5 equiv, Ac-Gly-OH 3 mol %, HFIP, 25 °C, 30 min. (d) [Mn(OTf)_2_(^Me2N^pdp)] 1 mol %, H_2_O_2_ 1.5 equiv, NFTBA, 0 °C, 30 min. ^a^Formation of **P2-O** as the exclusive oxidation product at C-4. ^b^Formation of **P2a-OH** as the exclusive oxidation product at C-4.

In order to probe the applicability of these concepts, the optimized conditions described above were then extended to the oxidation of the unsubstituted spiro[2.5]octane (**S2**) and the results thus obtained are displayed in Scheme 5.

Under all conditions, the reaction outcome closely parallels those obtained in the oxidation of **S1**, with the only difference being represented by the formation of sizable amounts (between 14 and 21% yields, 29–38% product selectivity) of oxidation products deriving from hydroxylation or ketonization at C-5 and C-6. As compared to the reactions of **S1**, better results were obtained when employing the [Mn(OTf)_2_(^TIPS^pdp)] catalyst in place of [Mn(OTf)_2_(^TIPS^mcp)] (see the Supporting Information). Along this line, the oxidation of **S2** with 3.5 equiv of H_2_O_2_ in the presence of 1.0 equiv of Phth-Tle-OH and 1 mol % of [Mn(OTf)_2_(^Me2N^pdp)] at 0 °C in MeCN led to the formation of spiro[2.5]octan-4-one (**P2-O**) in 30% yield as the exclusive oxidation product at C-4, accompanied by spiro[2.5]octan-5-one (**P2-O(5)**) and spiro[2.5]octan-6-one (**P2-O(6)**) in 7 and 8% yields, respectively (Scheme 5a). When the oxidation of **S2** was carried out in HFIP at 25 °C, using 1.5 equiv of H_2_O_2_ in the presence of 15 equiv of acetic acid and 1 mol % of [Mn(OTf)_2_(^TIPS^pdp)], the predominant formation of the unrearranged and rearranged acetate ester products **P2a-OX**_**1**_ and **P2b-OX**_**1**_ in 26 and 19% yields, respectively, was observed, in 96% selectivity for C-4 oxidation over bicyclo[4.2.0]octan-1-ol (**P2b-OH**) (formed in 2% yield), accompanied by spiro[2.5]octan-5-ol (**P2a-OH(5)**) and spiro[2.5]octan-6-ol (**P2a-OH(6)**) in 11 and 8% yields, respectively (Scheme 5b). By replacing acetic acid with Ac-Gly-OH (3 mol %), the formation of **P2b-OH** in 34% yield as the exclusive oxidation product at C-4 was observed, accompanied by **P2a-OH(5)** and **P2a-OH(6)** in 12 and 9% yields, respectively (Scheme 5c). Finally, when the oxidation of **S2** was carried out in NFTBA at 0 °C, using 1.5 equiv of H_2_O_2_ and 1 mol % of [Mn(OTf)_2_(^Me2N^pdp)], in the absence of a carboxylic acid additive, the formation of **P2a-OH** in 33% yield as the exclusive oxidation product at C-4 was observed, accompanied by **P2a-OH(5)** and **P2a-OH(6)** in 8 and 6% yields, respectively (Scheme 5d).

## Discussion

The results presented above clearly indicate that in the oxidation of **S1** under the different conditions employed, all the observed products result from site-selective C–H bond functionalization at C-4. No product deriving from functionalization at other sites was observed, confirming that the synergistic cooperation of deactivating torsional and steric effects and hyperconjugative activation exerted by the *tert*-butyl and cyclopropyl groups, respectively, directs the functionalization toward C-4. As a matter of comparison, the corresponding reactions of the unsubstituted substrate **S2** led in all cases to the formation of significant amounts (up to 38%) of oxidation products deriving from oxygenation at C-5 and C-6 (Scheme 5).

**Figure 2:**
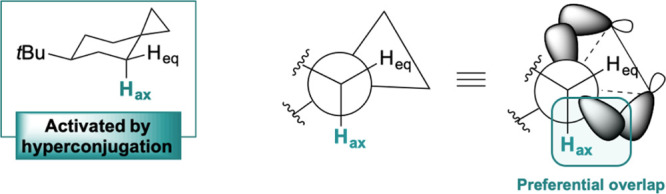
Origin of the observed diastereoselectivity in the formation of the unrearranged products in the oxidation of **S1**.

Analysis of the unrearranged products formed in the oxidation of **S1**, deriving from C-4 hydroxylation (in HFIP and NFTBA) and esterification (in MeCN and HFIP), shows in all cases the exclusive formation of the diastereoisomer where the oxygenated group is in a trans configuration to the *tert*-butyl group. C–H bond hydroxylations promoted by high-valent metal-oxo species are stereoretentive and have been proposed to occur through a mechanism that proceeds via initial HAT from a substrate C–H bond to give a metal-hydroxo species and a carbon radical that undergo very fast OH rebound.^16,56^ Along this line, the outstanding stereoselectivity observed in the formation of **P1a-OH** (and of **P1a-OX**_***n***_, see below) can be rationalized by taking into account the selective hyperconjugative activation of the axial C–H bond at C-4 provided by the spiro cyclopropyl group. In **S1**, the equatorial C–H bond at C-4 bisects the cyclopropane ring and accordingly cannot benefit from hyperconjugative overlap with the C-1–C-2 σ bonding orbitals, which is only possible for the axial C–H bond (Figure 2).

An analogous explanation can be put forward to account for the stereoselectivity observed in the C–H bond oxidation reaction promoted by TFDO, employed in an intermediate step of the total synthesis of (+)-phorbol.^31^

**Scheme 6:**
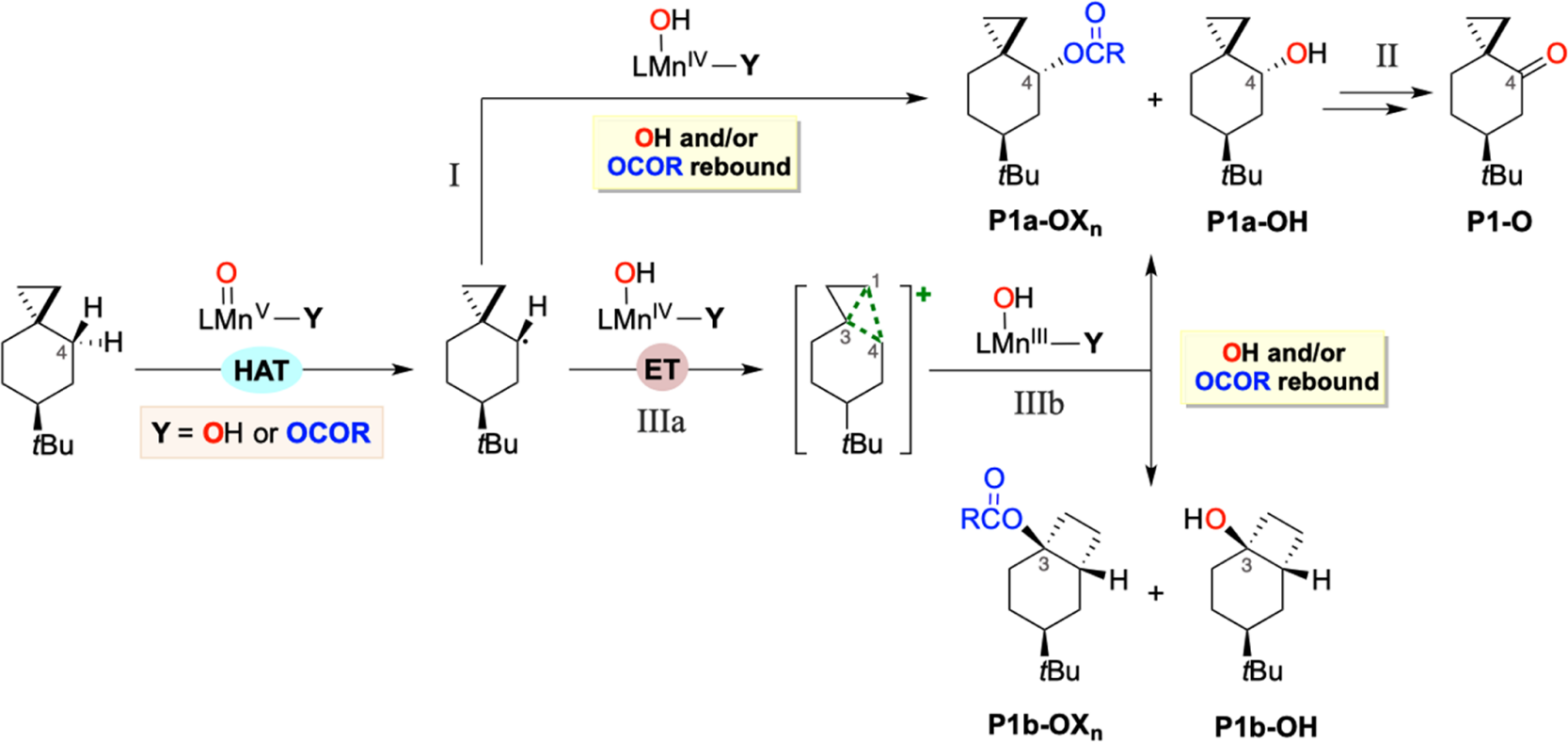
Proposed Mechanism for the Oxidation of **S1**

For what concerns the formation of the unrearranged esterification products **P1a-OX**_***n***_, mechanistic information was provided by means of control experiments carried out on *trans*-6-*tert*-butylspiro[2.5]octan-4-ol (**P1a-OH**), isolated from the oxidation reaction of **S1** in NFTBA described in Scheme 4c. When **P1a-OH** was added to a MeCN solution containing 3.5 equiv of H_2_O_2_ (delivered over 30 min using a syringe pump) and 15 equiv of acetic acid at 0 °C in the presence and absence of [Mn(OTf)_2_(^TIPS^mcp)], exclusive formation of **P1-O** deriving from the oxidation of the alcohol or complete recovery of **P1a-OH** was observed, respectively, with no formation of the acetate ester **P1a-OX**_**1**_ (see the Supporting Information for full details). These observations rule out the possibility that **P1a-OX**_**1**_ arises from esterification in the reaction medium of the first formed alcohol, suggesting that this product (and more generally **P1a-OX**_***n***_) derives from the intermediate carbon radical formed after the initial HAT step via stereoretentive acetate rebound (carboxylate rebound) that occurs in competition with hydroxyl rebound (Scheme 6, path **I**, Y = OCOR).

**P1a-OH** is then rapidly overoxidized to **P1-O** (Scheme 6, path **II**), whereas with **P1a-OX**_***n***_, the presence of the electron-withdrawing ester group electronically deactivates the α-C–H bond toward HAT, preventing overoxidation.

Support to the hypothesis of a carboxylate rebound is also provided by the observation that the (hydroxylation + ketonization)/esterification product ratio is not influenced by carboxylic acid loading (see the Supporting Information for full details), in line with product formation arising from a common manganese-hydroxo carboxylato intermediate (Scheme 6, path **I**, Y = OCOR). Within this mechanistic picture, the steric hindrance of the carboxylic acid additive plays a crucial role in determining the contribution of the carboxylate rebound pathway, with the relative importance of this pathway, quantified by the **P1-O**/**P1a-OX**_***n***_ ratio, that decreases with increasing steric bulk of the acid, being completely suppressed when employing the very bulky Phth-Tle-OH (Scheme 3a). An analogous competition has been recently proposed by Bryliakov and co-workers in the benzylic C–H bond oxygenation of cumene with H_2_O_2_ catalyzed by manganese complexes.^26^ In this study, the hydroxylation/esterification product ratio was observed to be unaffected by the carboxylic acid loading, increasing with increasing steric bulk of the carboxylic acid additive, supporting the involvement of competitive hydroxyl and carboxylate rebound pathways in these reactions.

The formation of the rearranged alcohol product *cis*-4-(*tert*-butyl)-bicyclo[4.2.0]octan-1-ol (**P1b-OH**) in small amounts (≤2.5% yield, Scheme 3a) in the oxidation of **S1** in MeCN catalyzed by [Mn(OTf)_2_(^TIPS^mcp)] supports the hypothesis of the formation of a cationic intermediate via a background ET reaction from the radical intermediate formed following HAT to the manganese-hydroxo species (Scheme 6, path **IIIa**). The observation that the formation of this product is suppressed when employing the more electron-rich [Mn(OTf)_2_(^Me2N^pdp)] catalyst in place of [Mn(OTf)_2_(^TIPS^mcp)] is in line with this hypothesis.

By changing the solvent from MeCN to HFIP, and employing [Mn(OTf)_2_(^TIPS^mcp)] as the catalyst and acetic acid as the co-catalyst, an increase in the relative amount of **P1a-OH**, **P1a-OX**_**1**_, and **P1b-OH** over **P1-O** was observed, accompanied by the formation of the rearranged ester product *cis*-4-(*tert*-butyl)-bicyclo[4.2.0]octan-1-yl acetate (**P1b-OX**_**1**_), with the esterification products being formed in 89% selectivity over the hydroxylation and ketonization ones (Scheme 4a). The increase in the relative amount of **P1a-OH** over **P1-O** can be explained on the basis of the well-established ability of fluorinated alcohol solvents to invert the polarity of hydroxyl groups by hydrogen bonding, electronically deactivating the adjacent C–H bonds toward HAT, thus preventing overoxidation.^57−59^ These solvents have been shown, moreover, to increase the oxidizing ability of ET reagents, stabilizing at the same time the charged intermediates.^60,61^ Along this line, the significant increase in the relative amount of rearranged over unrearranged oxygenation products observed on going from MeCN to HFIP can be accounted for on the basis of an increased contribution of the ET pathway (Scheme 6, path **IIIa**). In the cationic intermediate thus formed, the positive charge is significantly delocalized into the adjacent spiro cyclopropane ring,^32,62^ and hydroxide or acetate transfer from the reduced manganese-hydroxo carboxylato species can occur to the C-3 spiro carbon, leading to the formation of the rearranged products **P1b-OH** and **P1b-OX**_**1**_ and to the C-4 carbon, delivering **P1a-OH** and **P1a-OX**_**1**_ (Scheme 6, path **IIIb**, Y = OCOR). An increase in the ratio between rearranged and unrearranged products and between acetoxylation and hydroxylation (ketonization) products was observed by increasing the temperature from 0 to 25 °C, with the formation of the two esters in 96% selectivity over the rearranged alcohol **P1b-OH** (Scheme 3a), suggesting a preferential activation of the ET pathway under these experimental conditions.

**Scheme 7:**
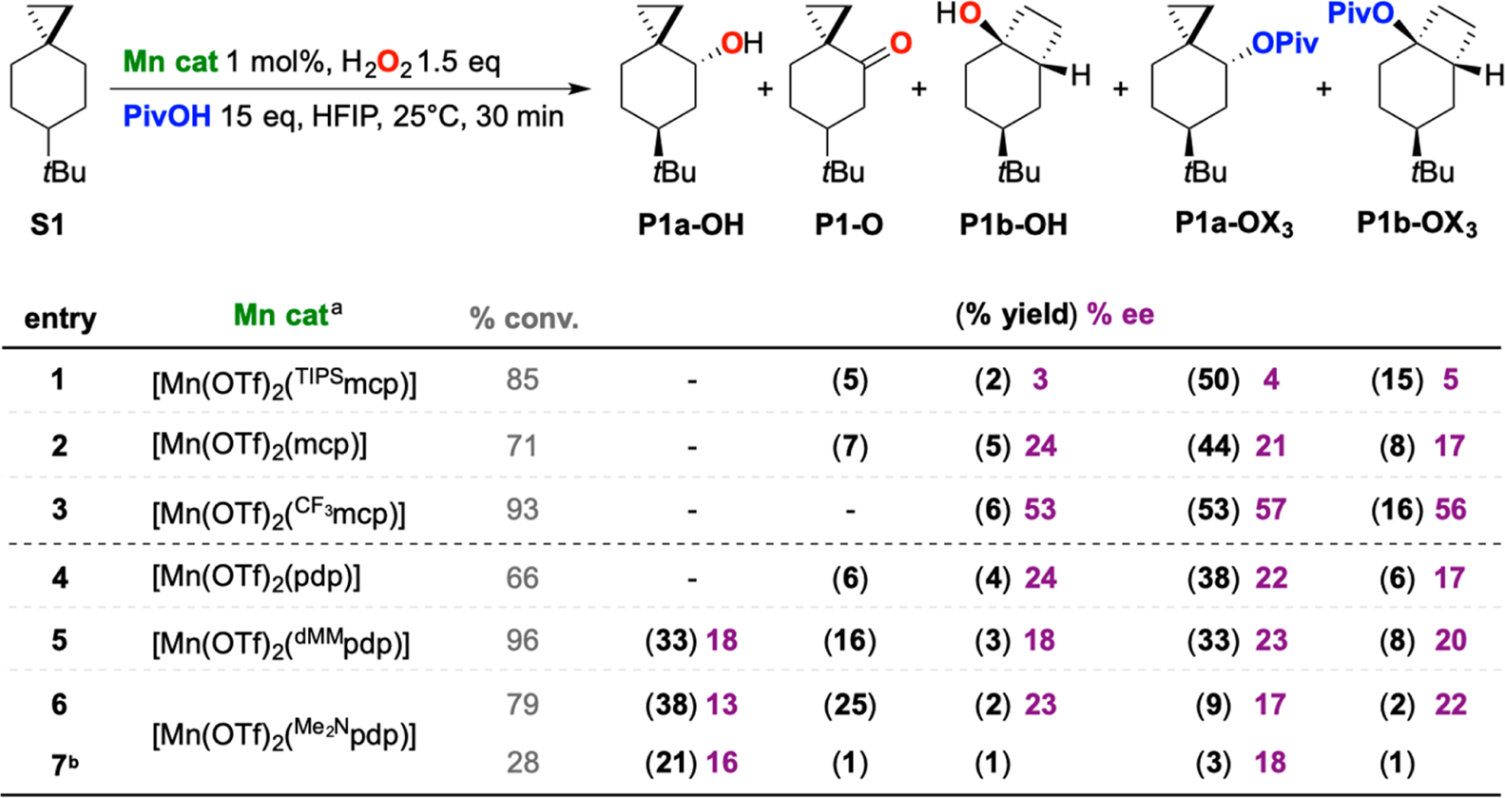
Oxidation of **S1** by Different Mn Catalysts Reaction conditions: Mn cat 1 mol %, H_2_O_2_ 1.5 equiv, PivOH 15 equiv, HFIP, 25 °C, 30 min. ^a^Catalyst structures are displayed in the Supporting Information (Figure S1). ^b^H_2_O_2_ 0.5 equiv.

Support to this mechanistic picture is also provided by the product enantioselectivities observed in the oxidation of **S1** with H_2_O_2_, catalyzed by different Mn complexes, carried out at 25 °C in HFIP in the presence of pivalic acid (Scheme 7). Although product distributions, yields, and enantiomeric excesses (ee’s) are influenced by the catalyst structure, in all the examples shown, very similar ee’s were measured for the unrearranged and rearranged alcohol and pivalate ester products (**P1a-OH**, **P1a-OX**_3_, **P1b-OH**, and **P1b-OX**_3_). This observation is consistent with the hypothesis that all four products arise from C–H bond cleavage by a common species,^63,64^ that is, the Mn^V^(O) (OCO*t*Bu) displayed in Scheme 6, which performs the initial HAT reaction.

In detail, very similar ee’s were observed for products **P1b-OH**, **P1a-OX**_**3**_, and **P1b-OX**_**3**_ when employing mcp-based catalysts and the [Mn(OTf)_2_(pdp)] catalysts (entries 1–4), with ee’s that significantly increased on going from [Mn(OTf)_2_(^TIPS^mcp)] to the [Mn(OTf)_2_(mcp)], [Mn(OTf)_2_(pdp)] and [Mn(OTf)_2_(^CF3^mcp)] catalysts, approaching 53–57% ee’s with the last one. In all four examples however, no formation of product **P1a-OH** was observed. The four products were instead observed in the reactions catalyzed by [Mn(OTf)_2_(^dMM^pdp)] and [Mn(OTf)_2_(^Me2N^pdp)] (entries 5 and 6). In the former case, 18–23% ee’s were measured for the four products, while in the latter one, a lower ee (13%) was measured for **P1a-OH** compared to the other three products (for which ee’s = 17–23%). This behavior can be reasonably accounted for on the basis of a kinetic resolution associated with the oxidation of **P1a-OH** to **P1-O**, in keeping with recent observations by Bryliakov and co-workers in benzylic C–H bond hydroxylations with H_2_O_2_ catalyzed by structurally related Mn(pdp) complexes.^65^ Accordingly, by decreasing H_2_O_2_ loading from 1.5 to 0.5 equiv (entry 7), trace amounts of the overoxidized product **P1-O** were observed, and very similar ee’s were then measured for **P1a-OH** and **P1a-OX**_**3**_. Full details of these experiments are provided in the Supporting Information.

**Scheme 8:**
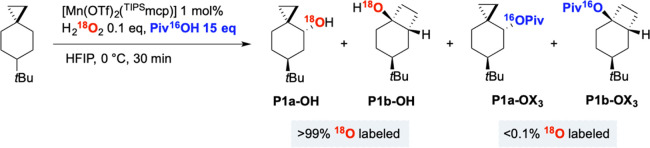
Oxidation of **S1** in the Presence of H_2_^18^O_2_ (80% Enriched in ^18^O) and Piv^16^OH Reaction conditions: [Mn(OTf)_2_(^TIPS^mcp)] 1 mol %, H_2_^18^O_2_ 0.1 equiv, Piv^16^OH 15 equiv, HFIP, 0 °C, 30 min. The labeling experiment was analyzed using GC–MS analysis via chemical ionization with NH_3_/NH_4_. The reported ^18^O incorporations were obtained after correction for the isotopic purity of the labeled reactant.

Information on the competition between hydroxide and carboxylate rebound pathways was also obtained by carrying out the oxidation of **S1** with labeled H_2_^18^O_2_ in the presence of unlabeled pivalic acid Piv^16^OH in HFIP, employing [Mn(OTf)_2_(^TIPS^mcp)] as the catalyst (Scheme 8). The ^18^O label was quantitatively retained in the products arising from hydroxylation (**P1a-OH** and **P1b-OH**) in agreement with the mechanism of C–H bond oxidation with H_2_O_2_ catalyzed by metal-oxo species, whereas the pivalate products (**P1a-OX**_**4**_ and **P1b-OX**_**4**_) only contained ^16^O. These results indicate that hydroxyl and carboxylate rebound can accompany the formation of both the unrearranged and rearranged products (Scheme 6, paths **I** and **IIIa–IIIb**, Y = OCOR) ruling out once again the hypothesis of a contribution to ester formation derived from the first formed alcohol products **P1a-OH** and **P1b-OH**.

On the basis of the effect of the temperature described above and leveraging on the ability of fluorinated alcohol solvents to assist in hydrogen peroxide activation,^53,54^ we reasoned that ester formation could be completely suppressed by carrying out the reaction in the absence of carboxylic acid. Along this line, by performing the oxidation of **S1** in HFIP at 25 °C employing [Mn(OTf)_2_(^TIPS^mcp)] as the catalyst, rearranged alcohol **P1b-OH** was formed in 96% selectivity over **P1a-OH** albeit in overall 24% yield (Scheme 4b). Interestingly, by carrying out the reaction in the presence of a catalytic amount (3 mol %) of Ac-Gly-OH, exclusive formation of **P1b-OH** in 47% isolated yield was observed. This result deserves special attention because, to the best of our knowledge, it represents an unprecedented example where straightforward access to a relevant bicyclo[4.2.0]octan-1-ol structure under mild reaction conditions is provided by means of HAT-initiated aliphatic C–H bond functionalization mediated by a cationic intermediate.

The product selectivity could be drastically changed by using the [Mn(OTf)_2_(^Me2N^pdp)] catalyst and NFTBA as the solvent. Under these conditions, the oxidation of **S1** with H_2_O_2_ (1.5 equiv) at 0 °C delivered **P1a-OH** as a single product in 57% isolated yield (Scheme 4c). Although the different outcomes observed with the [Mn(OTf)_2_(^Me2N^pdp)] and [Mn(OTf)_2_(^TIPS^mcp)] catalysts can be reasonably ascribed to the different oxidizing abilities of the intermediate manganese-hydroxo species, the possible formation of unrearranged products **P1a-OH** (and **P1-O**) and **P1a-OX**_***n***_ via both the HAT-rebound and HAT-ET-rebound pathways (Scheme 6, path **I** and **IIIa–IIIb**, respectively) does not allow a clear cut distinction between the relative contribution of these alternative mechanisms. Future studies will address this challenging issue.

## Conclusions

Taken together, the results described herein on the oxidation of **S1** with hydrogen peroxide catalyzed by manganese complexes show how careful tuning of the catalyst structure and reaction conditions (solvent, temperature, and carboxylic acid) can be employed to exquisitely govern product selectivity among multiple reaction channels in a C(sp^3^)–H oxidation reaction.

From a mechanistic perspective, the results conclusively demonstrate that stereospecific C–H oxidation performed by these catalysts can take place via cationic intermediates and that a judicious choice of catalyst and reaction conditions makes this path exclusive. While the more electron-rich NMe_2_-substituted catalyst appears to favor radical over cationic mechanisms, a reversed scenario is attained with the more oxidizing TIPS-substituted catalyst. Cationic paths are also favored by the use of strong HBD solvents such as fluorinated alcohols, presumably because these interactions increase the oxidizing ability of the reactive manganese species while also stabilizing the cationic intermediates.

From a synthetically oriented perspective, the parallel outcome observed in the oxidation of **S1** and **S2** points toward the generality of these findings and, because of the straightforward access of spiro[2.5]octane structures from cyclohexanones, these results uncover the possibility of installing bicyclo[4.2.0]octan-1-ol structures in complex molecular settings using site-selective C–H bond functionalization.

Finally, it is also worth mentioning that as compared to **S2**, the results presented herein clearly show that **S1** represents an improved mechanistic probe to be employed in the study of C–H functionalization reactions. By deactivating proximal C–H bonds toward HAT and allowing discrimination between the axial and equatorial C–H bonds at C-4, the *tert*-butyl substituent imparts full control over site- and stereoselectivity. The formation of rearranged 6-*tert*-butylbicyclo[4.2.0]octane products can provide moreover conclusive information on the involvement of a cationic intermediate.

## Supporting Information Available

Experimental details for the preparation of metal complexes and substrates and catalytic reactions and details on isolation and characterization of the reaction products (PDF)
