# Supplementary material for: Resolving Oxygenation Pathways in Manganese-Catalyzed C(sp3)–H Functionalization via Radical and Cationic Intermediates
Source: J Am Chem Soc. 2022 Apr 13;144(16):7391–401. doi: 10.1021/jacs.2c01466 (PMC9052745; doi:10.1021/jacs.2c01466)
Supplement: Supplementary file 1 — ja2c01466_si_001.pdf [file ja2c01466_si_001.pdf]

Supporting information

Resolving Oxygenation Pathways in Manganese-Catalyzed C(*sp*^{3})–H Functionalization via Radical and Cationic Intermediates.

Marco Galeotti,[1] Laia Vicens,[2] Michela Salamone,[1] Miquel Costas,[2,*] and Massimo Bietti[1,*]

[1] *Dipartimento di Scienze e Tecnologie Chimiche, Università "Tor Vergata", Via della Ricerca Scientifica, 1 I-00133 Rome, Italy.*

[2] *QBIS Research Group, Institut de Química Computacional i Catàlisi (IQCC) and Departament de Química, Universitat de Girona, Campus Montilivi, Girona E-17071, Catalonia, Spain*

CONTENTS

1. Experimental Section **S3**
1.1. Instrumentation S3
1.2. Materials S3

2. Synthesis of the substrates **S4**

3. Synthesis of the complexes **S5**

4. Oxidation with H_2O_2 catalyzed by Mn complexes **S6**
4.1. General procedure S6
4.2. Optimization of the reaction conditions S6
4.2.1. Optimization of **S1** oxidation in MeCN S6
4.2.2. Optimization of **S1** oxidation in fluorinated solvents (HFIP and NFTBA) S11
4.2.3. Optimization of **S2** oxidation in HFIP S14

5. Isolation and characterization of the oxidation products **S15**
5.1. Scale-up oxidation of **S1** and **S2** S15
5.2. Acylation of **P1(2)a-OH** and **P1(2)b-OH** S18

6. ^{1}H-NMR and ^{13}C-NMR spectra **S22**

7. GC chromatograms of chiral products **S35**

8. References **S51**

1. Experimental section

1.1. Instrumentation

Gas-chromatographic analyses were carried out for the oxidation reaction with H_2O_2 catalyzed by manganese-oxo complexes using an Agilent 7820A gas chromatograph equipped with an HP-5 capillary column 30m x 0.32 mm x 0.25 μm and a flame ionizator detector. GC-MS analyses are performed on an Agilent 7890A gas chromatograph equipped with an HP-5MS capillary column (30 m x 0.25 mm x 0.25 μm) interfaced with an Agilent 5975X mass spectrometer. NH_3 was used as the ionization gas. NMR spectra were taken on a Bruker Ultrashield AVANCE III400 or on a Bruker Ultrashield ASCEND Nanobay spectrometer using standard conditions. Spectra were referenced to the residual proton solvents peaks or TMS (tetramethylsilane). High resolution mass spectra (HRMS) were recorded on a Bruker MicroTOF-Q IITM instrument with an ESI source and a quadrupole analyzer at Serveis Tecnics of the University of Girona. Samples were introduced into the mass spectrometer ion source by direct infusion through a syringe pump and were externally calibrated using sodium formate. Chromatographic resolution of enantiomers was performed on an Agilent GC-7820-A chromatograph using a CYCLOSIL-B column.

1.2. Materials

Reagents and solvents used are of commercially available reagent quality unless stated otherwise and are purchased from SDS, Aldrich, Scharlab and Fluorochem. Sigma-Aldrich HPLC-grade acetonitrile was employed for oxidation catalysis.

The hydrogen peroxide solutions employed in the oxidation reactions were prepared by diluting commercially available hydrogen peroxide (30% H_2O_2 solution in water, Aldrich) in MeCN and in fluorinated solvents to achieve a ~1.0 M final concentration. All reagents used are of the highest commercial quality available unless stated otherwise.

2. Synthesis of the substrates

4-*tert*-butylspiro[2.5]octane (**S1**) was synthetized over two steps according to a slight modification of reported procedures (**Scheme S2.1**).

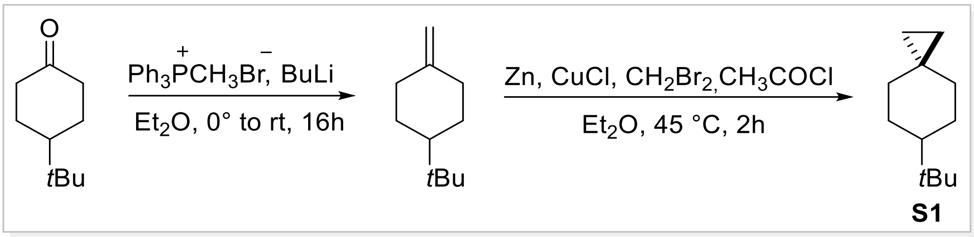

Scheme S1. Synthesis of 4-*tert*-butylspiro[2.5]octane (**S1**)

Wittig olefination of 4-tert-butylcyclohexanone[1]

13.7 g of methyltriphenylphosphonium bromide (0.0384 mol, 1.2 eq) and 100 mL of anhydrous diethyl ether were introduced in a 250 mL three-necked round-bottom flask equipped with a pressure-equalizing dropping funnel and kept under nitrogen. The reaction mixture was cooled at 0°C and 23 mL of 1.6 M *n*-butyllithium solution in hexane (0.0368 mol, 1.15 eq) were added dropwise in 15 minutes under vigorous stirring. When the solution becomes orange 5 g of 4-*tert*-butylcyclohexanone (0.032 mol, 1.0 eq) diluted in 10 mL of anhydrous diethyl ether, was added dropwise and the reaction mixture was kept stirring under a nitrogen atmosphere for 16 hours at 25 °C. After complete substrate conversion (monitored by TLC or GC analysis), the reaction mixture was cooled at 0°C, quenched with 100 mL of a saturated ammonium chloride aqueous solution and extracted with two portions of diethyl ether (2 x 75 mL). The combined organic phases are dried over anhydrous sodium sulfate, the solvent is removed at reduced pressure and the product is purified by flash chromatography over silica gel in pure *n*-hexane. 4.0 g of 4-*tert*-butylmethylenecyclohexane (0.026 mol, 81% yield) were collected as colorless liquid. Spectroscopic data match those previously reported.[1] ^{1}H-NMR (400 MHz, $CDCl_3$) δ, ppm: 4.58 (t, 2H), 2.36 – 2.30 (m, 2H), 1.99 (t, 2H), 1.87 (d, 2H), 1.17 –1.00 (m, 3H), 0.85 (s, 9H).

Simmons-Smith cyclopropanation of 4-tert-butylmethylenecyclohexane[2]

6.8 g of zinc (0.104 mol, 4 eq), 1.03 g of copper (I) chloride (0.0104 mol, 0.4 eq), 1.8 mL of dibromomethane (0.026 mol, 1.0 eq) and 15 mL of diethyl ether were introduced into a 100 mL three necked round-bottom flask, equipped with a condenser system and a pressure-equalizing dropping funnel. After addition of 148 µL of acetyl chloride (0.00208 mol, 0.08 eq), the flask was placed in a pre-heated oil bath (45-50 °C) under vigorous reflux and stirring. When the solution was turned dark (about 10 minutes), 4 g of 4-*tert*-butylmethylenecyclohexane (0.026 mol, 1.0 eq) diluted in 5 mL of diethyl ether, was added dropwise for 15 minutes. Additional 3.7 mL of dibromomethane (0.052 mol, 2.0 eq) diluted in 5 mL of diethyl ether were added dropwise for 30 minutes under manageable reflux. When substrate was fully converted (generally 2 hours after the addition of all the reagents), the flask was cooled in an ice bath and 25 mL of a saturated ammonium chloride aqueous solution were added dropwise (the reaction is strongly exothermic) through the dropping funnel. The residual solid zinc was removed by vacuum filtration and the aqueous layer of the filtrate was washed twice with *n*-pentane (2 x 25 ml). The combined organic layers were washed twice with a 2.5 M sodium hydroxide

aqueous solution (2 x 50 mL), one time with brine (50 mL) and were dried over anhydrous sodium sulfate. Due to product volatility *n*-pentane and diethyl ether were carefully removed by Hempel fractional distillation and a controlled vacuum distillation (110 °C, 60 mmHg) of the remaining oil gave the pure cyclopropyl derivative. 2.0 g of 4-*tert*-butylspiro[2.5]octane (**S1**, 0.012 mol, 46% yield) were collected as colorless liquid. Spectroscopic data match those previously reported.[3] ^{1}H-NMR (400 MHz, $CDCl_3$) δ, ppm: 1.80 – 1.68 (m, 4H), 1.22 – 1.08 (m, 2H), 1.07 – 0.95 (m, 1H), 0.89 (m, 11H), 0.32 – 0.23 (m, 2H), 0.23 – 0.12 (m, 2H). ^{13}C{^{1}H}-NMR (400 MHz, $CDCl_3$) δ, ppm: 48.0, 36.2, 32.5, 27.7, 26.5, 19.1, 12.4, 11.6.

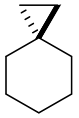

S2 Spiro[2.5]octane (**S2**) was synthetized via Simmons-Smith cyclopropanation of methylenecyclohexane according to the procedure reported above. Vacuum distillation (73 °C, 80 mmHg) of the crude afforded **S2** as colorless liquid (2.0 g, 0.018 mol, 43% yield). Spectroscopic data match those previously reported.[4] ^{1}H-NMR (400 MHz, $CDCl_3$) δ, ppm: 1.5 (m, 6H), 1.25 (m, 4H), 0.19 (m, 4H).

3. Synthesis of the complexes

The complexes [Mn(OTf)$_2$(mcp)],[5] [Mn(OTf)$_2$(^{TIPS}mcp)],[6] Mn(OTf)$_2$(^{CF_3}mcp)],[6] Mn(OTf)$_2$(pdp)],[7] [Mn(OTf)$_2$(^{TIPS}pdp)],[6] [Mn(OTf)$_2$(^{Bz}pdp)],[8] [Mn(OTf)$_2$(^{dMM}pdp)][9] and [Mn(OTf)$_2$(^{Me_2N}pdp)][9] were prepared according to the reported procedures (**Figure S1**).

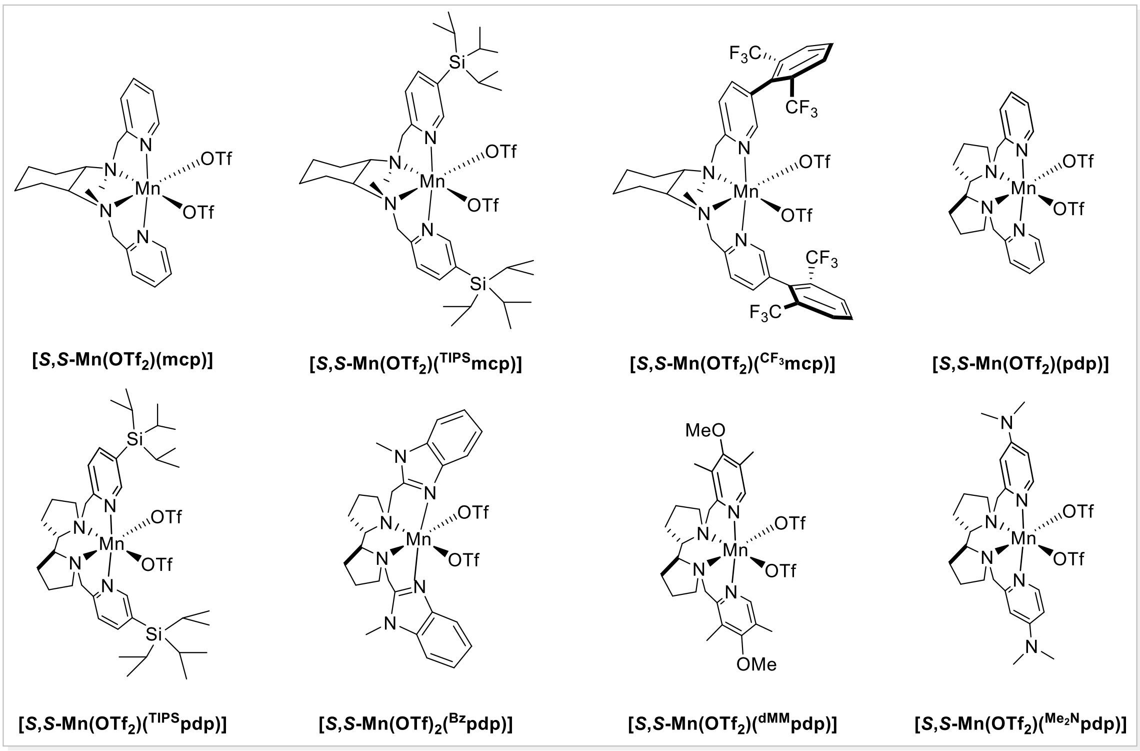

Figure S1. Structures of the manganese catalysts employed in this work.

4. Oxidation with H_2O_2 catalyzed by Mn complexes

4.1. General procedure

The following procedure was used for the aliphatic C–H bond oxidations of **S1** and **S2** with H_2O_2 catalyzed by Mn catalysts unless otherwise noted.

A MeCN, HFIP (1,1,1,3,3,3-hexafluoro-2-propanol) or NFTBA (nonafluoro-*tert*-butyl alcohol) solution (400 μL, 0.125 M) of the substrate (50 μmol, 1.0 eq) and the Mn catalyst (0.5 μmol, 1 mol%) was prepared in a 10 mL vial equipped with a stirring bar and thermostated at T = 0 °C (ice bath) or 25 °C. The carboxylic acid (15 eq) was added directly to the solution. Then ~1.0 M H_2O_2 solution in the pertinent solvent was directly added by syringe pump over 30 minutes. At this point 0.5 eq of internal standard (4-*tert*-butylcyclohexanone) was added and the solution was quickly filtered through a small silica and basic alumina plug, which was subsequently rinsed with 2 x 1 mL of EtOAc. GC analysis of the solution afforded the substrate conversions and product yields relative to the internal standard integration. Calibration curves were obtained using an authentic sample of the pure compounds prepared by scale-up catalysis or alternative procedures.

4.2. Optimization of the reaction conditions

Unless otherwise noted, the optimization experiments for the oxidation of 6-*tert*-butylspiro[2.5]octane (**S1**) were done following the general procedure reported above, using $Mn(OTf)_2(^{TIPS}mcp)$ as reference catalyst. Catalyst enantiomers were used interchangeably.

4.2.1. Optimization of S1 oxidation in MeCN

Table S1. Screening of the equivalent of H_2O_2.[a]

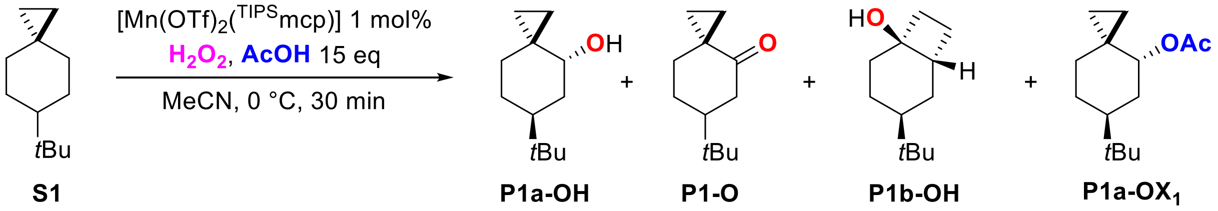

Entry	H_2O_2 eq	Conv. / %	Product yields / %				Total yield / %
			P1a-OH	P1-O	P1b-OH	P1a-OX1	
**1**	1.5	60	12	32	1.5	18	63.5
**2**	1.5+1.5[b]	90	2	54	2	24	82
**3**	**3.5**	**96**	**-**	**61**	**2**	**25**	**88**
**4[c]**	3.5	>99[d]	-	59	2	20	81

[a]Conversion and total yields were determined by GC and were averaged over at least two independent experiments. [b]Second addition of 1 mol% of $[Mn(OTf)_2(^{TIPS}mcp)]$, 1.5 eq of H_2O_2 and 15 eq of AcOH in further 30 min (60 min of total reaction time). [c]$[Mn(OTf)_2(^{TIPS}pdp)]$ 1 mol% was used. [d]Not identified byproducts in the chromatogram.

Table S2. Screening of the carboxylic acid co-catalysts.[a]

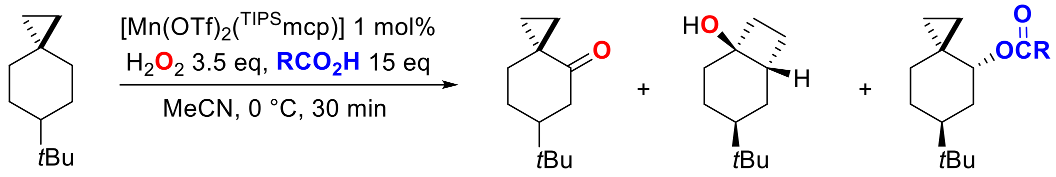

S1 P1-O P1b-OH P1a-OX$_n$

Entry	RCO₂H	Conv. / %	Product yields / %			Total yield / %
			P1-O	P1b-OH	P1a-OX$_n$	
**1**	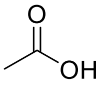	96	61	2	25	88
**2**	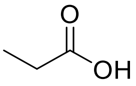	82	61	2	13	76
**3**	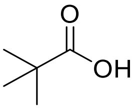	83	68	2	4	74
**4**	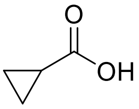	94	75	2	4	81
**5**	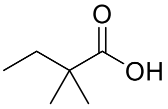	60	43.5	1.5	1	46
**6**[b]	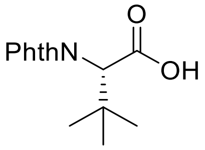	51	42	2	-	44

[a]Conversion and total yields were determined by GC and were averaged over at least two independent experiments. [b]1.0 eq of Phth-Tle-OH was used.

Table S3. Effect of the catalyst structure in MeCN.[a]

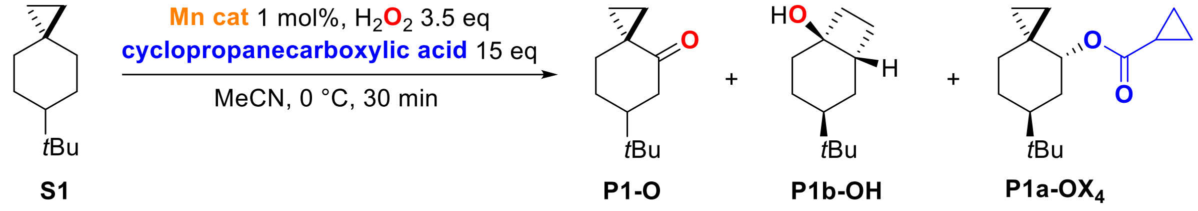

Entry	Mn cat	Conv. / %	Product yields / %			Total yield / %
			P1-O	P1b-OH	P1a-OX4	
1	[Mn(OTf)$_2$(mcp)]	75	58	3	3	64
**2**	**[Mn(OTf)$_2$(^{TIPS}mcp)]**	**94**	**75**	**2**	**4**	**81**
3	[Mn(OTf)$_2$(^{CF_3}mcp)]	46	22 (17)[b]	4	2	45
4	[Mn(OTf)$_2$(pdp)]	66	54	3	3	60
5	[Mn(OTf)$_2$(^{TIPS}pdp)]	85	62	3	4	69
6	[Mn(OTf)$_2$(^{Bz}pdp)]	87	62	3	5	70
7	[Mn(OTf)$_2$(^{dMM}pdp)]	80	60	1	6	67
**8**[c]	**[Mn(OTf)$_2$(^{Me_2N}pdp)]**	**66**	**53**	**-**	**6**	**59**

[a]Conversion and total yields were determined by GC and were averaged over at least two independent experiments. [b]Additional 17% yield of **P1a-OH**. [c]2.5 eq of H_2O_2 were used.

Table S4. Optimization of the **S1** oxidation employing Phth-Tle-OH as acid co-catalyst.[a]

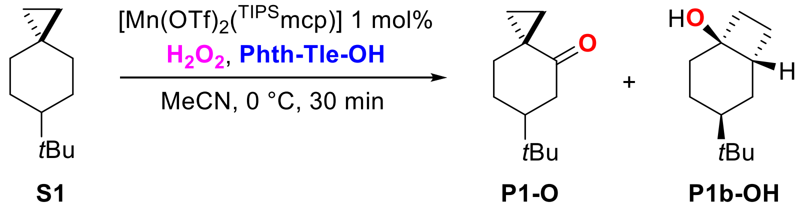

Entry	H_2O_2 / eq	Phth-Tle-OH / eq	Conv. / %	Product yields / %		Total yield / %
				P1-O	P1b-OH	
1	3.5	1.0	51	42	2	44
2[b]	5.0	1.0	50	47	2	49
3[c]	5.0	1.0	63	55	2.5	57.5
**4**[d]	**5.0**	**1.0+1.0+1.0**	**73**	**63.5**	**2.5**	**66**

[a]Conversion and total yields were determined by GC and were averaged over at least two independent experiments. [b]5 mol% of [Mn(OTf)$_2$(^{TIPS}mcp)] was used. [c]5 mol% of [Mn(OTf)$_2$(^{TIPS}mcp)] was used. Additional 5 mol% of catalyst was added after 10 and 20 min (15 mol% of [Mn(OTf)$_2$(^{TIPS}mcp)]). [e]5 mol% of [Mn(OTf)$_2$(^{TIPS}mcp)] was used. Additional 5 mol% of catalyst and 1.0 eq of Phth-Tle-OH were added after 10 and 20 min (15 mol% and 3.0 eq of [Mn(OTf)$_2$(^{TIPS}mcp)] and Phth-Tle-OH, respectively).

Table S5. Screening of the carboxylic acid amount.[a]

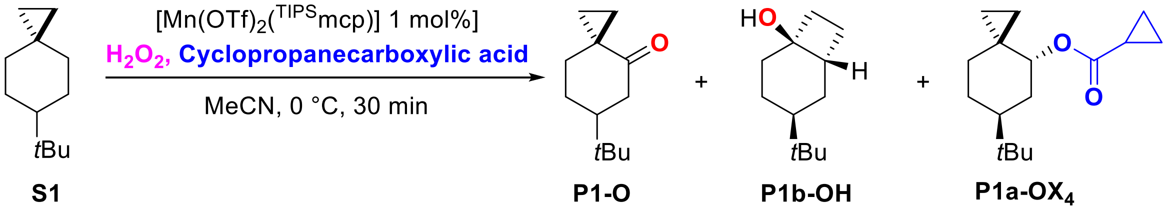

Entry	H_2O_2 / eq	Cyclop. / eq	Conv. / %	Product yields / %			P1-O / P1a-OX4	Total yield / %
				P1-O	P1b-OH	P1a-OX4		
**1**	3.5	15	94	75	2	4	19	81
**2**	5.0	7	91	73	3	4	18	80
**3**	10	1.5	88	70	3	4	18	77

[a]Conversion and total yields were determined by GC and were averaged over at least two independent experiments.

Table S6. Effect of the carboxylic acid employing [Mn(OTf)$_2$(^{Me_2N}pdp)] as catalyst.[a]

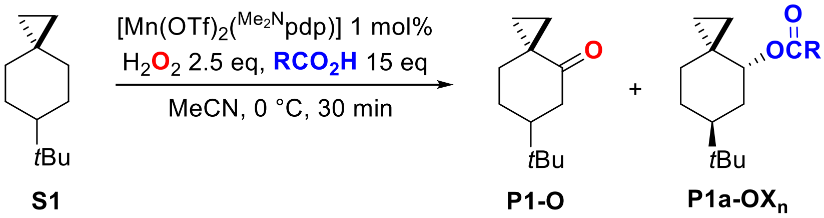

Entry	RCO_2H	Conv. / %	Product yields / %		Total yield / %
			P1-O	P1a-OXn	
**1**	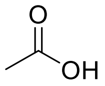	75	51	16	67
**2**	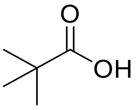	64	55	4	59
**3**	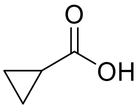	66	53	6	59
**4**[b]	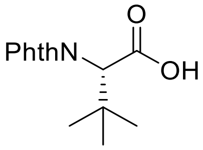	**68**	**61 (60)**[c]	**-**	**61 (60)**[c]

[a]Conversion and total yields were determined by GC and were averaged over at least two independent experiments. [b]3.5 eq of H_2O_2 and 1.0 eq of Phth-Tle-OH were used. [c]Isolated yield.

Table S7. Blank experiments.

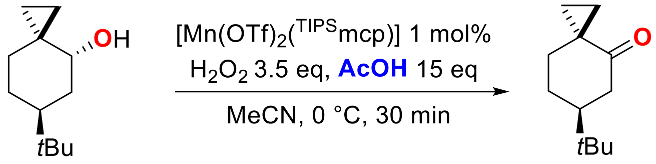

P1a-OH **P1-O**

Entry	Conv. / %	P1-O yield / %
**1**	74	66
**2**[a]	-	-

[a]Absence of [Mn(OTf)$_2$(^{TIPS}mcp)].

4.2.2. Optimization of S1 oxidation in fluorinated solvents (HFIP and NFTBA)

Table S8. Effect of the carboxylic acid and the temperature in HFIP.[a]

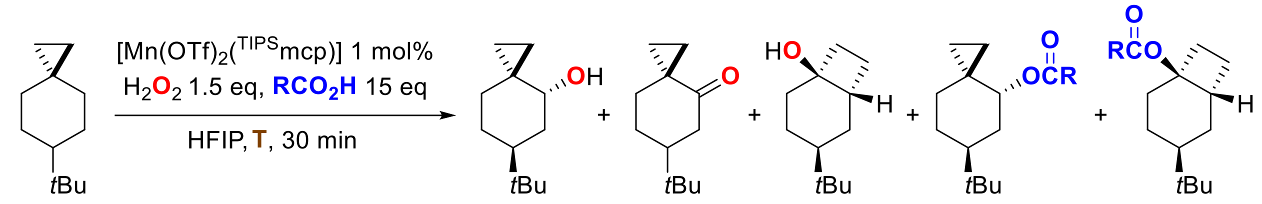

S1 P1a-OH P1-O P1b-OH P1a-OXn P1b-OXn

Entry	RCO2H	T / °C	Conv. / %	Product yields / % P1a-OH	P1-O	P1b-OH	P1a-OXn	P1b-OXn	Total yield / %
1	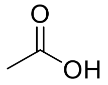	0	85	2	2	4	44	21	73
2		**25**	**90**	**-**	**-**	**3**	**41**	**33**	**77**
3	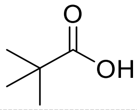	0	95	1	18	3	47	9	78
4		25	85	-	5	2	50	15	72
5	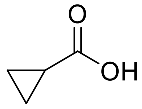	0	94	6	10	2	47	13	78
6		25	86	1.5	3	4	37	24	69.5

[a]Conversion and total yields were determined by GC and were averaged over at least two independent experiments.

Table S9. Effect of the carboxylic acid in NFTBA.[a]

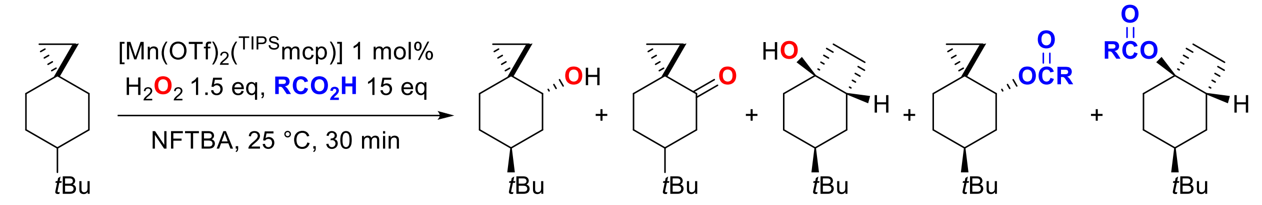

S1 P1a-OH P1-O P1b-OH P1a-OXn P1b-OXn

Entry	RCO2H	Conv. / %	Product yields / % P1a-OH	P1-O	P1b-OH	P1a-OXn	P1b-OXn	Total yield / %
1	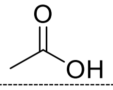	95[b]	10	2	5	34	9	60
2	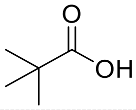	98	20	8	18	47	4	97
3	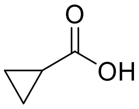	95	33	9	8	25	4	79

[a]Conversion and total yields were determined by GC and were averaged over at least two independent experiments. [b]Not identified byproducts in GC.

Table S10. Oxidation of **S1** in absence of carboxylic acid.[a]

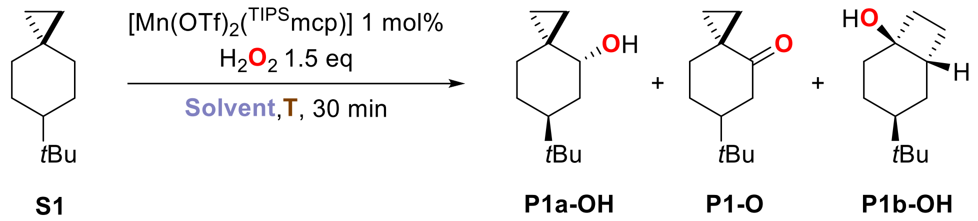

Entry	Solvent	T / °C	Conv. / %	Product yields / %			Total yield / %
				P1a-OH	P1-O	P1b-OH	
**1**	HFIP	0	23	8	3	11	22
**2**		**25**	**30**	1	**-**	**23**	**24**
**3**	NFTBA	0	50	14	4	19	37
**4**		25	52	11	-	31	42

[a]Conversion and total yields were determined by GC and were averaged over at least two independent experiments.

Table S11. Effect of the Ac-Gly-OH co-catalyst in HFIP.[a]

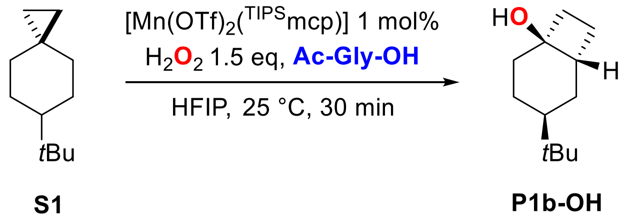

Entry	Ac-Gly-OH	Conv. / %	P1b-OH yield / %
**1**	-	30	23[b]
**2**	1.5 eq	93[c]	34
**3**	15 mol%	92[c]	47
**4**	**3 mol%**	**65**	**47 (47)[d]**
**5**	1.5 mol%	63	38

[a]Conversion and total yields were determined by GC and were averaged over at least two independent experiments. [b]Additional 1% yield of **P1a-OH**. [c]Not identified byproducts in GC. [d]Isolated yield.

Table S12. Oxidation of **S1** in absence of the carboxylic acid employing $[Mn(OTf)_2(^{Me_2N}pdp)]$ as catalyst.[a]

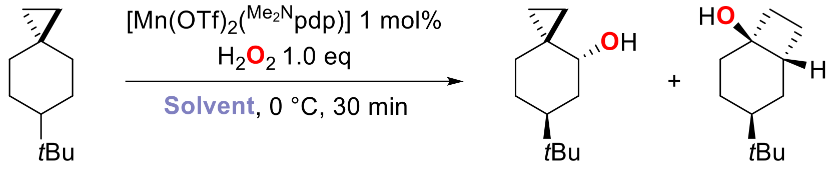

S1 **P1a-OH** **P1b-OH**

Entry	Solvent	Conv. / %	Product yields / %		Total yield / %
			P1a-OH	P1b-OH	
1	HFIP	57	50	5	55
2	NFTBA	52	46	-	46
3[b]		**68**	**62 (57)[c]**	-	**62 (57)[c]**

[a]Conversion and total yields were determined by GC and were averaged over at least two independent experiments. 1.5 eq H_2O_2 were used. [c]Isolated yield (0.656 and 3.6 mmol scale, see **Scheme 4c** in the manuscript).

Table S13. Yields and enantioselectivities on the oxidation of **S1** catalyzed by manganese complexes.[a]

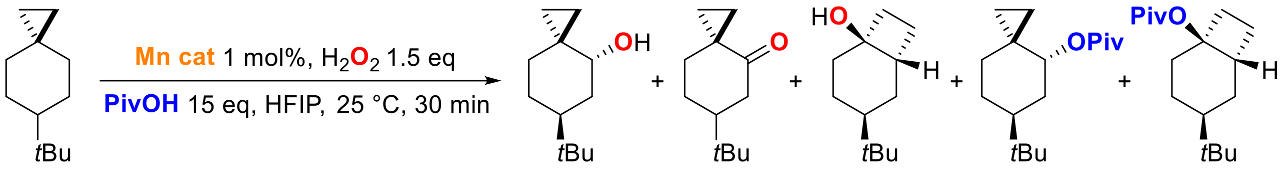

S1 **P1a-OH** **P1-O** **P1b-OH** **P1a-OX3** **P1b-OX3**

Entry	Mn cat[b]	Conv / %	(Product yields / %) ee / % [c]					Total yield / %
			P1a-OH	P1-O	P1b-OH	P1a-OX3	P1b-OX3	
1	$[Mn(OTf)_2(mcp)]$	71	-	(7)	(5) **24**	(44) **21**	(8) **17**	64
2	$[Mn(OTf)_2(^{TIPS}mcp)]$	85	-	(5)	(2) **3**	(50) **4**	(15) **5**	72
3	$[Mn(OTf)_2(^{CF_3}mcp)]$	93	-	-	(6) **53**	(53) **57**	(16) **56**	75
4	$[Mn(OTf)_2(pdp)]$	66	-	(6)	(4) **24**	(38) **22**	(6) **17**	54
5	$[Mn(OTf)_2(^{TIPS}pdp)]$	86	-	(4)	(3) **7**	(56) **6**	(10) **5**	73
6	$[Mn(OTf)_2(^{Bz}pdp)]$	80	-	(5)	(5) **35**	(53) **39**	(11) **38**	74
7	$[Mn(OTf)_2(^{dMM}pdp)]$	96	(33) **18**	(16)	(3) **18**	(33) **23**	(8) **20**	93
8	$[Mn(OTf)_2(^{Me_2N}pdp)]$	79	(38) **13**	(25)	(2) **23**	(9) **17**	(2) **22**	76
9[d]		28	(21) **16**	(1)	(1)	(3) **18**	(1)	27

[a]Conversion and total yields were determined by GC and were averaged over at least two independent experiments. [b]Catalyst structures are shown in **Figure S1**. [c]ee's were determinated by GC equipped with chiral stationary phase. Representative chromatograms of chiral products are displayed in **Figures S31-S58**. [d]0.5 eq of H_2O_2 was used.

4.2.3. Optimization of S2 oxidation in HFIP

Table S14. Effect of the catalyst structure in HFIP.[a]

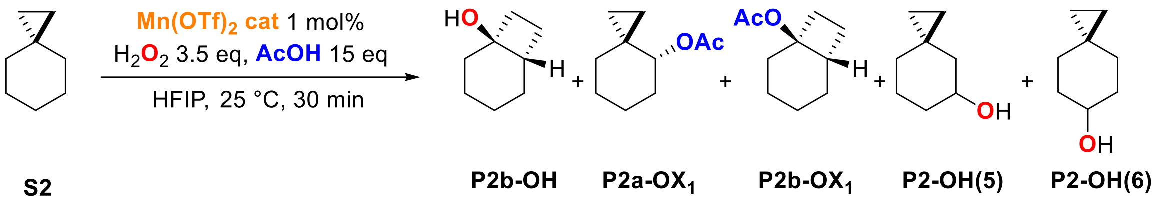

Entry	Mn cat	Conv. / %	Product yields / %					Total yield / %
			P2b-OH	P2a-OX1	P2b-OX1	P2-OH(5)	P2-OH(6)	
**1**	[Mn(^{TIPS}mcp)]	60	2	17	13	7	5	44
**2**	**[Mn(^{TIPS}pdp)]**	**80**	**2**	**26**	**19**	**11**	**8**	**66**

[a]Conversion and total yields were determined by GC and were averaged over at least two independent experiments.

Mn(OTf)$_2$(^{TIPS}pdp) was chosen as catalyst for the same reaction in HFIP replacing 15 eq of acetic acid with 3 mol% of Ac-Gly-OH (see **Scheme 5** in the manuscript).

5. Isolation and characterization of the oxidation products

Trans-6-*tert*-butylspiro[2.5]octan-4-ol (**P1a-OH**), *cis*-4-(*tert*-butyl)-bicyclo[4.2.0]octan-1-ol (**P1a-OH**) and 6-*tert*-butylspiro[2.5]octan-2-one (**P1-O**) obtained as single products in the oxidation of **S1** with H_2O_2 catalyzed by Mn catalysts, were prepared by scale-up oxidation reaction. The oxidation products of **S2**, spiro[2.5]octan-4-ol (**P2a-OH**), *cis*-bicyclo[4.2.0]octan-1-ol (**P2b-OH**), spiro[2.5]octan-4-one (**P2-O**), spiro[2.5]octan-5-ol (**P2-OH(5)**), spiro[2.5]octan-5-one (**P2-O(5)**), spiro[2.5]octan-6-ol (**P2-OH(6)**), and spiro [2.5]octan-6-one (**P2-O(6)**) were also isolated by scale-up catalysis. The other ester products derived by oxidation of **S1** and **S2** were instead prepared by esterification of the alcohols **P1(2)a-OH** and **P1(2)b-OH**.

5.1. Scale-up oxidation of S1 and S2

General procedure. A MeCN, HFIP or NFTBA (volume range 2.5-4 mL) solution of substrate (1.0 eq, 0.125 M), Mn catalyst (1 mol%) was prepared in a 25 mL round bottom flask equipped with a magnetic stirring bar. When used, the carboxylic acid (15 eq) or the amino acid (range 0.03 – 1.0 eq) were added and the resulting mixture was cooled at 0 °C (ice bath) or 25 °C. Then ~1.0 M H_2O_2 solution in the pertinent solvent (range 1.0 – 3.5 eq) was directly added by syringe pump over 30 minutes. Then the solvent was evaporated at reduced pressure and the crude was rinsed with 5 mL of CH_2Cl_2. The resulting organic solution was extracted two times with 2 x 5 mL saturated $NaHCO_3$ aqueous solution and one time with 5 mL of brine. The reunited organic phases were dried with $MgSO_4$, filtered and then evaporated at reduced pressure. The resulting residue was purified by flash chromatography on silica gel and the spectroscopic data of the pure products were compared with those reported in literature. The characterization of the unknown products was performed by ^{1}H-NMR, ^{13}C-NMR and HRMS.

Reactions were performed on a 0.328 – 0.656 mmol scale of **S1** and **S2**.

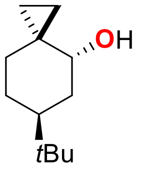

***Trans*-6-*tert*-butylspiro[2.5]octan-4-ol (P1a-OH)**. Prepared following the general procedure on 0.656 mmol of **S1**. General conditions: 5 mg of [(*S*,*S*)-Mn(^{Me_2N}pdp)] (0.00656 mmol, 1 mol%), 109.1 mg of **S1** (0.656 mmol, 1.0 eq), 1.63 mL of 0.603 M H_2O_2 solution in NFTBA (0.984 mmol, 1.5 eq), 4 mL of NFTBA at 0 °C for 30 min. Purification by flash chromatography over silica gel (hexane-EtOAc 10:1) afforded 68.1 mg of *trans*-6-*tert*-butylspiro[2.5]octan-4-ol (**P1a-OH**, 0.374 mmol, 57% yield) as white solid. Spectroscopic data match those previously reported.[10] ^{1}H-NMR (400 MHz, $CDCl_3$) δ, ppm: 3.09 (s, 1H), 2.16 – 2.06 (m, 1H), 1.91 (dq, *J* = 13.3, 3.0 Hz, 1H), 1.75 (ddd, *J* = 12.2, 6.4, 3.4 Hz, 1H), 1.59 – 1.46 (m, 2H), 1.35 (td, *J* = 13.1, 2.8 Hz, 1H), 1.15 (qd, *J* = 12.5, 3.7 Hz, 1H), 0.89 (s, 9H), 0.80 – 0.72 (m, 1H), 0.47 – 0.41 (m, 1H), 0.36 – 0.27 (m, 3H). ^{13}C{^{1}H}-NMR (400 MHz, $CDCl_3$) δ, ppm: 75.8, 40.5, 32.7, 32.1, 30.5, 27.5, 26.0, 23.5, 11.7, 10.3. This compound was also prepared on *3.6 mmol* (655 mg) of **S1**. The same value of isolated yield (57%) was observed.

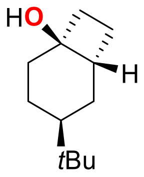

***Cis*-4-(*tert*-butyl)-bicyclo[4.2.0]octan-1-ol (P1b-OH)**. Prepared following the general procedure on 0.505 mmol of **S1**. General conditions: 5 mg of [Mn(OTf)$_2$(^{TIPS}mcp)] (0.00505 mmol, 1 mol%), 84 mg of **S1** (0.505 mmol, 1.0 eq), 1.8 mg of Ac-Gly-OH (0.0152 mmol, 3 mol%), 0.842 ml of 0.9 M H_2O_2 solution in HFIP (0.758 mmol, 1.5 eq), 4 mL of HFIP at 25 °C for 30 min. Purification by flash chromatography over silica gel (hexane-EtOAc 15:1) afforded 43 mg of *cis*-4-(*tert*-butyl)-bicyclo[4.2.0]octan-1-ol (**P1b-OH**, 0.236 mmol, 47% yield) as white solid. ^{1}H-NMR (400 MHz, $CDCl_3$) δ, ppm: δ 2.38 (ddd, *J* = 10.7, 8.9, 6.1 Hz, 1H), 1.92 – 1.84 (m, 1H), 1.82 – 1.68 (m, 4H), 1.57 (dddd, *J* = 12.5, 5.9, 3.8, 1.9 Hz, 2H), 1.48 (ddt, *J* = 13.6, 3.7, 1.8 Hz, 1H), 1.38 – 1.32 (m, 1H), 1.32 – 1.27 (m, 1H), 1.16 (tdd, *J* = 12.2, 3.9, 2.1 Hz, 1H), 1.10 – 0.99 (m, 1H), 0.86 (s, 9H). ^{13}C{^{1}H}-NMR (400 MHz, $CDCl_3$) δ, ppm: 71.6, 44.9, 43.6, 36.3, 36.3, 35.7, 32.3, 27.5, 25.2, 22.7, 15.8, 14.1. HRMS (ESI-MS) *m/z* calculated for $C_{12}H_{22}O$ $[M+Na]^+$ 205.1563, found 205.1561. The assignment of stereochemistry was carried out by NOESY- and selective NOE-NMR spectra (see **Figures S6-S8** for details).

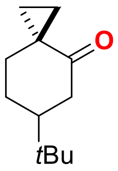

6-*tert*-butylspiro[2.5]octan-4-one (P1-O). Prepared following the general procedure on 0.328 mmol of **S1**. General conditions: 2.5 mg of [Mn(OTf)$_2$(^{Me_2N}pdp)] (0.00328 mmol, 1 mol%), 54.6 mg of **S1** (0.328 mmol, 1.0 eq), 85.7 mg of Phth-Tle-OH (0.328 mmol, 1.0 eq), 1.0 ml of 1.2 M H_2O_2 solution in MeCN (1.2 mmol, 3.5 eq), 2 ml of MeCN at 0 °C for 30 min. Purification by flash chromatography over silica gel (hexane-EtOAc 20:1) afforded 35.6 mg of 6-*tert*-butylspiro[2.5]octan-4-one (**P1-O**, 0.198 mmol, 60% yield) as colorless liquid. Spectroscopic data match those previously reported.[10] ^{1}H-NMR (400 MHz, $CDCl_3$) δ, ppm: δ 2.54 (ddd, *J* = 16.4, 4.5, 2.2 Hz, 1H), 2.14 (dd, *J* = 16.4, 12.7 Hz, 1H), 2.04 – 1.89 (m, 2H), 1.72 – 1.63 (m, 1H), 1.56 (ddd, *J* = 13.8, 7.0, 3.7 Hz, 1H), 1.51 – 1.42 (m, 2H), 0.92 (s, 10H), 0.69 (ddd, *J* = 9.5, 6.5, 3.1 Hz, 1H), 0.58 (ddd, *J* = 9.0, 6.6, 3.7 Hz, 1H). ^{13}C{^{1}H}-NMR (400 MHz, $CDCl_3$) δ, ppm: 212.6, 46.4, 41.8, 33.3, 32.6, 28.3, 27.0, 25.0, 21.6, 14.0.

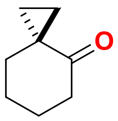

Spiro[2.5]octan-4-one (P2-O). Prepared following the general procedure on 0.656 mmol of **S2**. General conditions: 5 mg of [Mn(OTf)$_2$(^{Me_2N}pdp)] (0.00656 mmol, 1 mol%), 72.3 mg of **S2** (0.656 mmol, 1.0 eq), 171.4 mg of Phth-Tle-OH (0.656 mmol, 1.0 eq), 1.5 ml of 1.5 M H_2O_2 solution in MeCN (2.3 mmol, 3.5 eq), 4 ml of MeCN at 0 °C for 30 min. Purification by flash chromatography over silica gel (hexane-EtOAc 20:1) afforded 19.4 mg of spiro[2.5]octan-4-one (**P2-O**, 0.16 mmol, 24% yield) as colorless liquid. 4.2 mg and 3.4 mg of spiro[2.5]octan-5-one (**P2-O(5)**, 0.034 mmol, 5.2% yield) and spiro[2.5]octan-6-one (**P2-O(6)**, 0.027 mmol, 4.1% yield) respectively were also collected. Spectroscopic data match those previously reported.[11]

P2-O: ^{1}H-NMR (400 MHz, $CDCl_3$) δ, ppm: 2.42 (t, *J* = 6.7 Hz, 2H), 1.84 (m, 2H), 1.83 – 1.75 (m, 2H), 1.65 (dd, *J* = 5.6, 5.9 Hz, 2H), 1.10 (dd, *J* = 3.5, 5.9 Hz, 2H), 0.47 (dd, *J* = 3.5, 5.9 Hz, 2H).
P2-O(5): ^{1}H-NMR (400 MHz, $CDCl_3$) δ, ppm: 2.36 (t, *J* = 6.7 Hz, 2H), 2.14 (s, 2H), 1.93 (m, 2H), 1.54 (m, 2H), 0.34 (m, 4H).
P2-O(6): ^{1}H-NMR (400 MHz, $CDCl_3$) δ, ppm: 2.46 – 2.38 (m, 4H), 1.68 (t, *J* = 6.6 Hz, 4H), 0.49 (s, 4H).

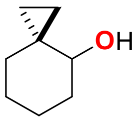

Spiro[2.5]octan-4-ol (P2a-OH). Prepared following the general procedure on 0.328 mmol of **S2**. General conditions: 2.5 mg of [Mn(OTf)$_2$(^{Me_2N}pdp)] (0.00328 mmol, 1 mol%), 36.1 mg of **S2** (0.328 mmol, 1.0 eq), 0.82 ml of 0.6 M H_2O_2 solution in NFTBA (0.492 mmol, 1.5 eq), 2 ml of NFTBA at 0 °C for 30 min. Purification by flash chromatography over silica gel (hexane-EtOAc 10:1) afforded 10.2 mg of spiro[2.5]octan-4-ol (**P2a-OH**, 0.081 mmol, 25% yield) as colorless liquid. Spectroscopic data match those previously reported.[12] ^{1}H-NMR (400 MHz, $CDCl_3$) δ, ppm: 3.20 (m, 1H), 1.79 – 1.64 (m, 4H), 1.56 (tt, *J* = 5.5, 3.1 Hz, 1H), 1.53 – 1.40 (m, 2H), 1.34 (s, 1H), 0.97 (m, 1H), 0.50 – 0.34 (m, 2H), 0.32 – 0.17 (m, 2H).
6.3 mg of an inseparable mixture of **P2-OH(5)** and **P2-OH(6)** (0.050 mmol, 15% yield) were oxidized by chromic acid according to a reported procedure.[13] The spectroscopic data of the obtained products match those of **P2-O(5)** and **P2-O(6)** reported above.

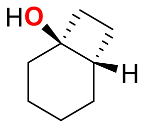

***Cis*-bicyclo[4.2.0]octan-1-ol (P2b-OH)**. Prepared following the general procedure on 0.505 mmol of **S2**. General conditions: 5 mg of [Mn(OTf)$_2$(^{TIPS}mcp)] (0.00505 mmol, 1 mol%), 55.6 mg of **S2** (0.505 mmol, 1.0 eq), 1.8 mg of Ac-Gly-OH (0.0152 mmol, 3 mol%), 0.758 ml of 1.0 M H_2O_2 solution in HFIP (0.758 mmol, 1.5 eq), 4 ml of HFIP at 25 °C for 30 min. Purification by flash chromatography over silica gel (hexane-EtOAc 15:1) afforded 15.3 mg of *cis*-bicyclo[4.2.0]octan-1-ol (**P2b-OH**, 0.121 mmol, 24% yield) as white solid. Spectroscopic data match those previously reported.[14] ^{1}H-NMR (400 MHz, $CDCl_3$) δ, ppm: 2.27 (dt, *J* = 15.3, 7.9 Hz, 1H), 1.89 – 1.70 (m, 3H), 1.57 (qq, *J* = 13.9, 8.2, 7.7 Hz, 6H), 1.35 (dtd, *J* = 26.7, 11.2, 7.1 Hz, 4H). ^{13}C-NMR (400 MHz, $CDCl_3$) δ, ppm: 72.1, 43.7, 36.2, 35.4, 24.2, 21.6, 21.2, 15.8.

5.2. Acylation of alcohols P1(2)a-OH and P1(2)b-OH

The ester products (**P1(2)a-OX$_n$** and **P1(2)b-OX$_n$**) were prepared according to a slightly modified reported procedure (**Scheme S3**).[15]

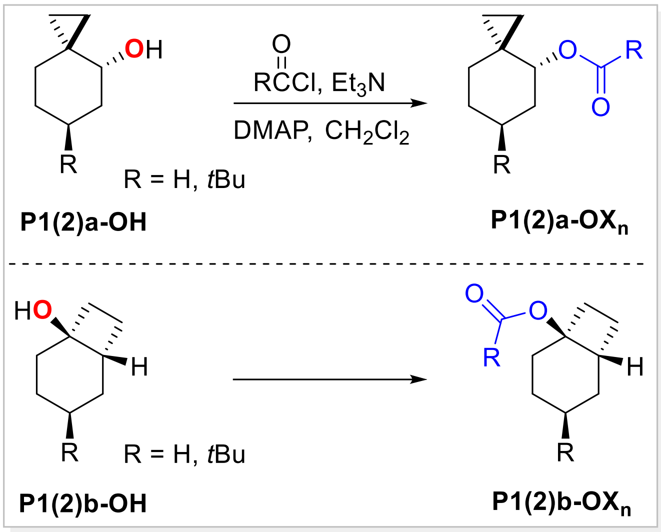

Scheme S3. Synthesis of products **P1(2)a-OX$_n$** and **P1(2)b-OX$_n$**.

General procedure. 0.15 mmol of the alcohols (1.0 eq), 1.5 eq of triethylamine, 5 mol% of 4-dimethylaminopyridine and 2.5 ml of CH_2Cl_2 were introduced in a 10 mL vial and kept under nitrogen. 1.5 eq of the corresponding acyl chloride were then added dropwise to the reaction mixture at 0 °C under magnetic stirring. After the addition, the solution was brought at room temperature and kept under stirring for 4 hours. The reaction mixture was then quenched with 2.5 mL of a 2 M HCl solution, and the organic layer was separated. Thus 2.5 mLof saturated $NaHCO_3$ aqueous solution were then added and the solution was kept under stirring for 30 min to remove the excess of the acyl chloride. At this point, the organic phase was extracted with 2 x 2.5 mL of saturated $NaHCO_3$ aqueous solution, 2 x 2.5 mL of brine and then dried over $MgSO_4$. After filtration, the solution was concentrated under vacuum and the crude was purified by flash chromatography on silica gel. The characterization of the products was accomplished by ^{1}H-NMR, ^{13}C-NMR and HMRS.

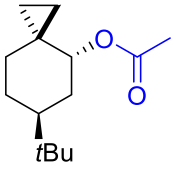

***Trans*-6-*tert*-butylspiro[2.5]octan-4-yl acetate (P1a-OX$_1$)**. Purification by flash chromatography (hexane-EtOAc 20:1) afforded 24.7 mg of *trans*-6-*tert*-butylspiro[2.5]octan-4-yl acetate (**P1a-OX$_1$**, 0.11 mmol, 73% yield) as colorless liquid. ^{1}H-NMR (400 MHz, $CDCl_3$) δ, ppm: 4.31 (m, 1H), 2.16 – 2.06 (m, 4H), 1.93 (ddd, 1H), 1.78 (m, 1H), 1.47 – 1.33 (m, 2H), 1.18 (qd, 1H), 0.88 (s, 9H), 0.79 (dtd, 1H), 0.61 – 0.50 (m, 1H), 0.40 – 0.29 (m, 3H). ^{13}C{^{1}H}-NMR (400 MHz, $CDCl_3$) δ, ppm: 170.9, 78.2, 41.5, 32.1, 31.3, 30.9, 27.6, 27.5, 25.8, 21.4, 11.8, 11.0. HRMS (ESI-MS) *m/z* calculated for $C_{14}H_{24}O_2$ $[M+Na]^+$ 247.1669, found 247.1675.

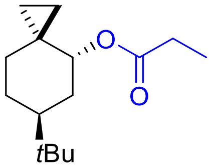

***Trans*-6-*tert*-butylspiro[2.5]octan-4-yl propionate (P1a-OX$_2$)**. Purification by flash chromatography (hexane-EtOAc 20:1) afforded 19.7 mg of *trans*-6-*tert*-butylspiro[2.5]octan-4-yl propionate (**P1a-OX$_2$**, 0.083 mmol, 55% yield) as colorless liquid. ^{1}H NMR (400 MHz, CDCl$_3$) δ, ppm: 4.31 (dq, *J* = 3.1, 1.5 Hz, 1H), 2.37 (q, *J* = 7.6 Hz, 2H), 2.16 – 2.01 (m, 1H), 1.92 (ddt, *J* = 12.9, 4.1, 2.2 Hz, 1H), 1.77 (dp, *J* = 12.2, 3.0 Hz, 1H), 1.45 – 1.32 (m, 2H), 1.18 (t, *J* = 7.6 Hz, 4H), 0.87 (s, 9H), 0.79 (dtd, *J* = 13.3, 3.6, 1.4 Hz, 1H), 0.57 – 0.52 (m, 1H), 0.42 – 0.28 (m, 3H). ^{13}C{^{1}H}-NMR (400 MHz, CDCl$_3$) δ, ppm: 174.2, 77.9, 41.6, 32.1, 31.4, 31.0, 28.2, 27.5, 25.8, 21.5, 11.7, 11.0, 9.5. HRMS (ESI-MS) *m/z* calculated for $C_{15}H_{26}O_2$ [M+Na]$^+$ 261.1825, found 261.1820.

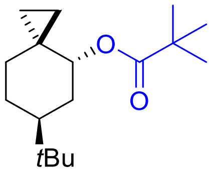

***Trans*-6-*tert*-butylspiro[2.5]octan-4-yl pivalate (P1a-OX$_3$)**. Purification by flash chromatography (hexane-EtOAc 20:1) afforded 17.5 mg of *trans*-6-*tert*-butylspiro[2.5]octan-4-yl pivalate (**P1a-OX$_3$**, 0.066 mmol, 44% yield) as colorless liquid. ^{1}H NMR (400 MHz, CDCl$_3$) δ, ppm: 4.27 (dq, *J* = 3.1, 1.4 Hz, 1H), 2.09 (tdd, *J* = 13.2, 3.7, 1.7 Hz, 1H), 1.90 (ddt, *J* = 13.0, 4.1, 2.1 Hz, 1H), 1.78 (dp, *J* = 12.2, 3.0 Hz, 1H), 1.47 – 1.32 (m, 2H), 1.30 – 1.17 (m, 10H), 0.87 (m, 10H), 0.56 – 0.48 (m, 1H), 0.41 – 0.26 (m, 3H). ^{13}C{^{1}H}-NMR (400 MHz, CDCl$_3$) δ, ppm: 178.0, 77.7, 41.9, 39.0, 32.0, 31.6, 31.0, 27.5, 27.3, 25.8, 21.4, 11.6, 10.7. HRMS (ESI-MS) *m/z* calculated for $C_{17}H_{30}O_2$ [M+Na]$^+$ 289.2138, found 289.2141.

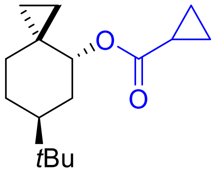

***Trans*-6-*tert*-butylspiro[2.5]octan-4-yl cyclopropanecarboxylate (P1a-OX$_4$)**. Purification by flash chromatography (hexane-EtOAc 20:1) afforded 24.4 mg of *trans*-6-*tert*-butylspiro[2.5]octan-4-yl cyclopropanecarboxylate (**P1a-OX$_4$**, 0.097 mmol, 65% yield) as colorless liquid. ^{1}H NMR (400 MHz, CDCl$_3$) δ, ppm: 4.30 (m, 1H), 2.12 – 2.05 (m, 1H), 1.96 – 1.87 (m, 1H), 1.83 – 1.73 (m, 1H), 1.70 – 1.61 (m, 1H), 1.48 – 1.31 (m, 2H), 1.18 (qd, *J* = 12.7, 3.7 Hz, 1H), 1.06 – 0.97 (m, 2H), 0.88 (s, 12H), 0.62 – 0.49 (m, 1H), 0.39 – 0.26 (m, 3H). ^{13}C{^{1}H}-NMR (400 MHz, CDCl$_3$) δ, ppm: 174.4, 78.0, 41.6, 32.1, 31.4, 31.0, 27.5, 25.8, 21.5, 13.4, 11.7, 11.0, 8.2. HRMS (ESI-MS) *m/z* calculated for $C_{16}H_{26}O_2$ [M+Na]$^+$ 273.1825, found 273.1827.

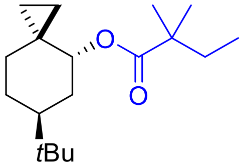

***Trans*-6-*tert*-butylspiro[2.5]octan-4-yl 2,2-dimethylbutanoate (P1a-OX$_5$)**. Purification by flash chromatography (pentane-EtOAc 50:1) afforded 22.9 mg of *trans*-6-*tert*-butylspiro[2.5]octan-4-yl 2,2-dimethylbutanoate (**P1a-OX$_5$**, 0.082 mmol, 55% yield) as colorless liquid. ^{1}H NMR (400 MHz, CDCl$_3$) δ, ppm: 4.30 (m, 1H), 2.08 (tdd, *J* = 13.2, 3.5, 1.6 Hz, 1H), 1.90

(dt, J = 10.4, 2.2 Hz, 1H), 1.78 (dp, J = 11.8, 3.0 Hz, 1H), 1.66 – 1.55 (m, 3H), 1.46 – 1.31 (m, 2H), 1.19 (s, 7H), 0.88 (d, J = 7.4 Hz, 11H), 0.81 (ddd, J = 13.4, 4.4, 2.2 Hz, 1H), 0.54 – 0.48 (m, 1H), 0.42 – 0.26 (m, 3H). $^{13}C\{^1H\}$-NMR (400 MHz, $CDCl_3$) δ, ppm: 177.4, 77.5, 42.9, 41.8, 33.5, 32.1, 31.6, 31.1., 27.5, 25.8, 24.9, 24.8, 21.5, 11.7, 10.8, 9.4. HRMS (ESI-MS) *m/z* calculated for $C_{18}H_{32}O_2$ $[M+Na]^+$ 303.2295, found 303.2297.

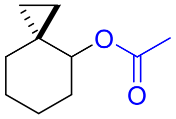

Spiro[2.5]octan-4-yl acetate (P2a-OX$_1$). Prepared following the general procedure on 0.1 mmol of **P2a-OH**. Purification by flash chromatography (hexane-EtOAc 20:1) afforded 8.2 mg of spiro[2.5]octan-4-yl acetate (**P2a-OX$_1$**, 0.049 mmol, 49% yield) as colorless liquid. ^1H-NMR (400 MHz, $CDCl_3$) δ, ppm: 4.39 (t, J = 4.0 Hz, 1H), 2.07 (s, 3H), 1.84 – 1.62 (m, 5H), 1.55 – 1.43 (m, 2H), 0.99 (d, J = 13.1 Hz, 1H), 0.59 – 0.52 (m, 1H), 0.45 – 0.36 (m, 1H), 0.36 – 0.19 (m, 2H). $^{13}C\{^1H\}$-NMR (400 MHz, $CDCl_3$) δ, ppm: 170.8, 32.1, 30.2, 29.7, 24.7, 21.6, 21.5, 21.4, 10.6, 10.3.

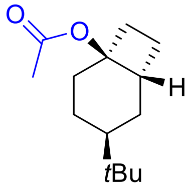

***Cis*-4-*tert*-butylbicyclo[4.2.0]octan-1-yl acetate (P1b-OX$_1$)**. Purification by flash chromatography (hexane-EtOAc 20:1) afforded 14.5 mg of *cis*-4-*tert*-butylbicyclo[4.2.0]octan-1-yl acetate **(P1b-OX$_1$**, 0.065 mmol, 43% yield) as colorless liquid. ^1H NMR (400 MHz, $CDCl_3$) δ, ppm: 2.63 – 2.53 (m, 1H), 2.34 (dt, J = 15.2, 4.1 Hz, 1H), 2.21 – 2.14 (m, 1H), 2.00 (s, 4H), 1.77 – 1.65 (m, 2H), 1.58 – 1.43 (m, 3H), 1.37 – 1.26 (m, 1H), 1.22 – 1.14 (m, 1H), 1.02 – 0.90 (m, 1H), 0.87 (s, 9H). $^{13}C\{^1H\}$-NMR (400 MHz, $CDCl_3$) δ, ppm: 170.0, 78.3, 43.3, 41.4, 34.0, 32.7, 32.3, 27.4, 25.4, 22.3, 21.9, 17.9. HRMS (ESI-MS) *m/z* calculated for $C_{14}H_{24}O_2$ $[M+Na]^+$ 247.1669, found 247.1659.

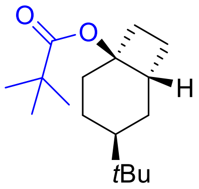

***Cis*-4-*tert*-butylbicyclo[4.2.0]octan-1-yl pivalate (P1b-OX$_3$)**. Purification by flash chromatography (hexane-EtOAc 20:1) afforded 11.9 mg of *cis*-4-*tert*-butylbicyclo[4.2.0]octan-1-yl pivalate **(P1b-OX$_3$**, 0.045 mmol, 30% yield) as colorless liquid. ^1H NMR (400 MHz, $CDCl_3$) δ, ppm: 2.66 – 2.50 (m, 1H), 2.34 – 2.24 (m, 1H), 2.23 – 2.12 (m, 1H), 2.08 – 1.91 (m, 1H), 1.80 – 1.64 (m, 2H), 1.54 – 1.46 (m, 2H), 1.34 (dd, J = 12.4, 6.5 Hz, 4H), 1.19 (s, 9H), 0.87 (s, 9H). $^{13}C\{^1H\}$-NMR (400 MHz, $CDCl_3$) δ, ppm: 177.5, 77.8, 43.4, 41.2, 38.7, 34.0, 32.7, 32.3, 29.7, 27.4, 27.1, 25.5, 22.4, 18.0. HRMS (ESI-MS) *m/z* calculated for $C_{17}H_{30}O_2$ $[M+Na]^+$ 289.2138, found 289.2147.

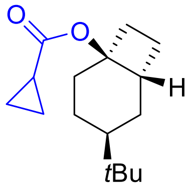

***Cis*-4-*tert*-butylbicyclo[4.2.0]octan-1-yl cyclopropanecarboxylate (P1b-OX$_4$)**. Purification by flash chromatography (hexane-EtOAc 20:1) afforded 13.8 mg of *cis*-4-*tert*-

butylbicyclo[4.2.0]octan-1-yl cyclopropanecarboxylate **(P1b-OX$_4$**, 0.055 mmol, 37% yield) as colorless liquid. ^{1}H NMR (400 MHz, $CDCl_3$) δ, ppm: 2.61 (td, *J* = 9.8, 6.1 Hz, 1H), 2.32 (dtd, *J* = 15.2, 3.4, 1.7 Hz, 1H), 2.15 (ddd, *J* = 11.2, 7.6, 1.7 Hz, 1H), 2.09 – 1.98 (m, 1H), 1.79 – 1.64 (m, 2H), 1.60 – 1.49 (m, 3H), 1.39 – 1.27 (m, 2H), 1.19 (tdd, *J* = 12.4, 4.0, 2.3 Hz, 1H), 1.04 – 0.97 (m, 1H), 0.95 (dt, *J* = 4.3, 3.2 Hz, 2H), 0.88 (s, 9H), 0.82 (dt, *J* = 8.1, 3.3 Hz, 2H). ^{13}C{^{1}H}-NMR (400 MHz, $CDCl_3$) δ, ppm: 173.7, 78.2, 43.4, 41.4, 34.1, 32.8, 32.3, 27.4, 25.4, 22.4, 17.9, 13.4, 8.1, 8.0. HRMS (ESI-MS) *m/z* calculated for $C_{16}H_{26}O_2$ $[M+Na]^+$ 273.1825, found 273.1830.

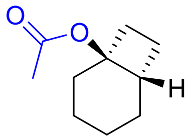

***Cis*-bicyclo[4.2.0]octan-1-yl acetate (P2b-OX$_1$)**. Prepared following the general procedure on 0.09 mmol of **P2b-OH**. Purification by flash chromatography (hexane-EtOAc 20:1) afforded 4.3 mg of *cis*-bicyclo[4.2.0]octan-1-yl acetate **(P2b-OX$_1$**, 0.026 mmol, 29% yield) as colorless liquid. Spectroscopic data match those previously reported.[14] ^{1}H NMR (400 MHz, $CDCl_3$) δ, ppm: 2.52 (q, *J* = 9.4, 8.9 Hz, 1H), 2.41 – 2.22 (m, 4H), 2.15 – 2.03 (m, 1H), 1.80 – 1.67 (m, 3H), 1.65 – 1.59 (m, 2H), 1.55 – 1.36 (m, 5H). ^{13}C{^{1}H}-NMR (400 MHz, $CDCl_3$) δ, ppm: 163.2, 79.0, 40.3, 34.4, 29.7, 24.2, 21.3, 21.1, 18.0, 17.9.

6. ^{1}H-NMR and ^{13}C-NMR spectra

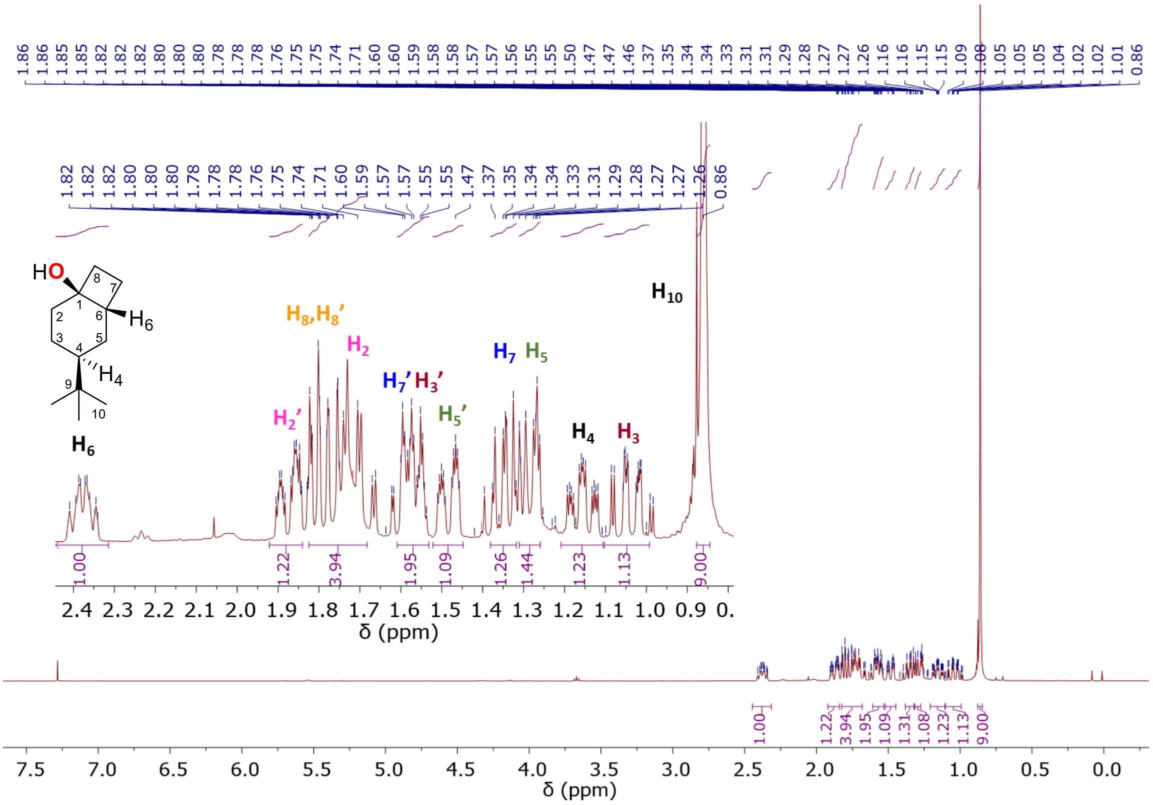

Figure S2. ^{1}H-NMR spectrum (400 MHz, CDCl$_3$) of *cis*-4-*tert*-butylbicyclo[4.2.0]octan-1-ol (**P1b-OH**).

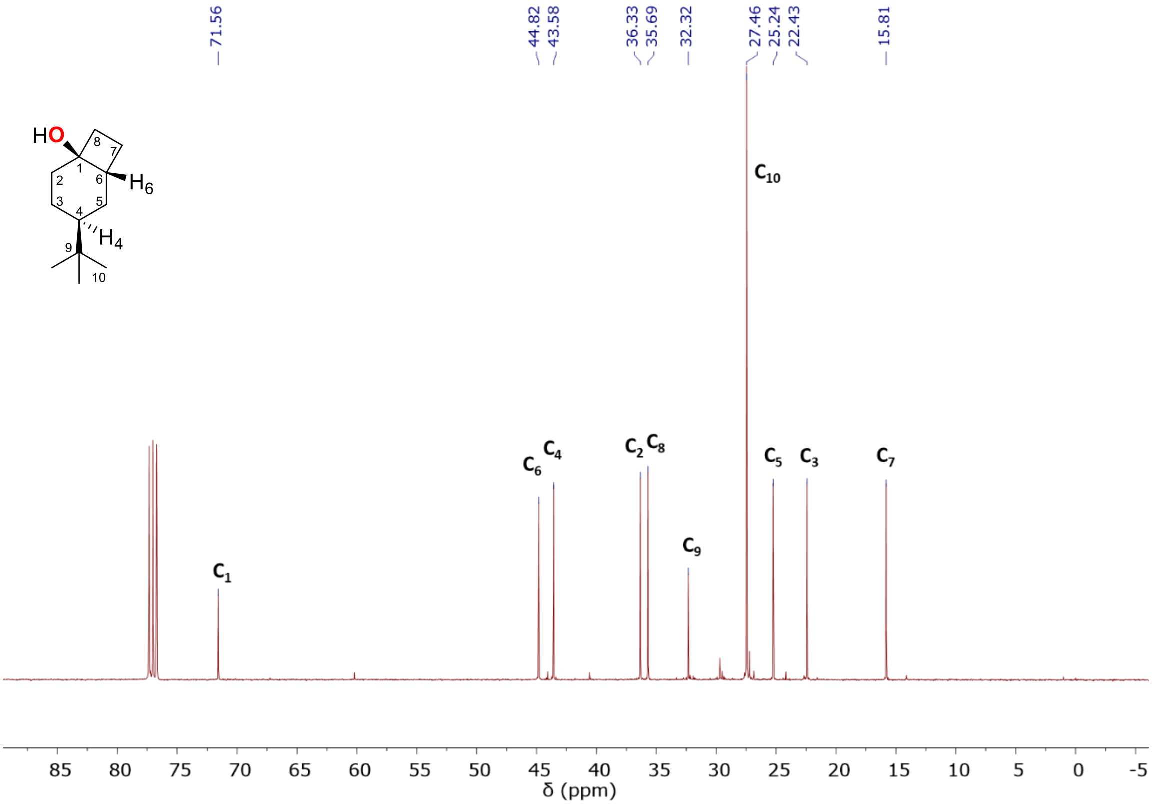

Figure S3. ^{13}C{^{1}H}-NMR spectrum (400 MHz, CDCl$_3$) of *cis*-4-*tert*-butylbicyclo[4.2.0]octan-1-ol (**P1b-OH**).

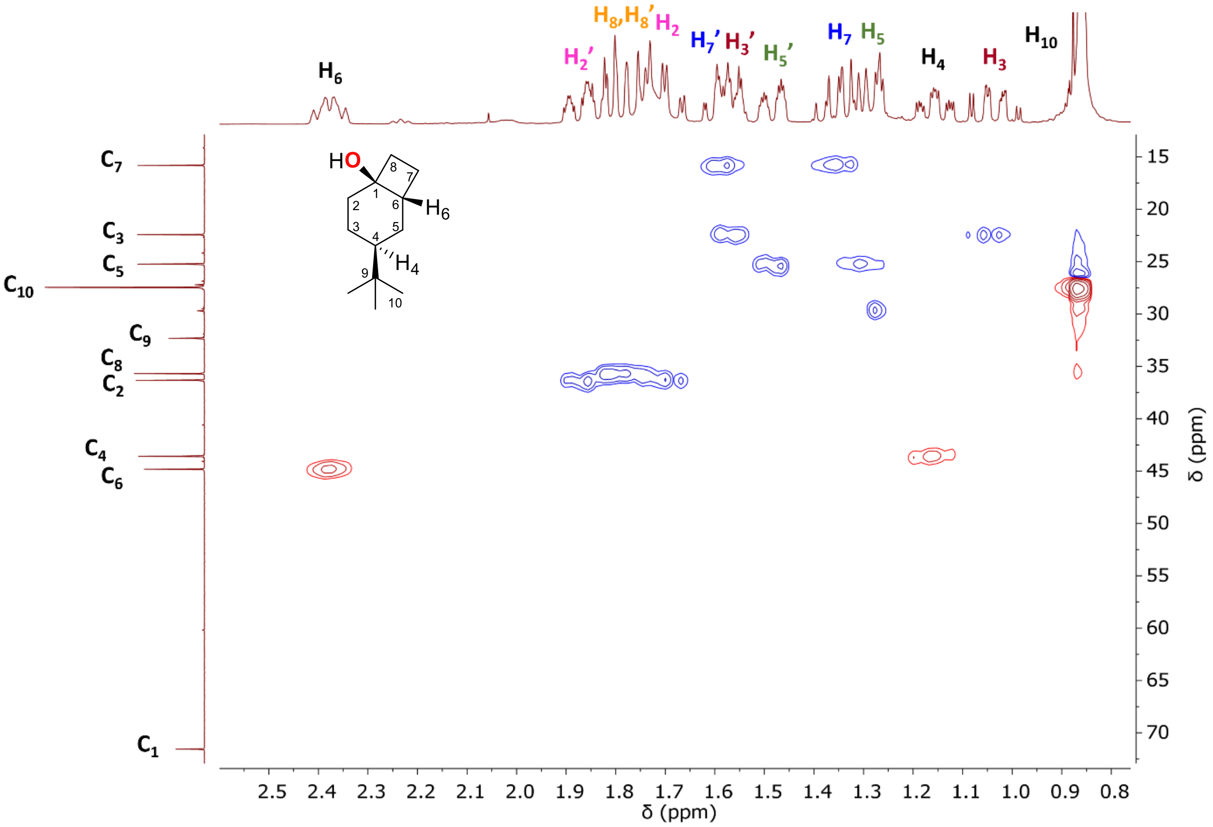

Figure S4. HSQC-NMR spectrum (400 MHz, $CDCl_3$) of *cis*-4-*tert*-butylbicyclo[4.2.0]octan-1-ol (**P1b-OH**).

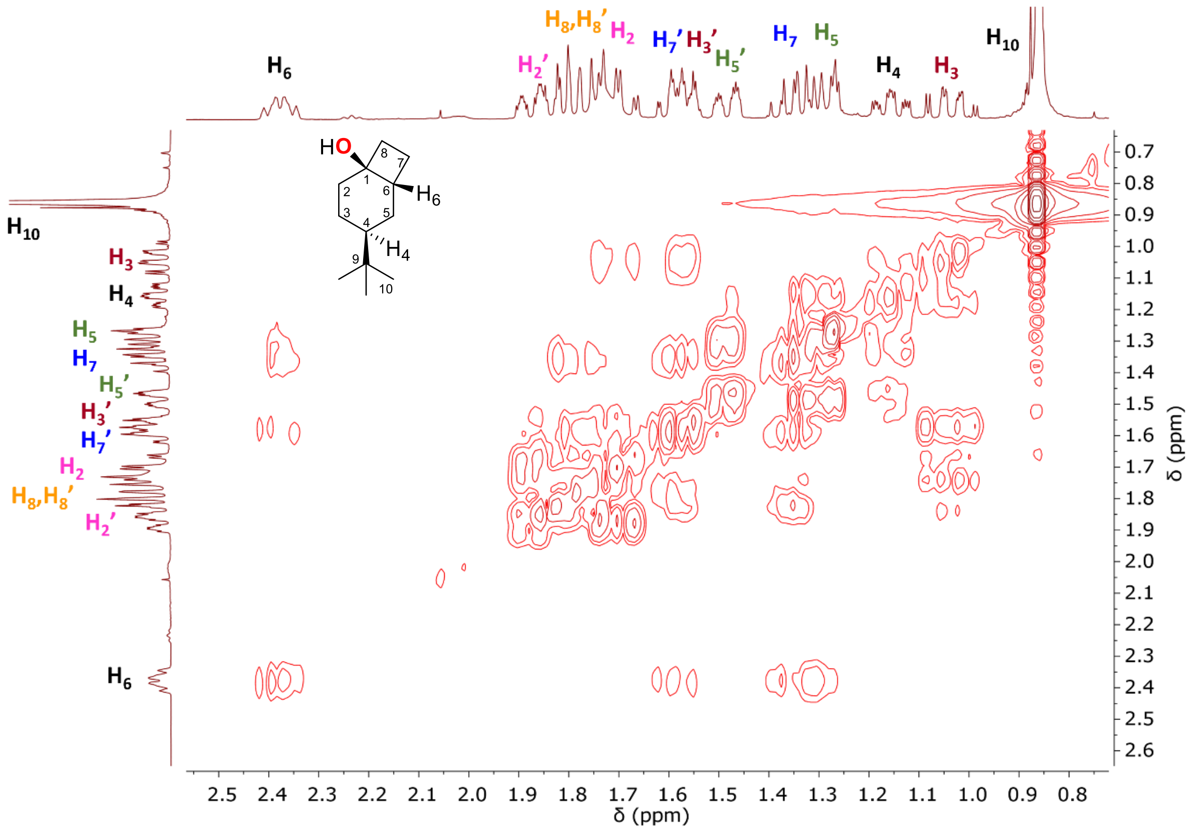

Figure S5. COSY-NMR spectrum (400 MHz, $CDCl_3$) of *cis*-4-*tert*-butylbicyclo[4.2.0]octan-1-ol (**P1b-OH**).

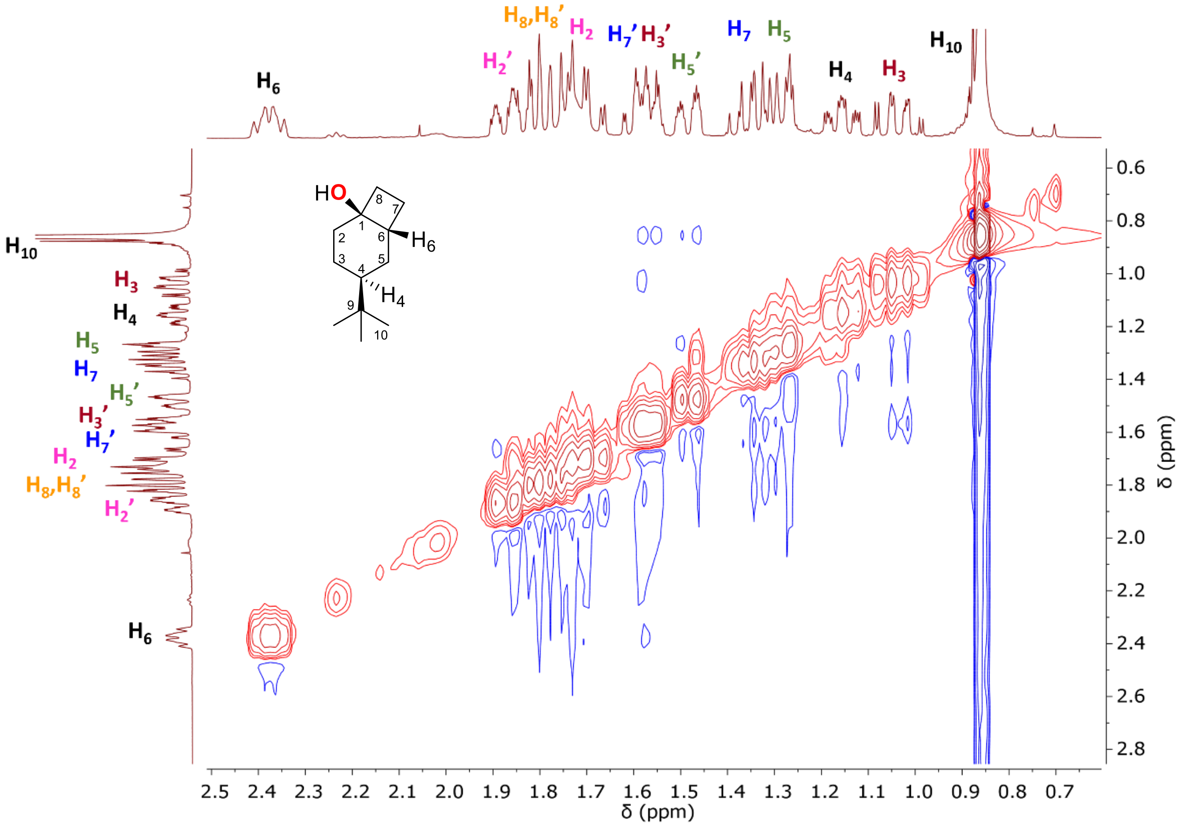

Figure S6. NOESY-NMR spectrum (400 MHz, $CDCl_3$) of *cis*-4-*tert*-butylbicyclo[4.2.0]octan-1-ol (**P1b-OH**).

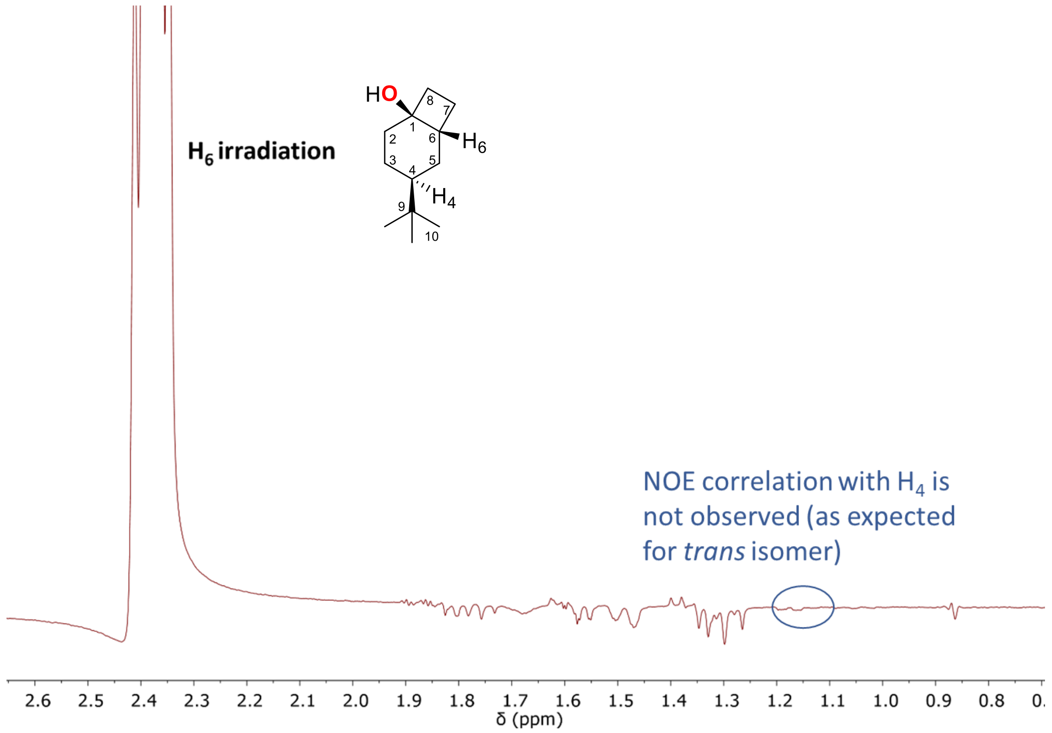

Figure S7. Selective NOE-NMR spectrum (400 MHz, $CDCl_3$, δ_{irr} = 2.38 ppm) of *cis*-4-*tert*-butylbicyclo[4.2.0]octan-1-ol (**P1b-OH**).

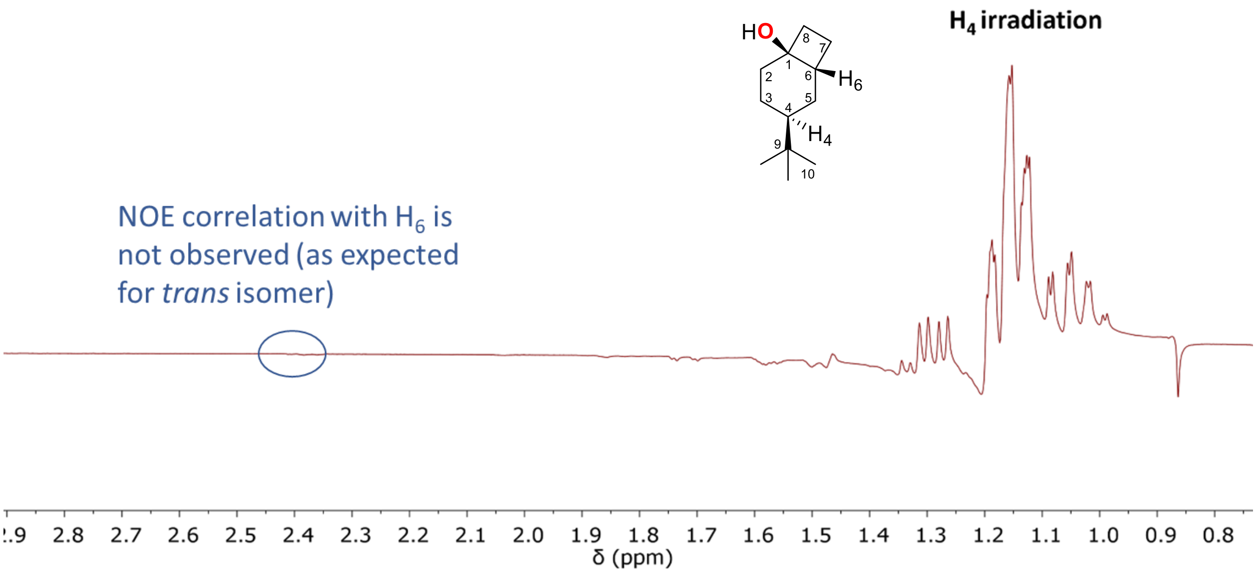

Figure S8. Selective NOE-NMR spectrum (400 MHz, $CDCl_3$, δ_{irr} = 1.16 ppm) of *cis*-4-*tert*-butylbicyclo[4.2.0]octan-1-ol (**P1b-OH**).

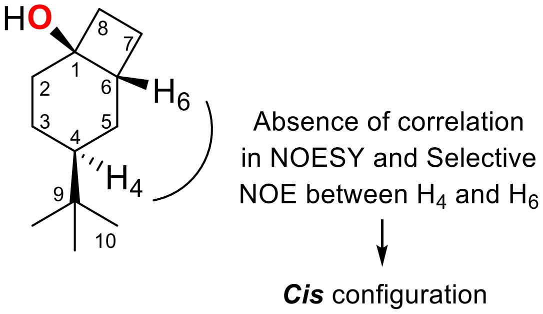

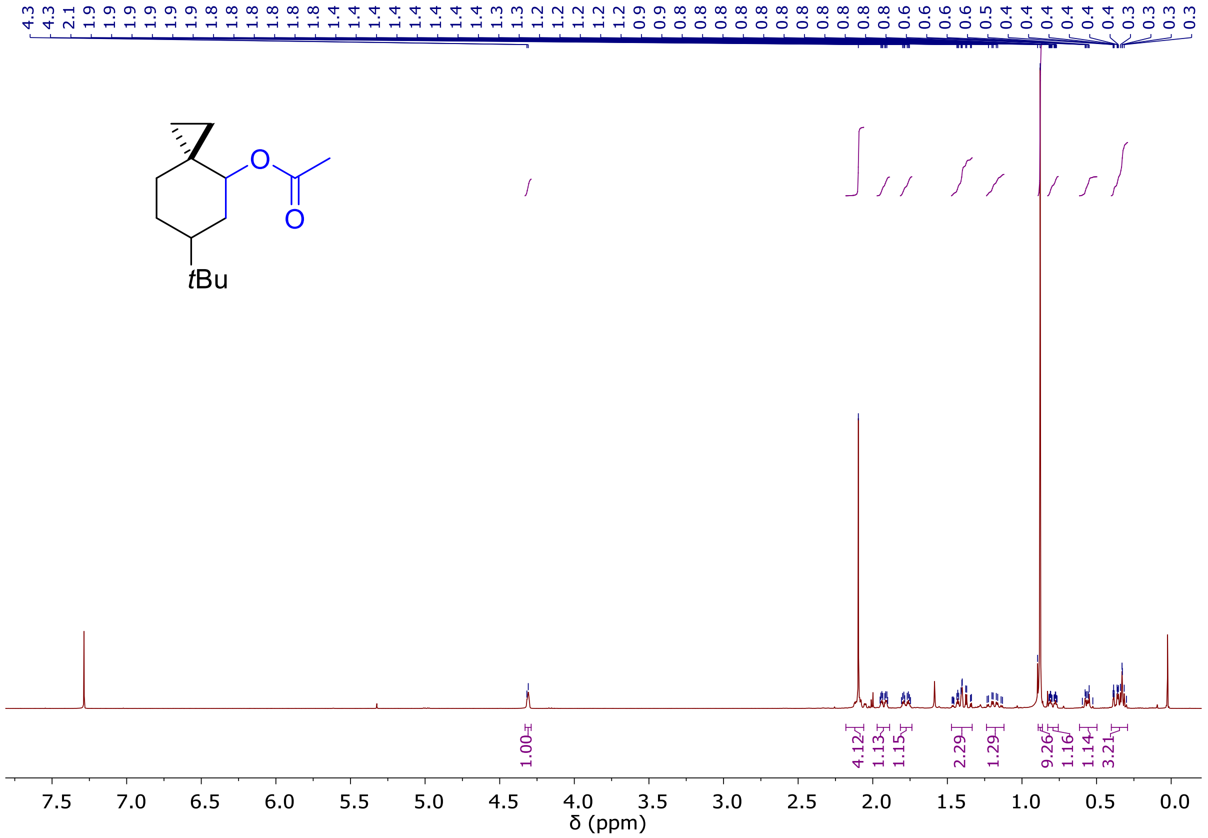

Figure S9. ^{1}H-NMR spectrum (400 MHz, $CDCl_3$) of *trans*-6-*tert*-butylspiro[2.5]octan-4-yl acetate (**P1a-OX$_1$**).

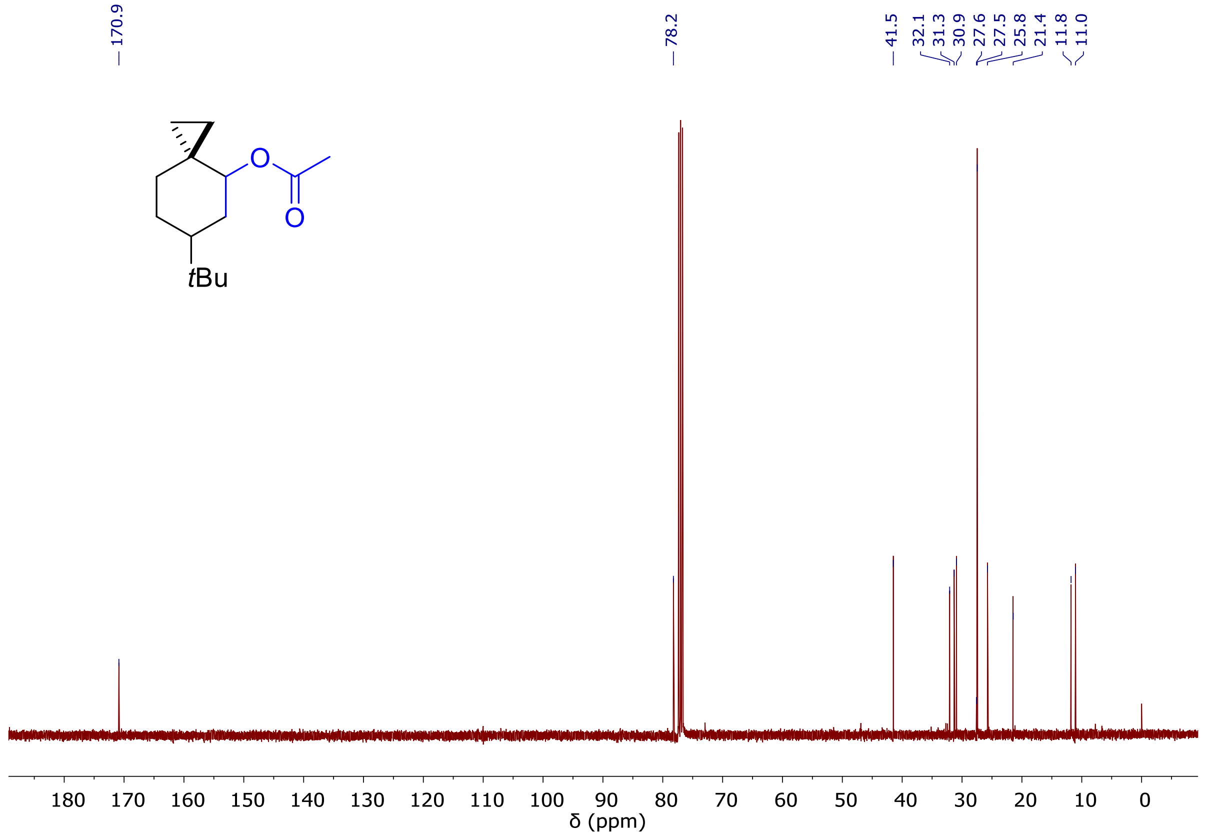

Figure S10. ^{13}C{^{1}H}-NMR spectrum (400 MHz, $CDCl_3$) of *trans*-6-*tert*-butylspiro[2.5]octan-4-yl acetate (**P1a-OX$_1$**).

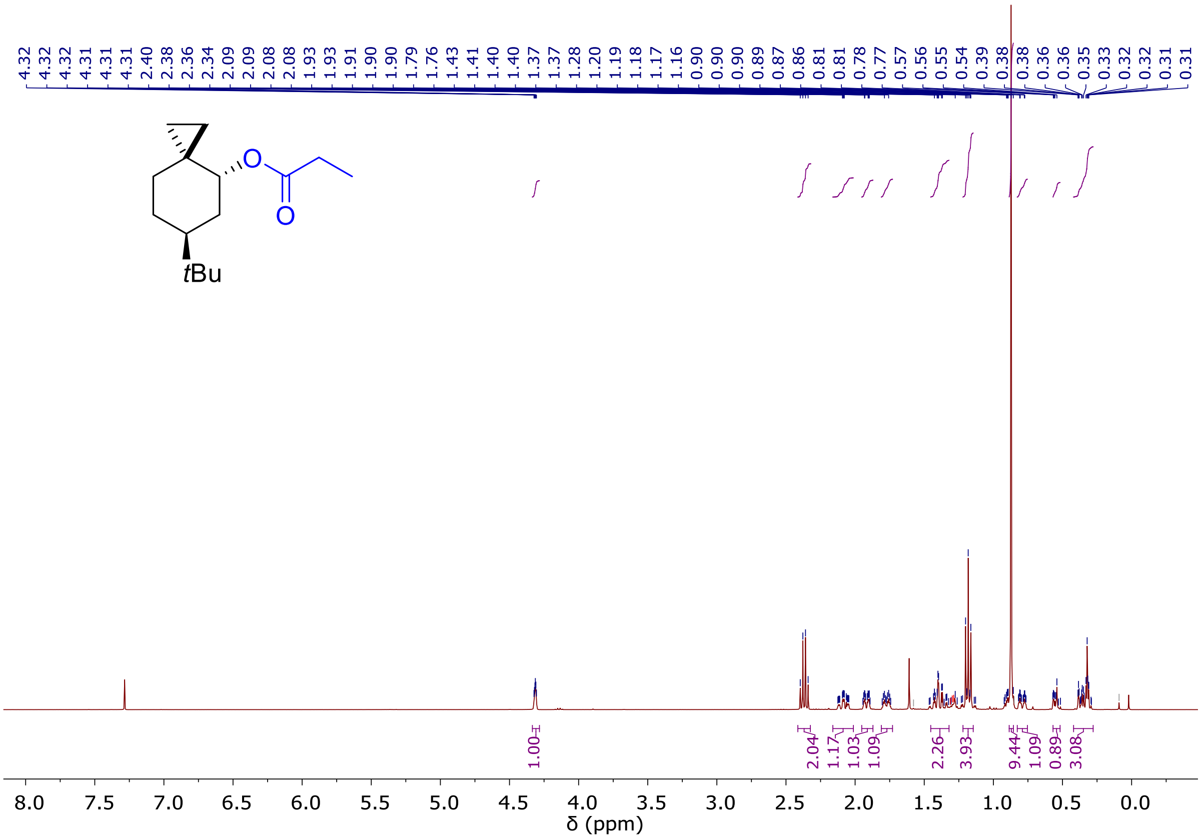

Figure S11. ^{1}H-NMR spectrum (400 MHz, $CDCl_3$) of *trans*-6-*tert*-butylspiro[2.5]octan-4-yl propionate (**P1a-OX$_2$**).

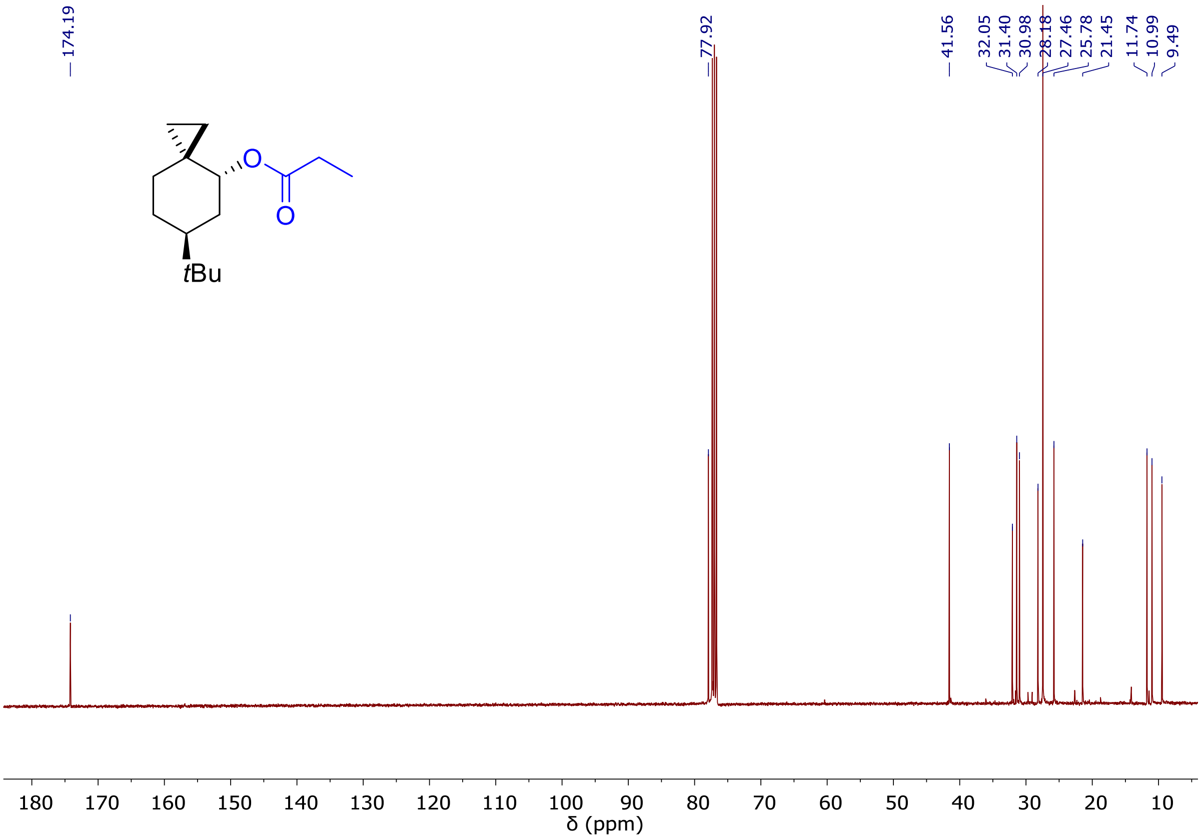

Figure S12. ^{13}C{^{1}H}-NMR spectrum (400 MHz, $CDCl_3$) of *trans*-6-*tert*-butylspiro[2.5]octan-4-yl propionate (**P1a-OX$_2$**).

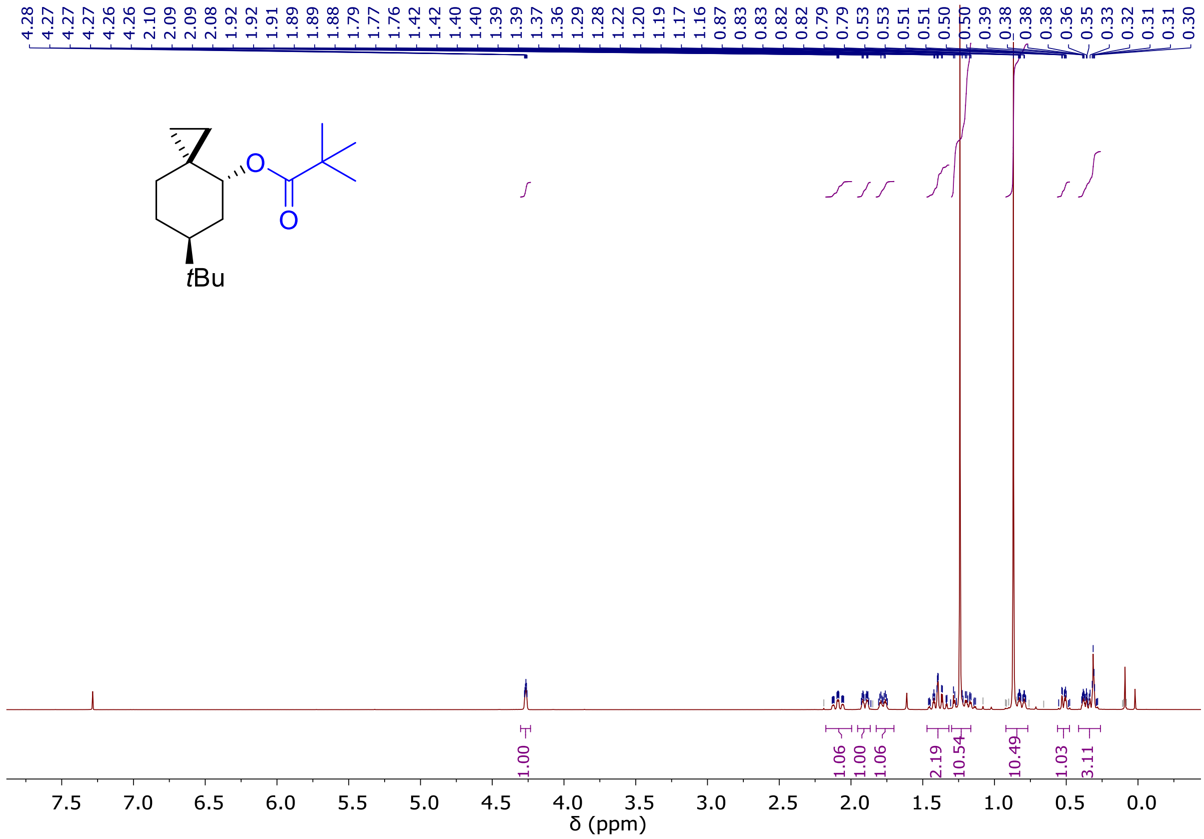

Figure S13. ^{1}H-NMR spectrum (400 MHz, $CDCl_3$) of *trans*-6-*tert*-butylspiro[2.5]octan-4-yl pivalate (**P1a-OX**$_3$).

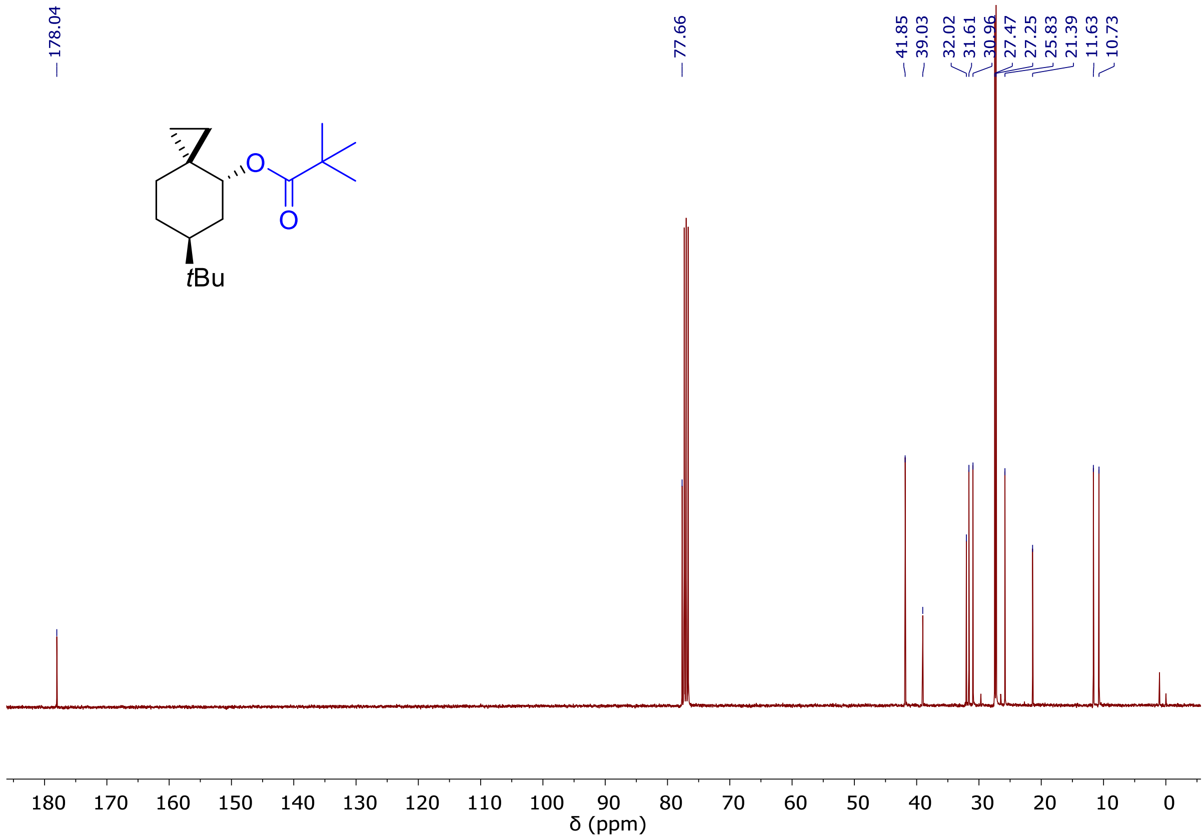

Figure S14. ^{13}C{^{1}H}-NMR spectrum (400 MHz, $CDCl_3$) of *trans*-6-*tert*-butylspiro[2.5]octan-4-yl pivalate (**P1a-OX**$_3$).

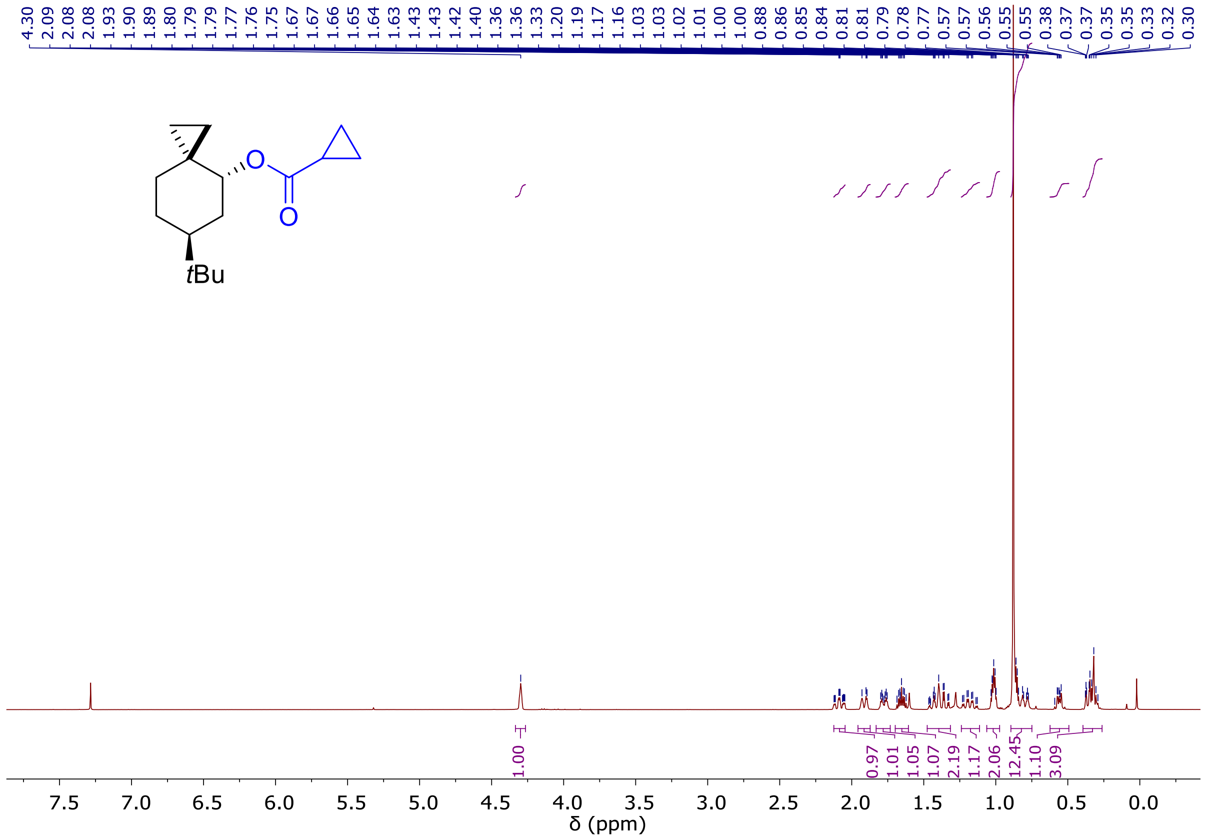

Figure S15. ^{1}H-NMR spectrum (400 MHz, $CDCl_3$) of *trans*-6-*tert*-butylspiro[2.5]octan-4-yl cyclopropanecarboxylate (**P1a-OX$_4$**).

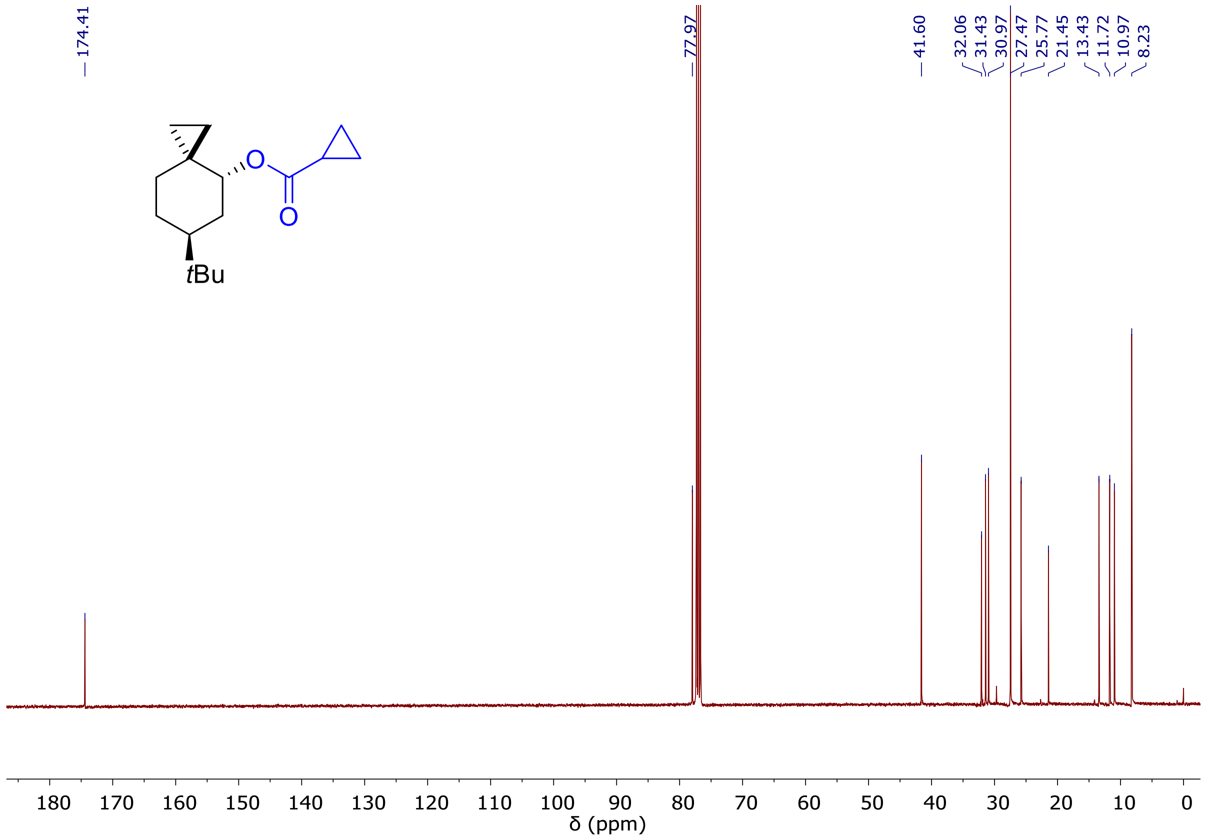

Figure S16. ^{13}C{^{1}H}-NMR spectrum (400 MHz, $CDCl_3$) of *trans*-6-*tert*-butylspiro[2.5]octan-4-yl cyclopropanecarboxylate (**P1a-OX$_4$**).

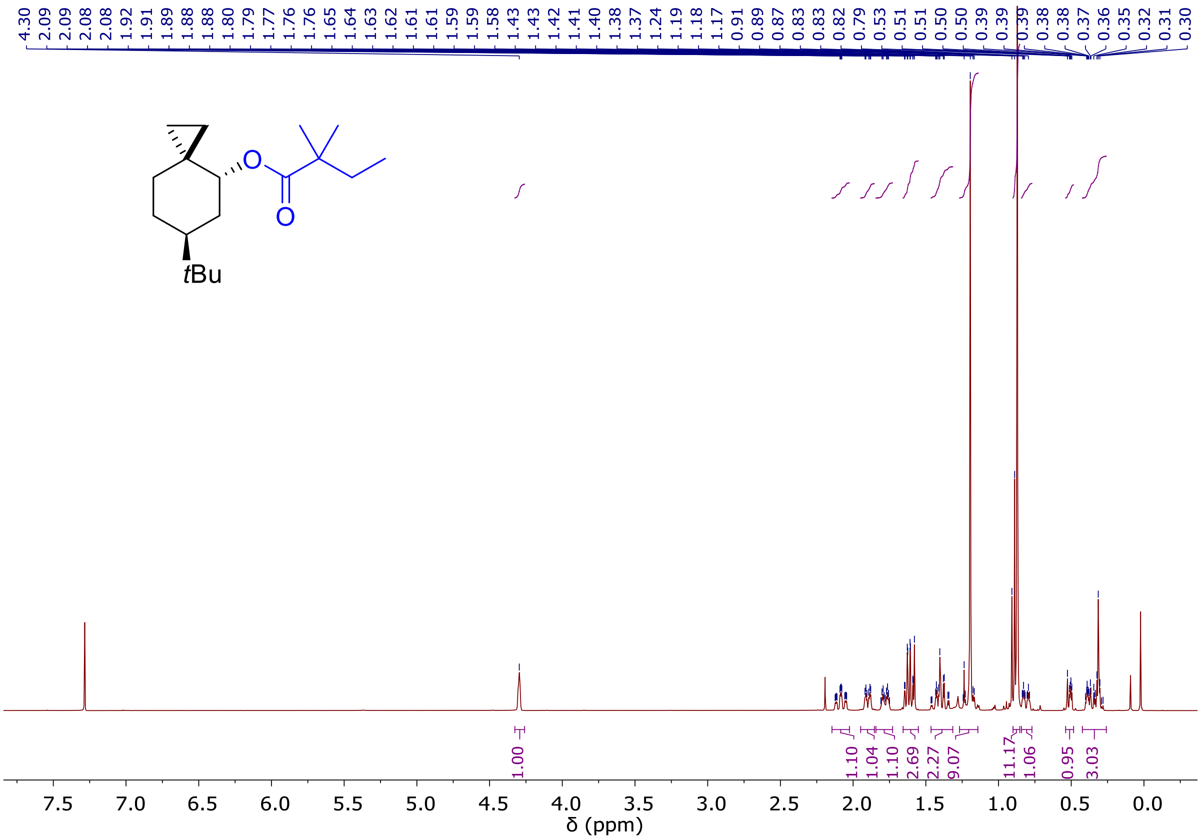

Figure S17. ^{1}H-NMR spectrum (400 MHz, $CDCl_3$) of *trans*-6-*tert*-butylspiro[2.5]octan-4-yl 2,2-dimethylbutanoate (**P1a-OX$_5$**).

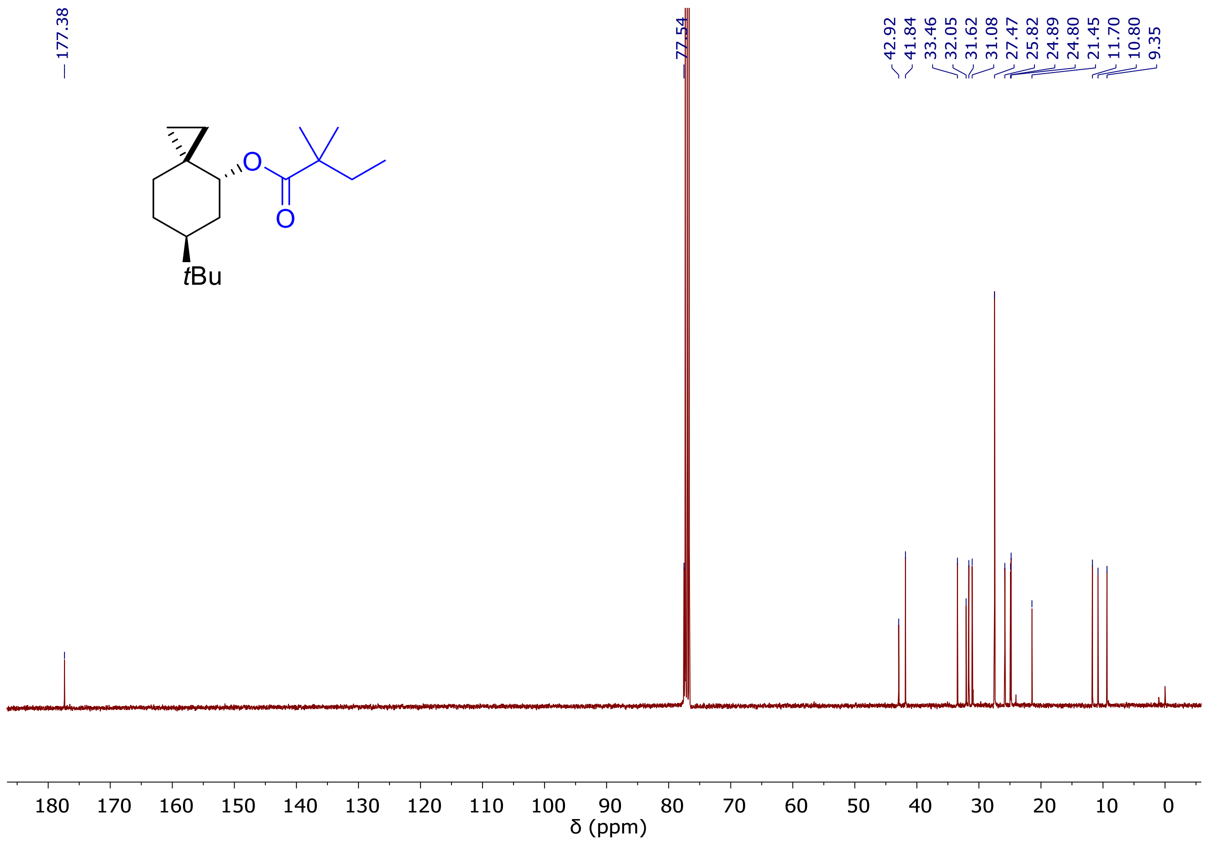

Figure S18. ^{13}C{^{1}H}-NMR spectrum (400 MHz, $CDCl_3$) of *trans*-6-*tert*-butylspiro[2.5]octan-4-yl 2,2-dimethylbutanoate (**P1a-OX$_5$**).

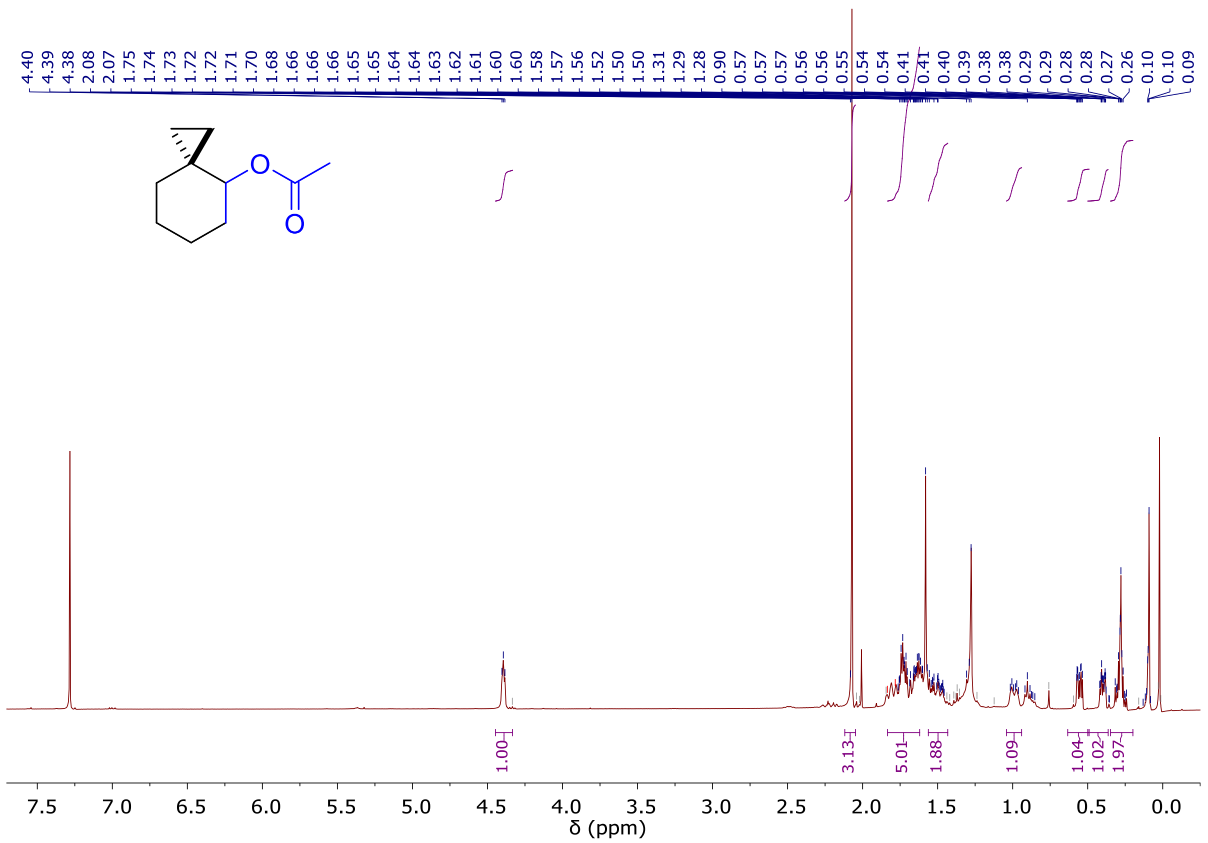

Figure S19. ^{1}H-NMR spectrum (400 MHz, $CDCl_3$) of spiro[2.5]octan-4-yl acetate (**P2a-OX$_1$**).

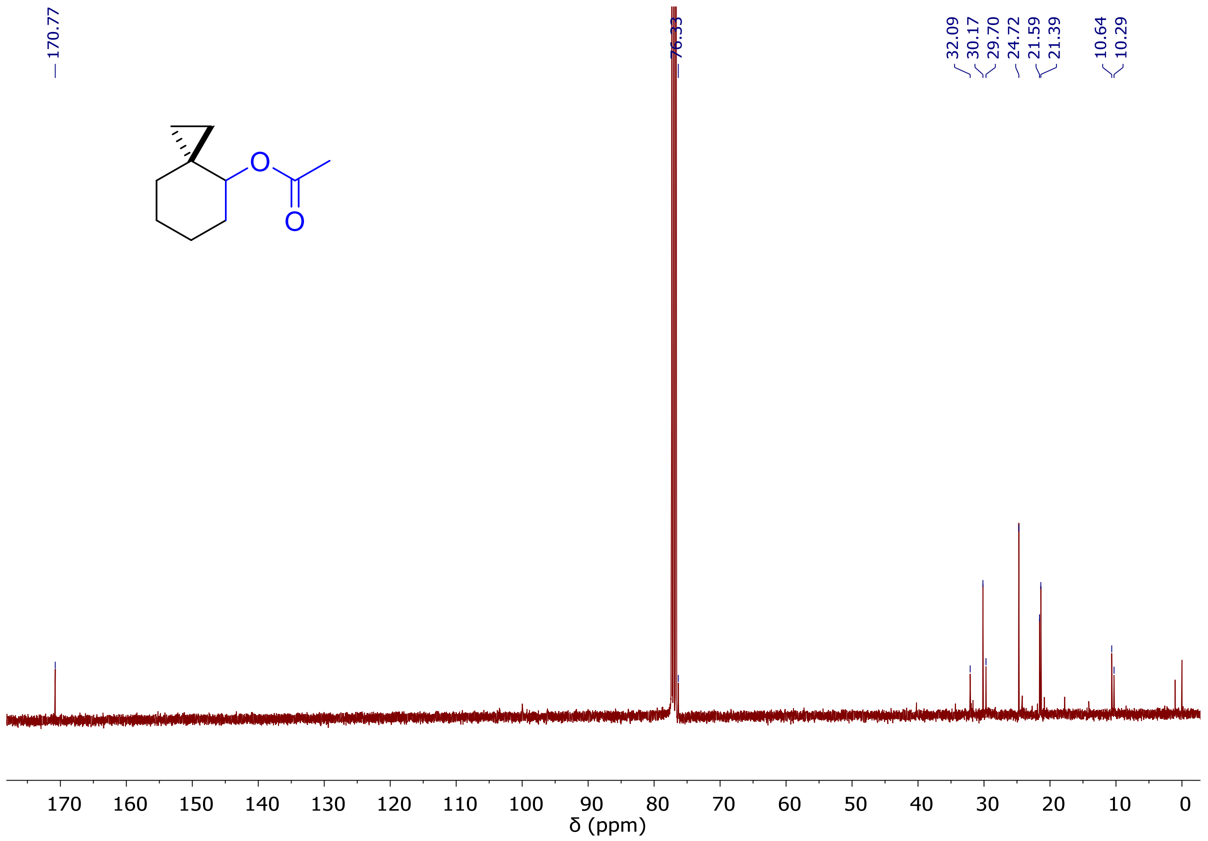

Figure S20. ^{13}C{^{1}H}-NMR spectrum (400 MHz, $CDCl_3$) of spiro[2.5]octan-4-yl acetate (**P2a-OX$_1$**).

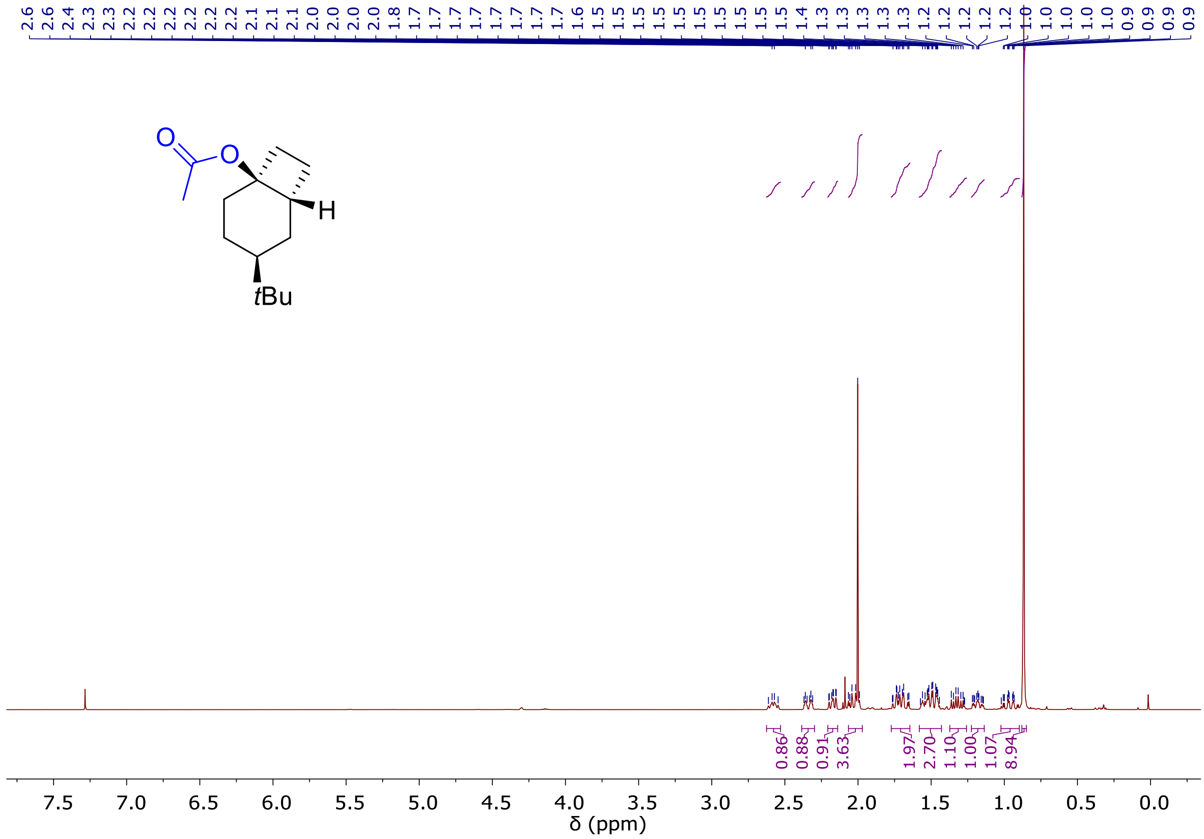

Figure S21. ^{1}H-NMR spectrum (400 MHz, $CDCl_3$) of *cis*-4-*tert*-butylbicyclo[4.2.0]octan-1-yl acetate **(P1b-OX$_1$)**.

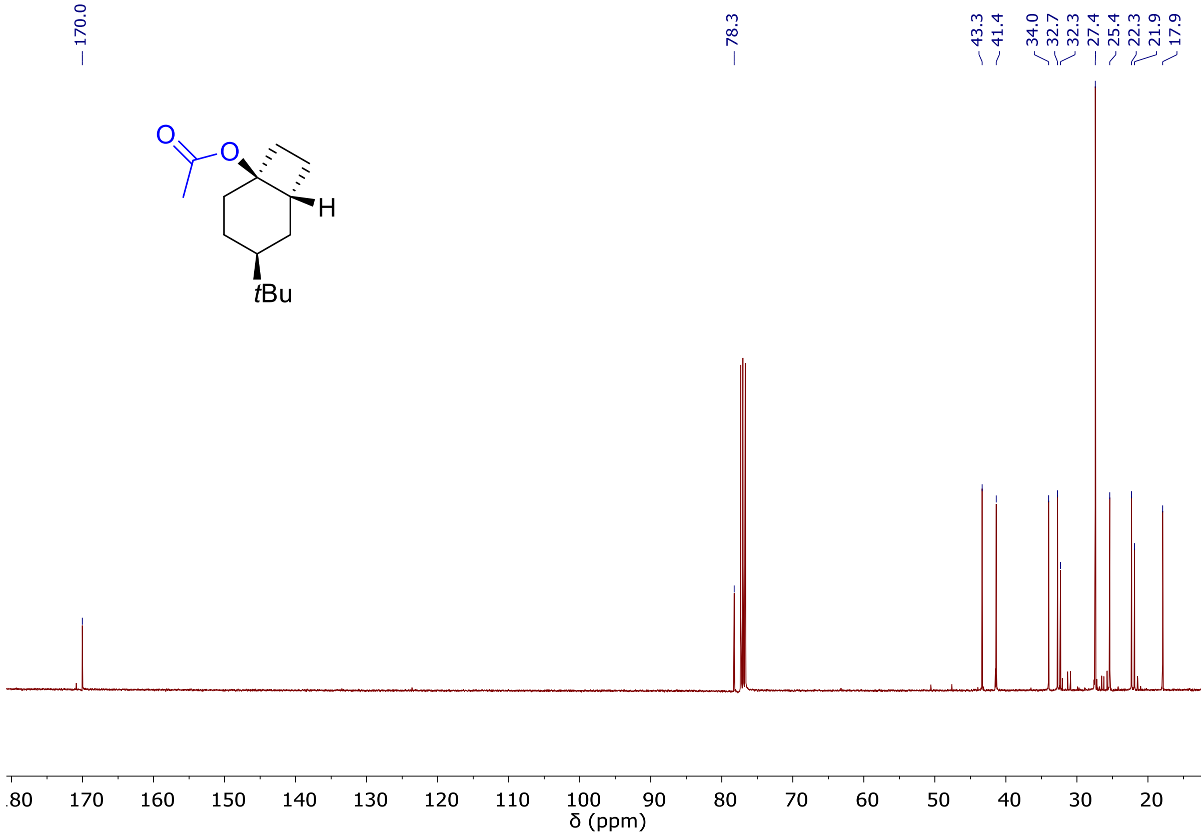

Figure S22. ^{13}C{^{1}H}-NMR spectrum (400 MHz, $CDCl_3$) of *cis*-4-*tert*-butylbicyclo[4.2.0]octan-1-yl acetate **(P1b-OX$_1$)**.

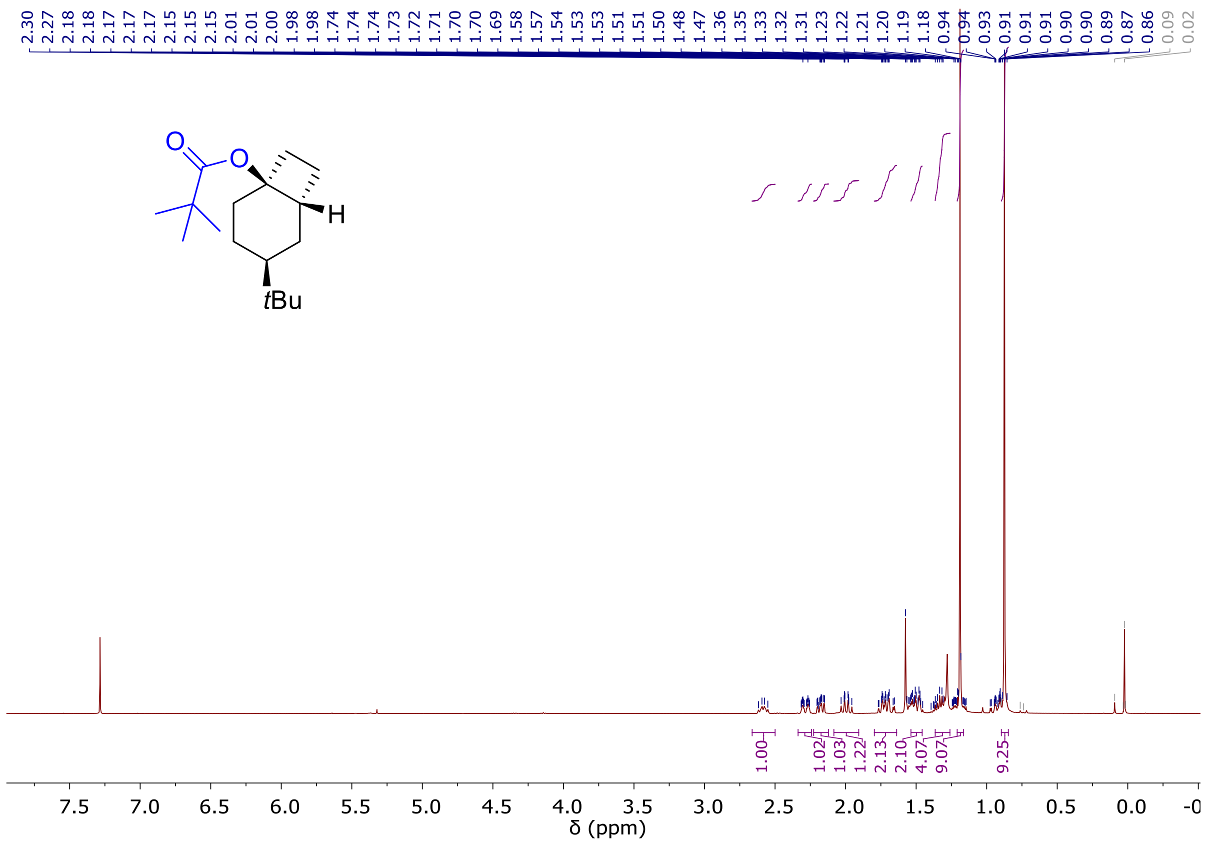

Figure S23. ^{1}H-NMR spectrum (400 MHz, $CDCl_3$) of *cis*-4-*tert*-butylbicyclo[4.2.0]octan-1-yl pivalate **(P1b-OX$_3$)**.

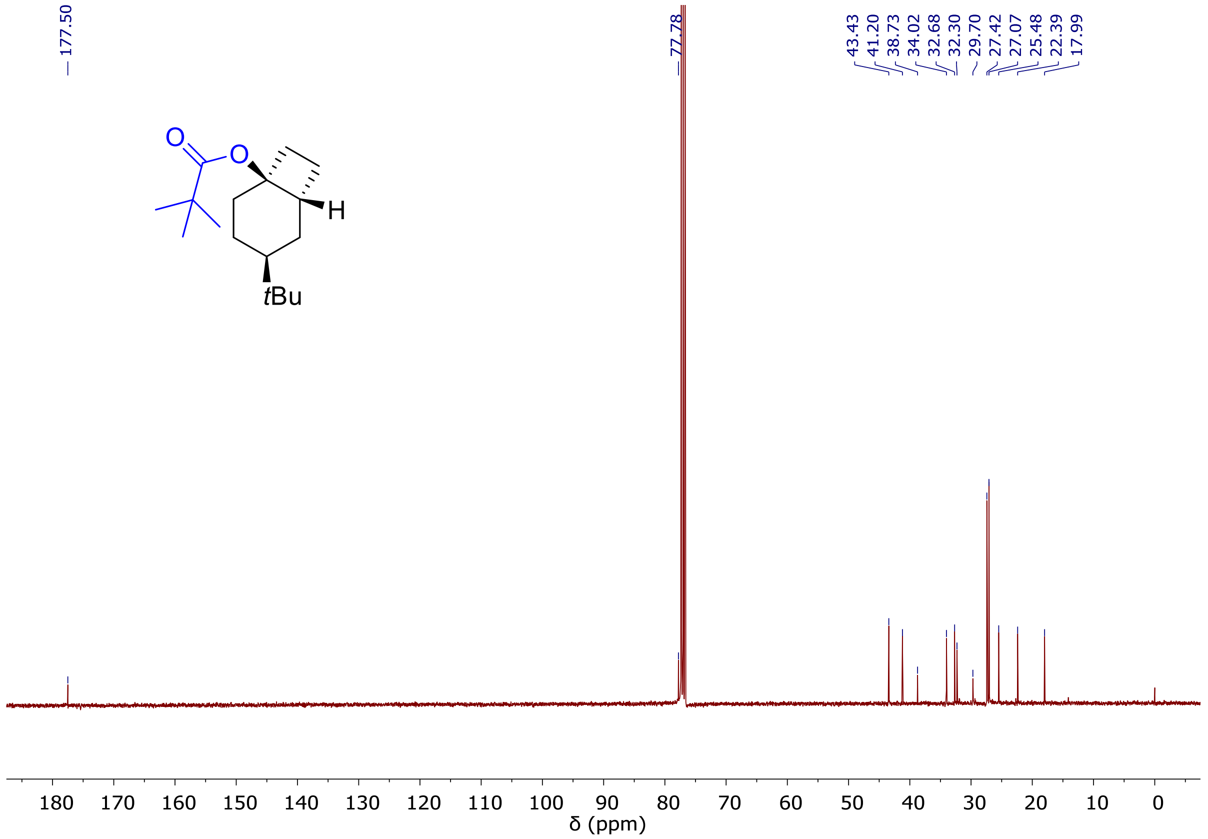

Figure S24. ^{13}C{^{1}H}-NMR spectrum (400 MHz, $CDCl_3$) of *cis*-4-*tert*-butylbicyclo[4.2.0]octan-1-yl pivalate **(P1b-OX$_3$)**.

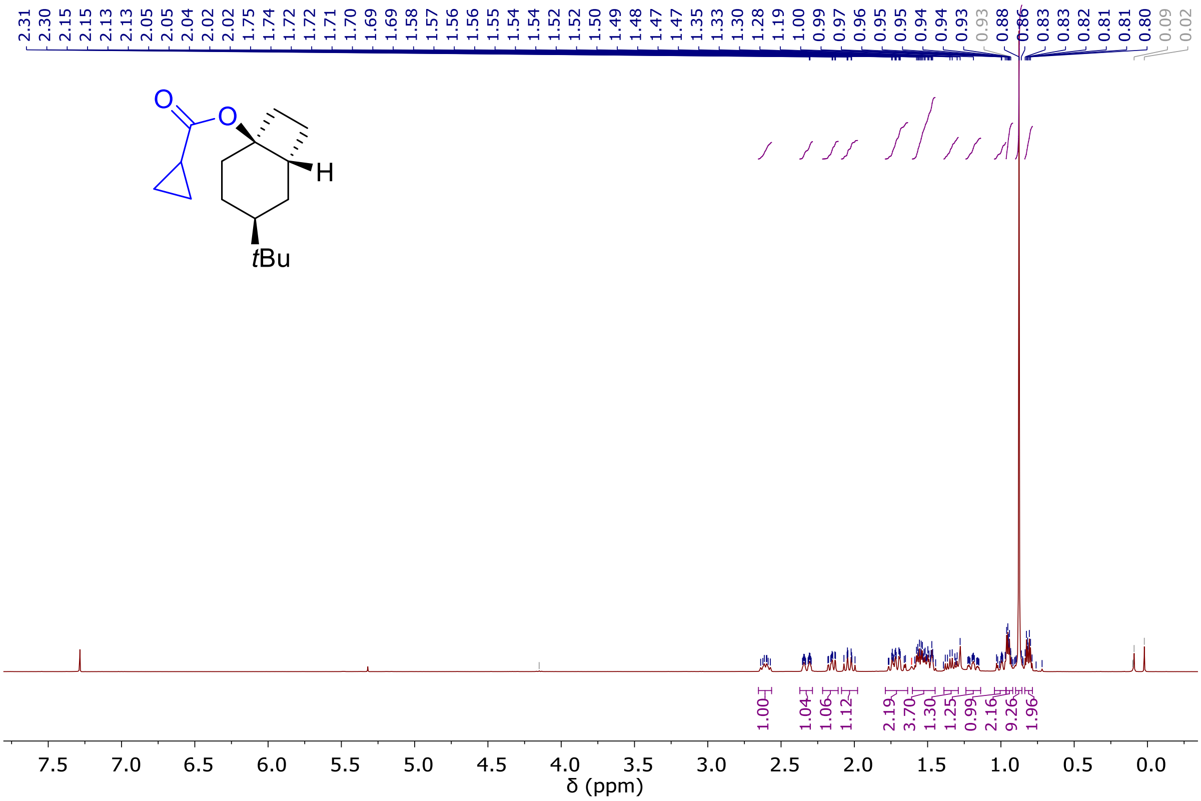

Figure S25. ^{1}H-NMR spectrum (400 MHz, $CDCl_3$) of *cis*-4-*tert*-butylbicyclo[4.2.0]octan-1-yl cyclopropanecarboxylate **(P1b-OX4)**.

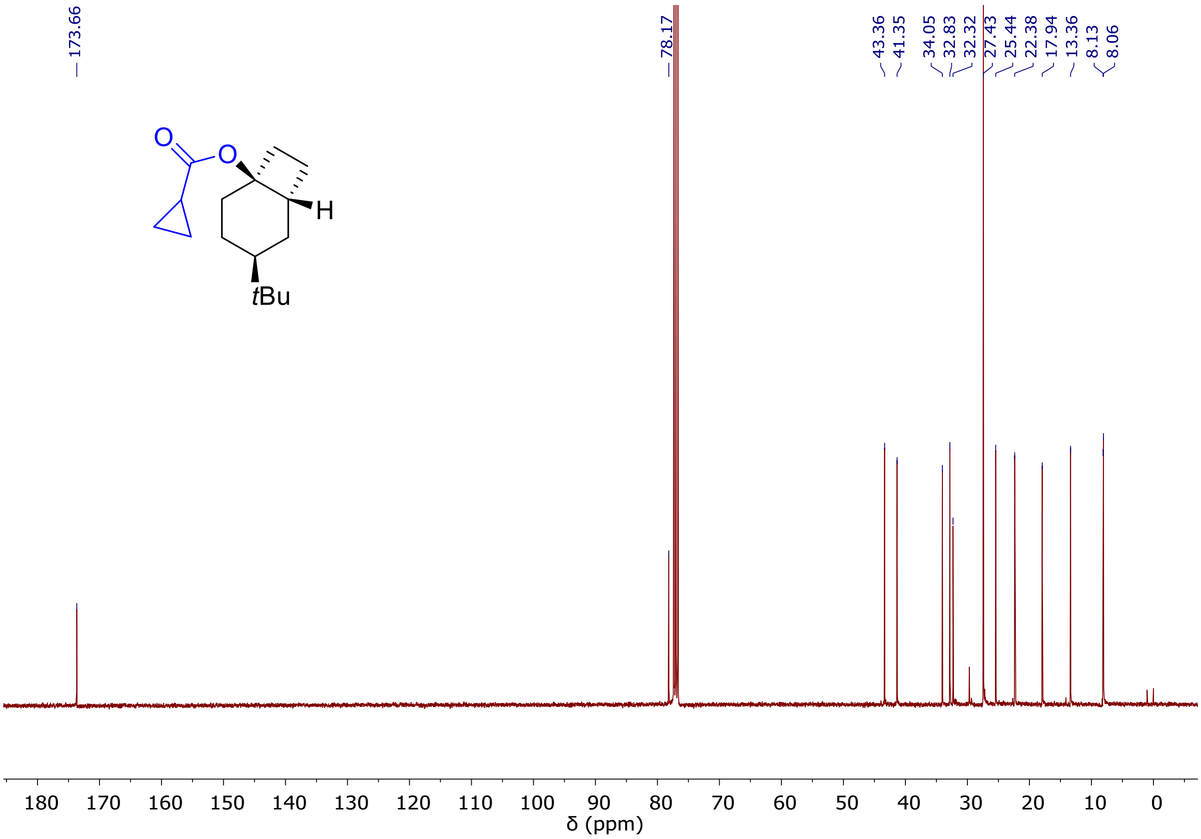

Figure S26. ^{13}C{^{1}H}-NMR spectrum (400 MHz, $CDCl_3$) of *cis*-4-*tert*-butylbicyclo[4.2.0]octan-1-yl cyclopropanecarboxylate **(P1b-OX4)**.

7. GC chromatograms of chiral products

The chromatograms of the racemic products obtained in the oxidation of **S1** catalyzed by a racemic catalyst are reported in **Figures S27-S30**. The representative chromatograms of the chiral products obtained in the oxidation of **S1** catalyzed by manganese complexes (**Table S13**) are shown in **Figures S31-S58.**

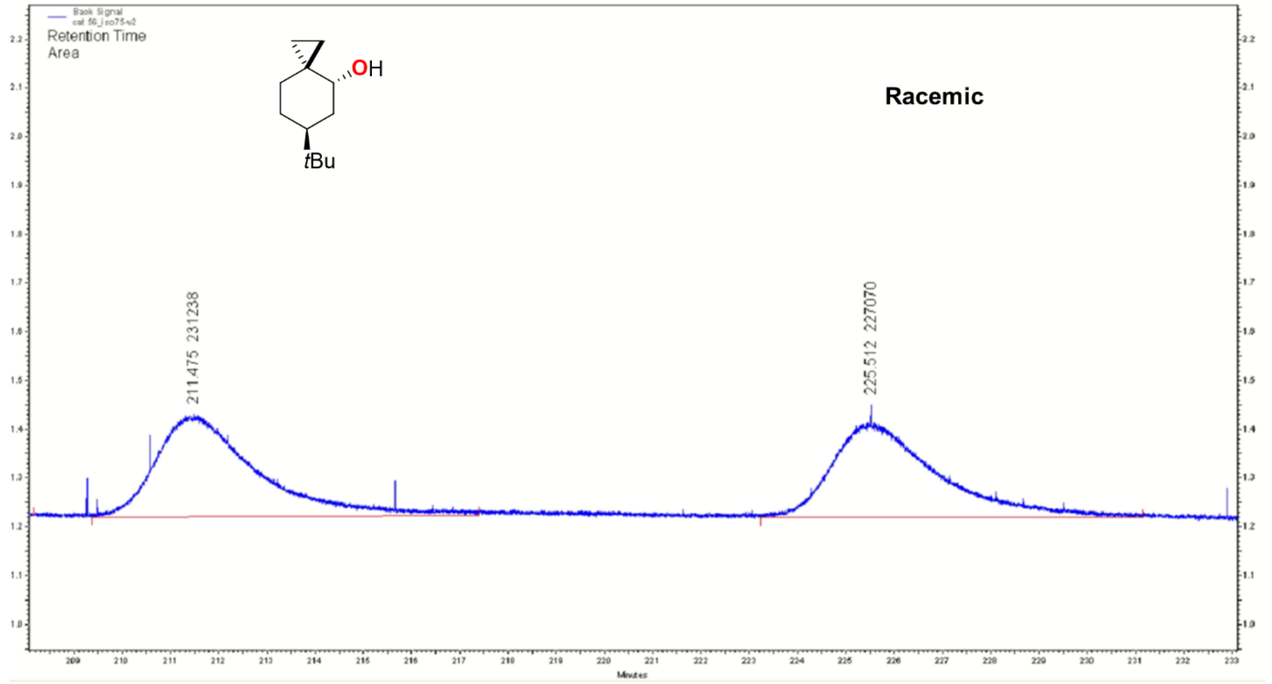

Figure S27: Chromatogram of racemic **P1a-OH**.

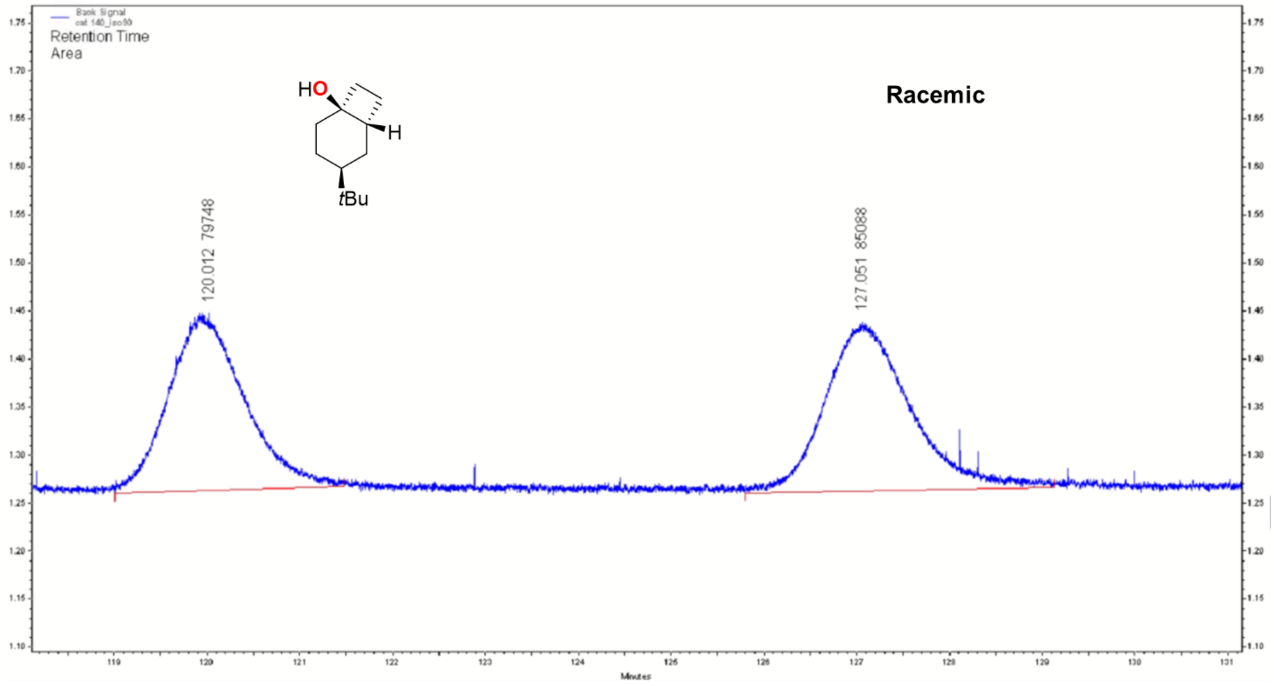

Figure S28: Chromatogram of racemic **P1b-OH**.

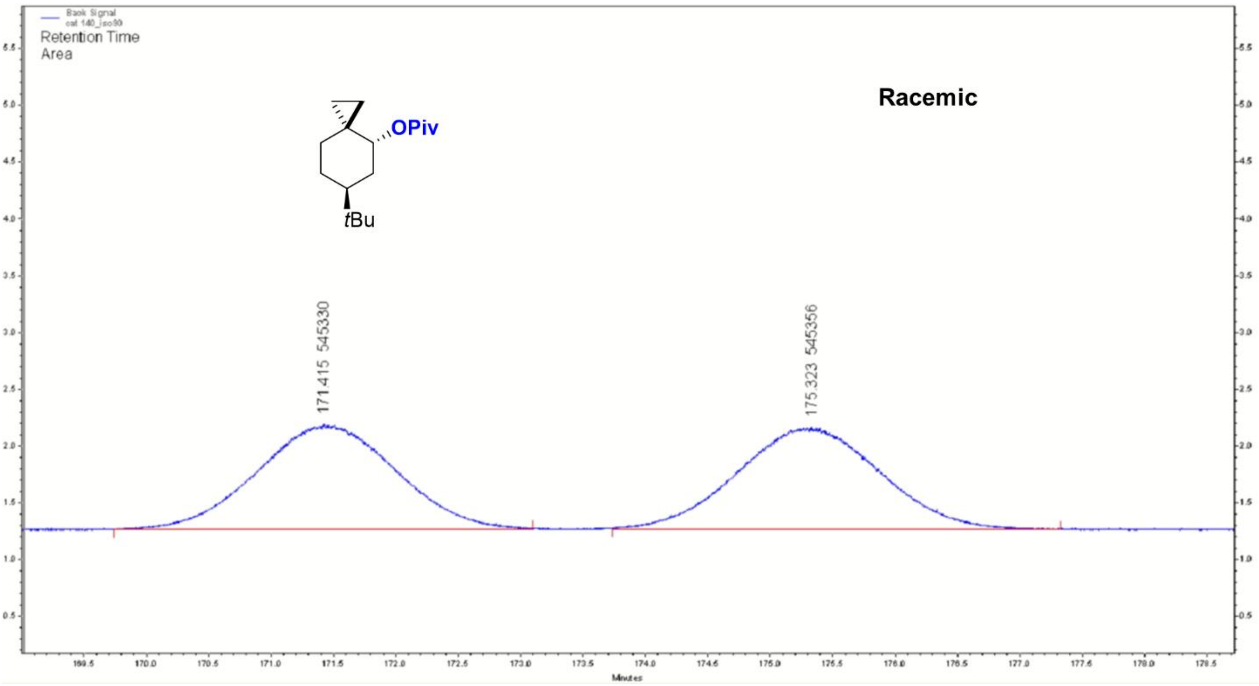

Figure S29: Chromatogram of racemic **P1a-OX3**.

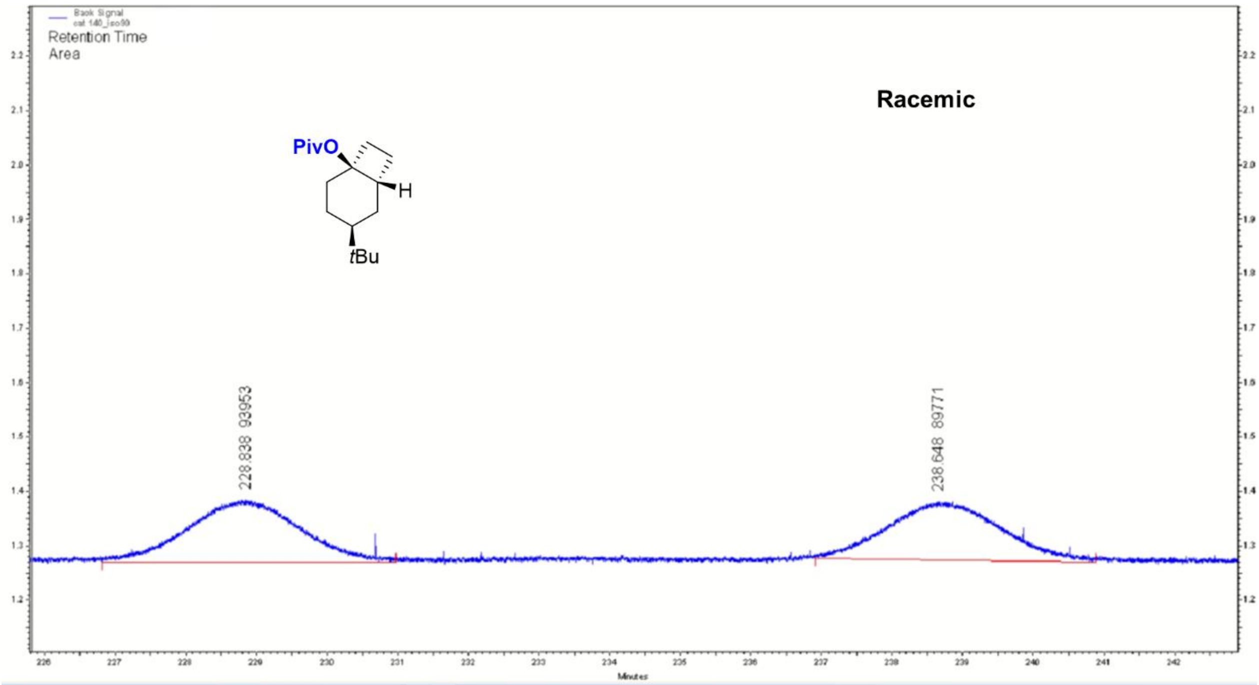

Figure S30: Chromatogram of racemic **P1b-OX3**.

- Oxidation of **S1** catalyzed by [(*R*,*R*)-Mn(OTf)$_2$(mcp)] (entry 1, **Table S13**)

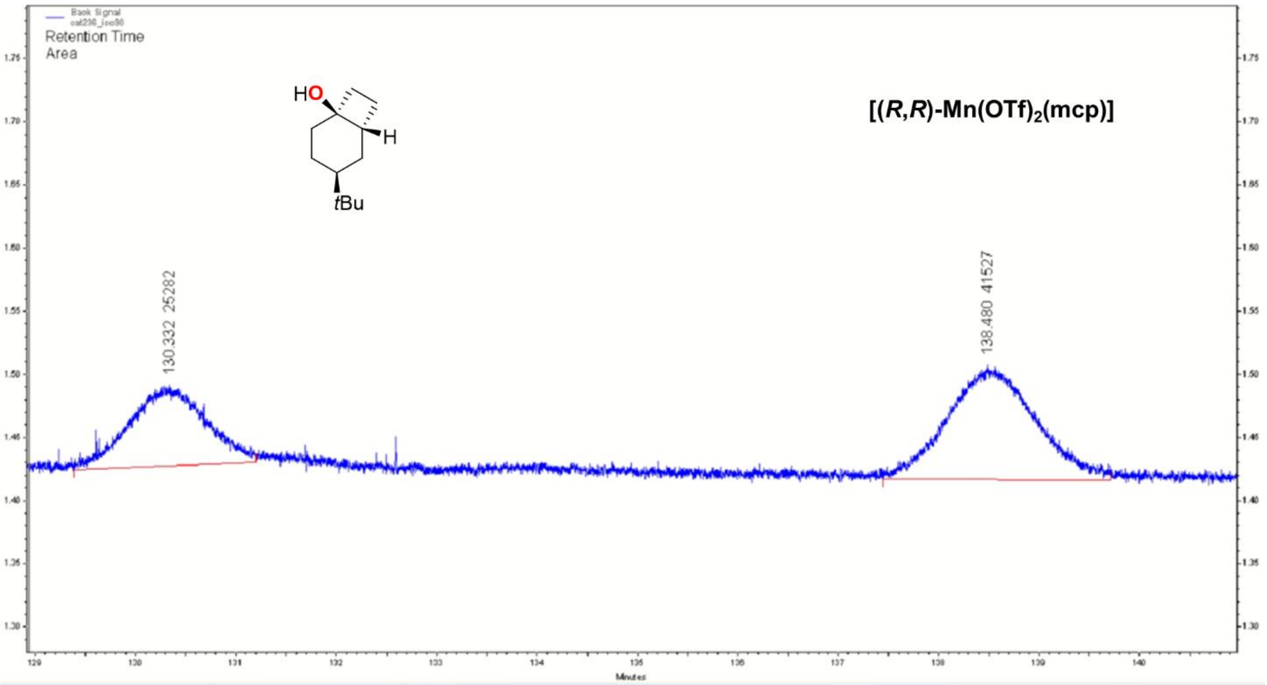

Figure S31: Chromatogram of chiral **P1b-OH**.

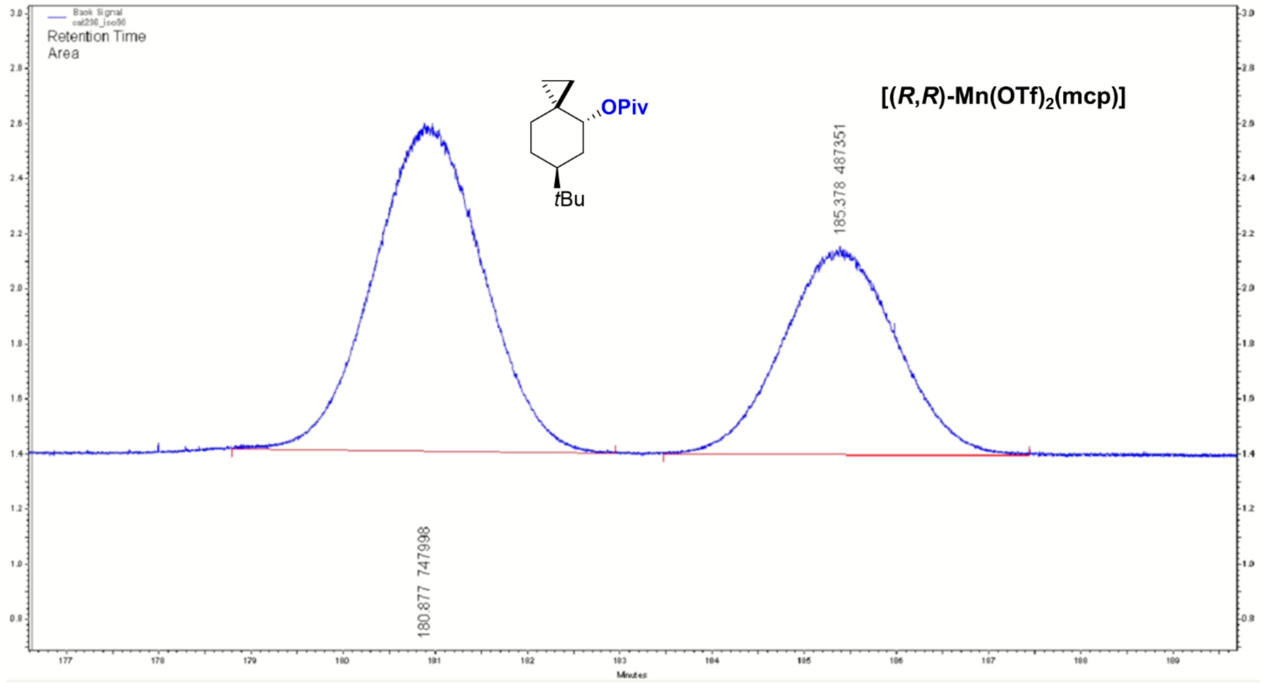

Figure S32: Chromatogram of chiral **P1a-OX$_3$**.

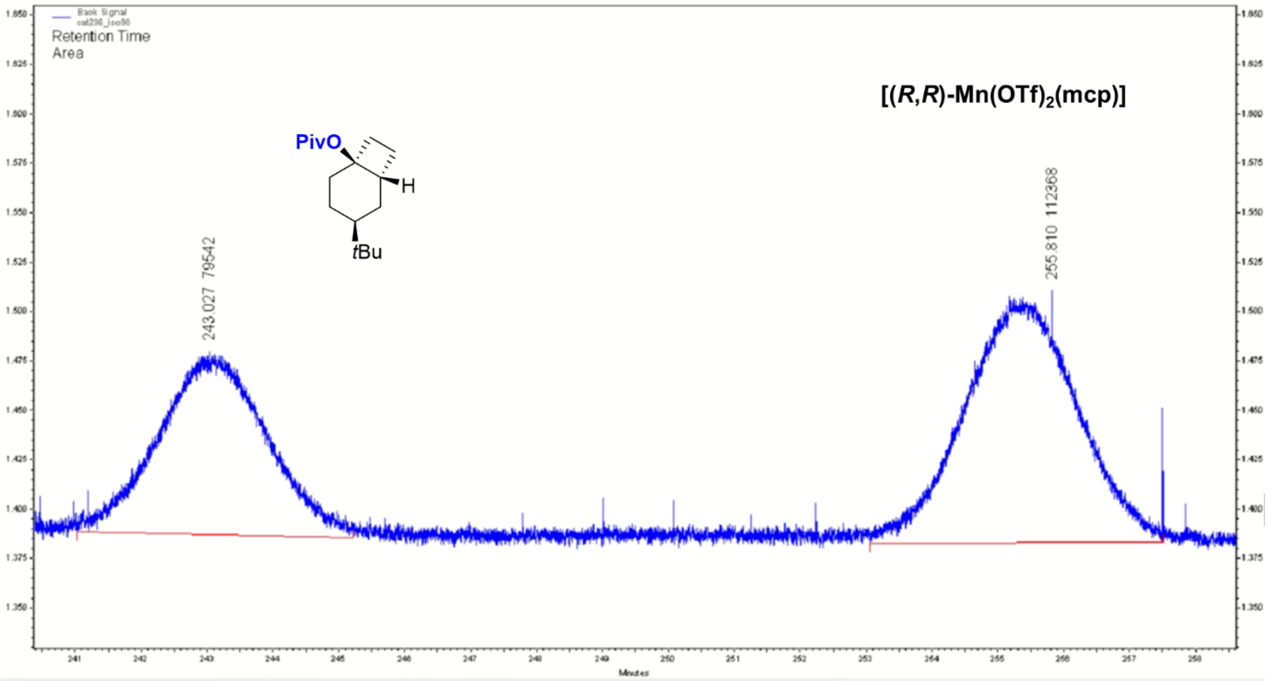

Figure S33: Chromatogram of chiral **P1b-OX**3.

- Oxidation of **S1** catalyzed by [(*S*,*S*)-Mn(OTf)$_2$(^{TIPS}mcp)] (entry 2, **Table S13**)

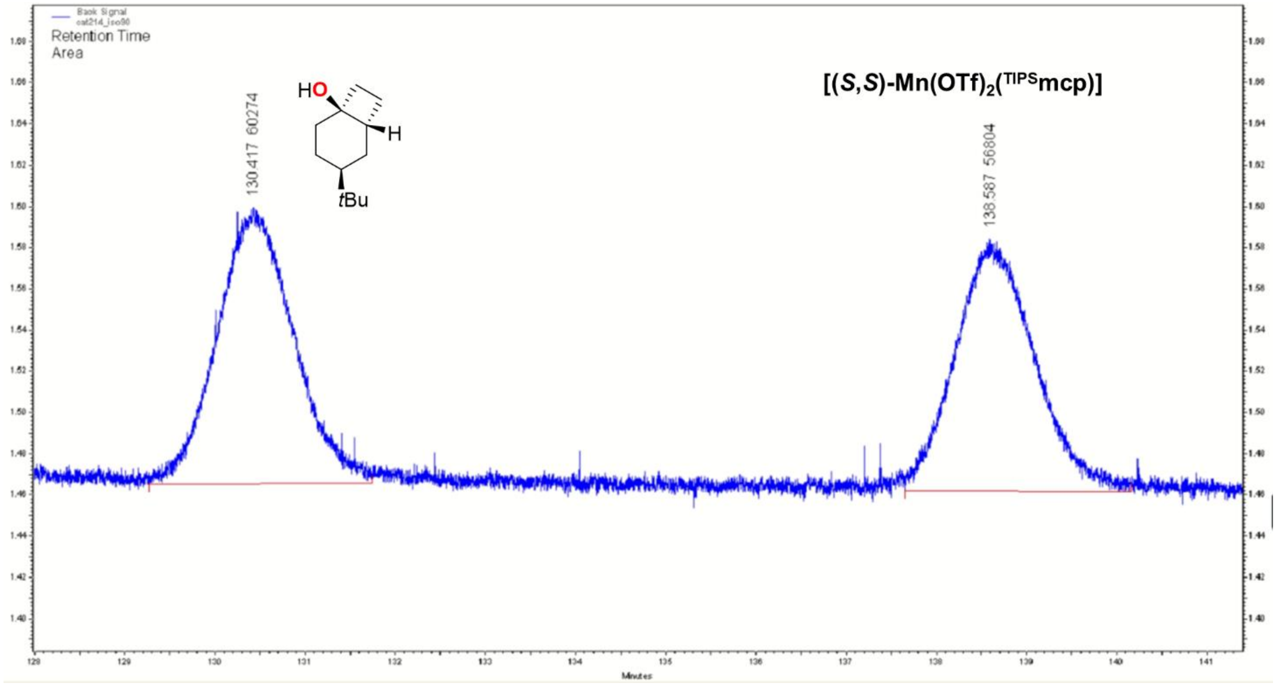

Figure S34: Chromatogram of chiral **P1b-OH**.

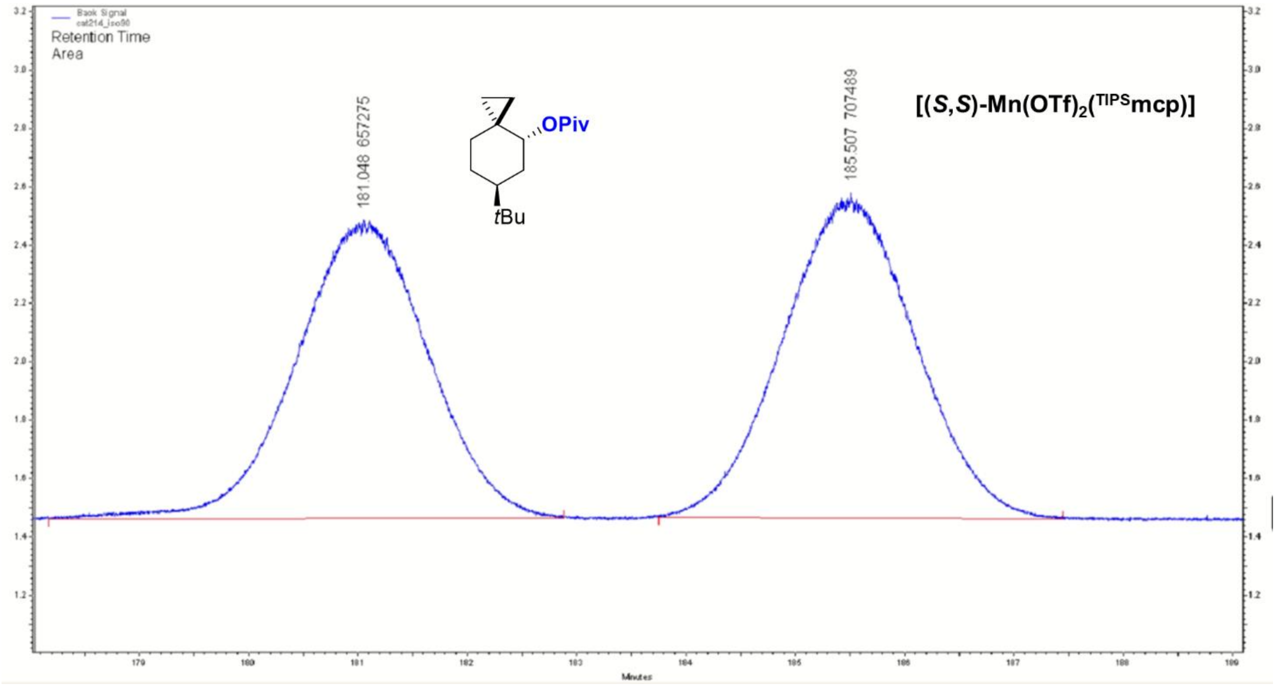

Figure S35: Chromatogram of chiral **P1a-OX3**.

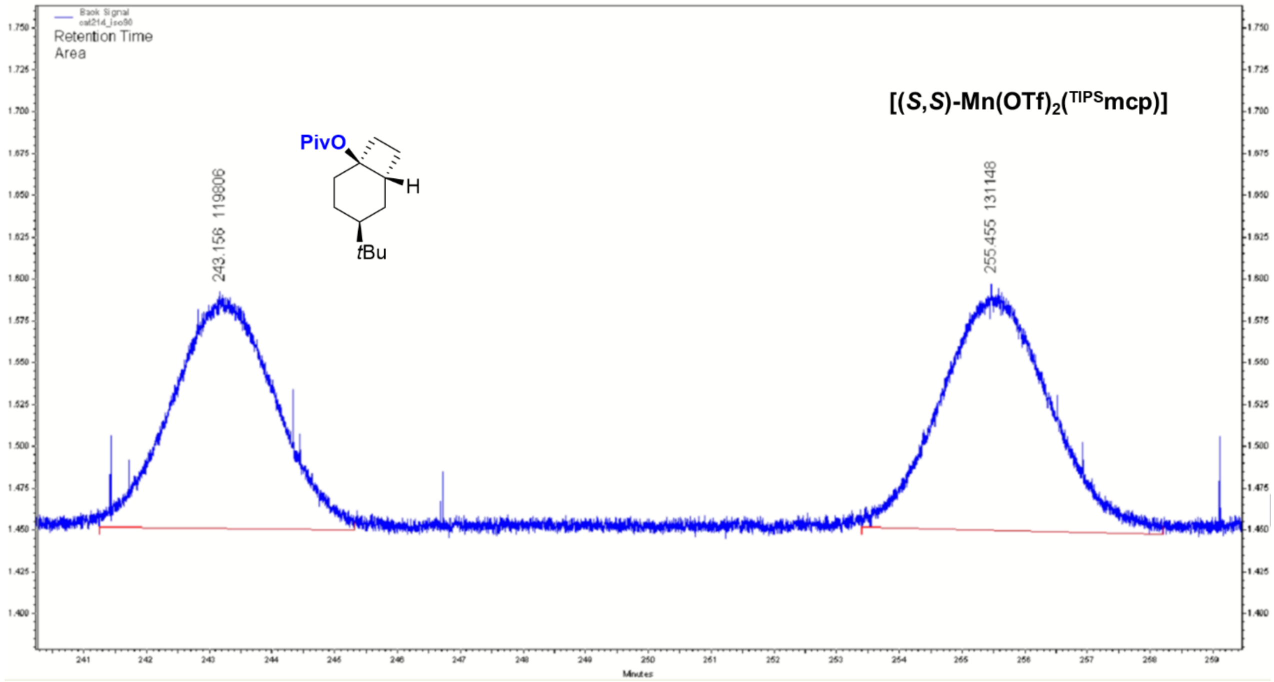

Figure S36: Chromatogram of chiral **P1b-OX3**.

- Oxidation of **S1** catalyzed by [(*S*,*S*)-Mn(OTf)$_2$(^{CF_3}mcp)] (entry 3, **Table S13**)

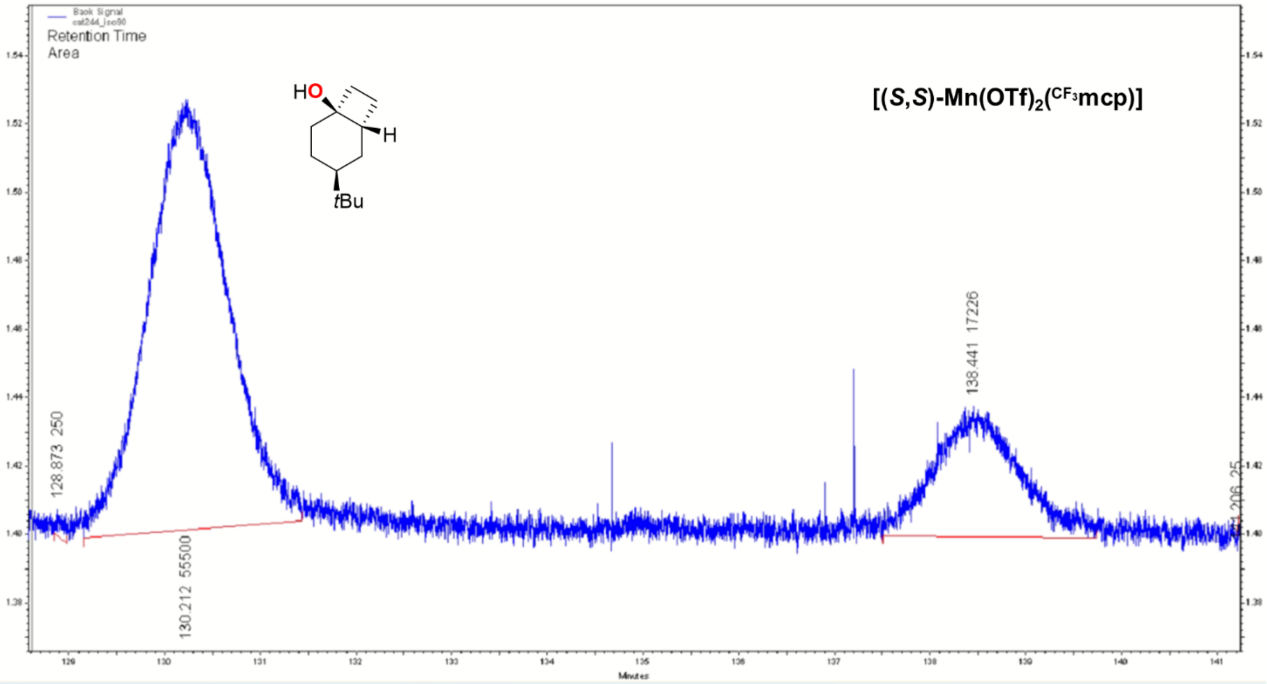

Figure S37: Chromatogram of chiral **P1b-OH**.

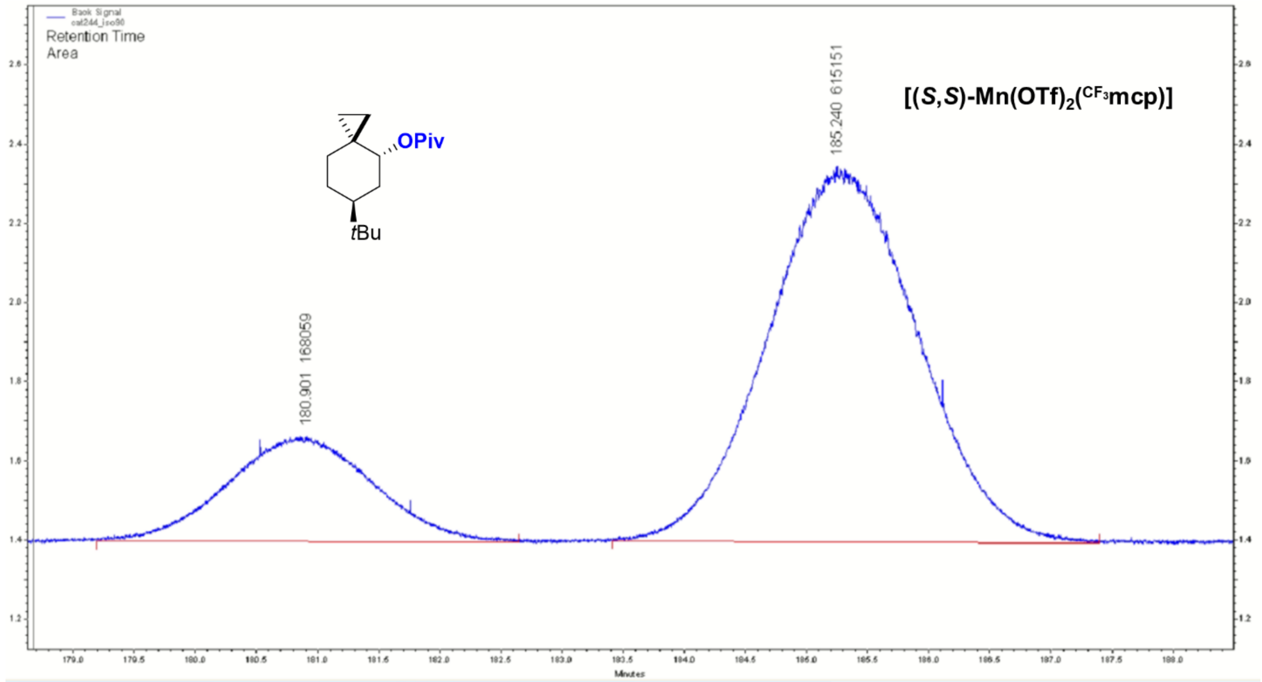

Figure S38: Chromatogram of chiral **P1a-OX$_3$**.

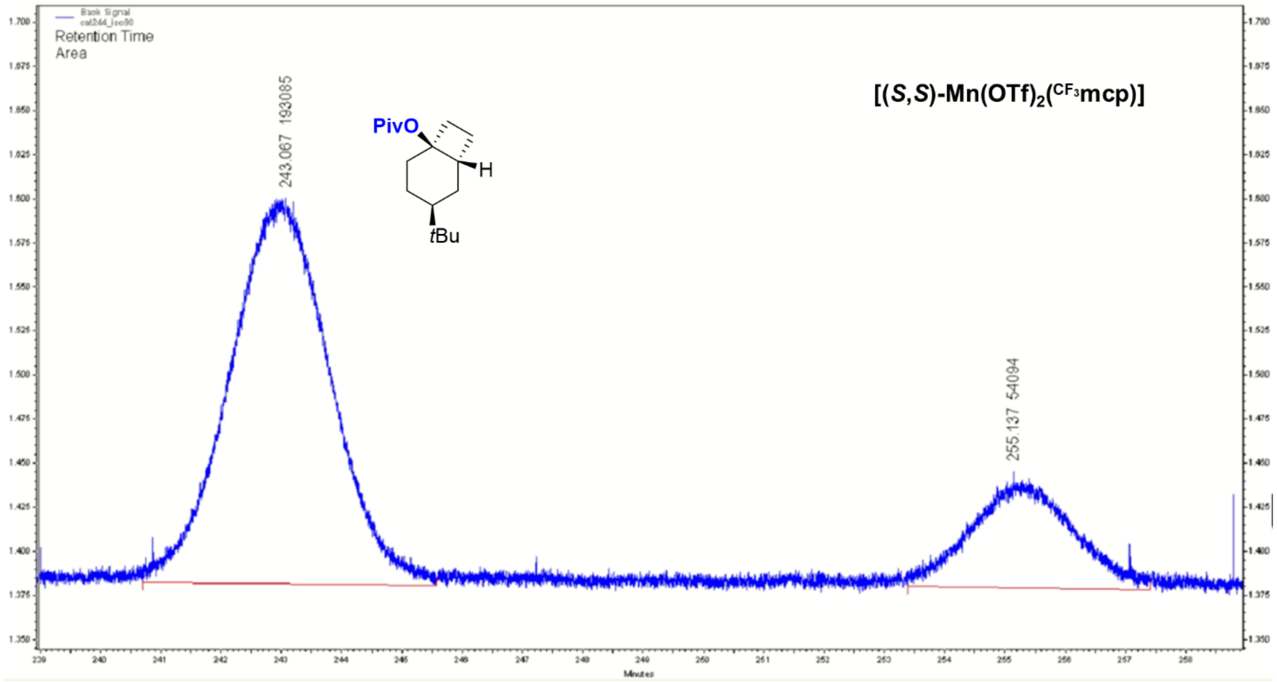

Figure S39: Chromatogram of chiral **P1b-OX3**.

- Oxidation of **S1** catalyzed by [(*R*,*R*)-Mn(OTf)$_2$(pdp)] (entry 4, **Table S13**)

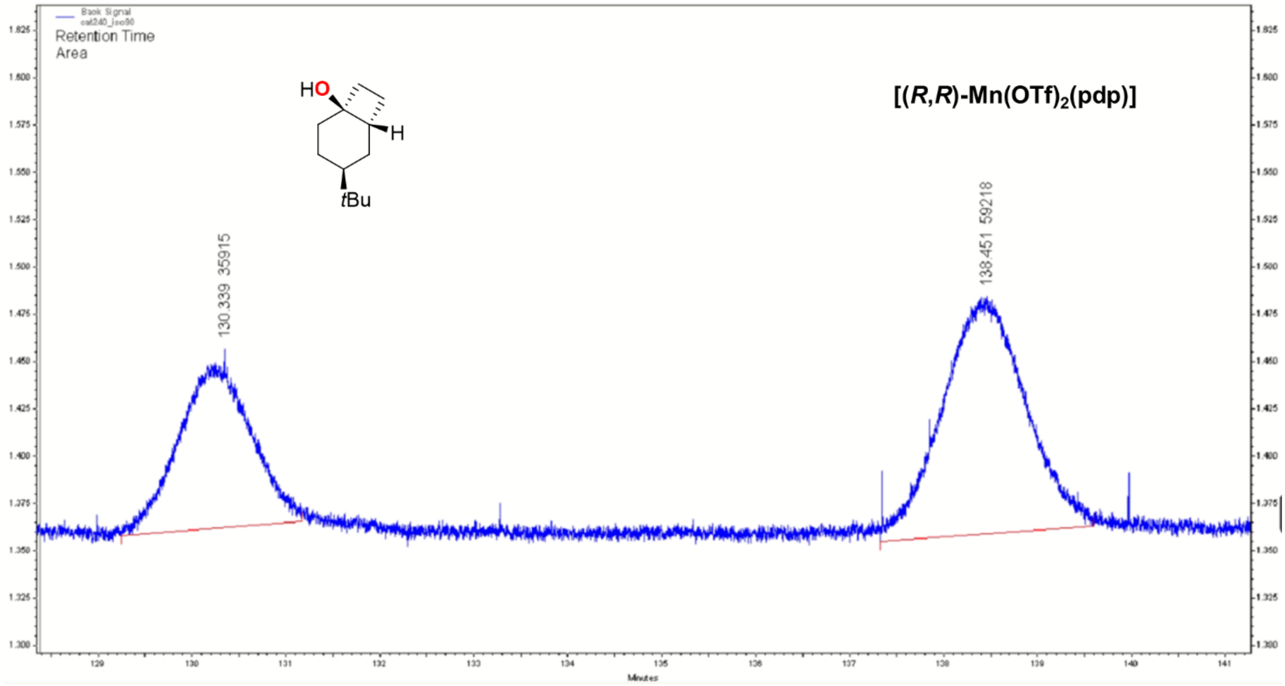

Figure S40: Chromatogram of chiral **P1b-OH**.

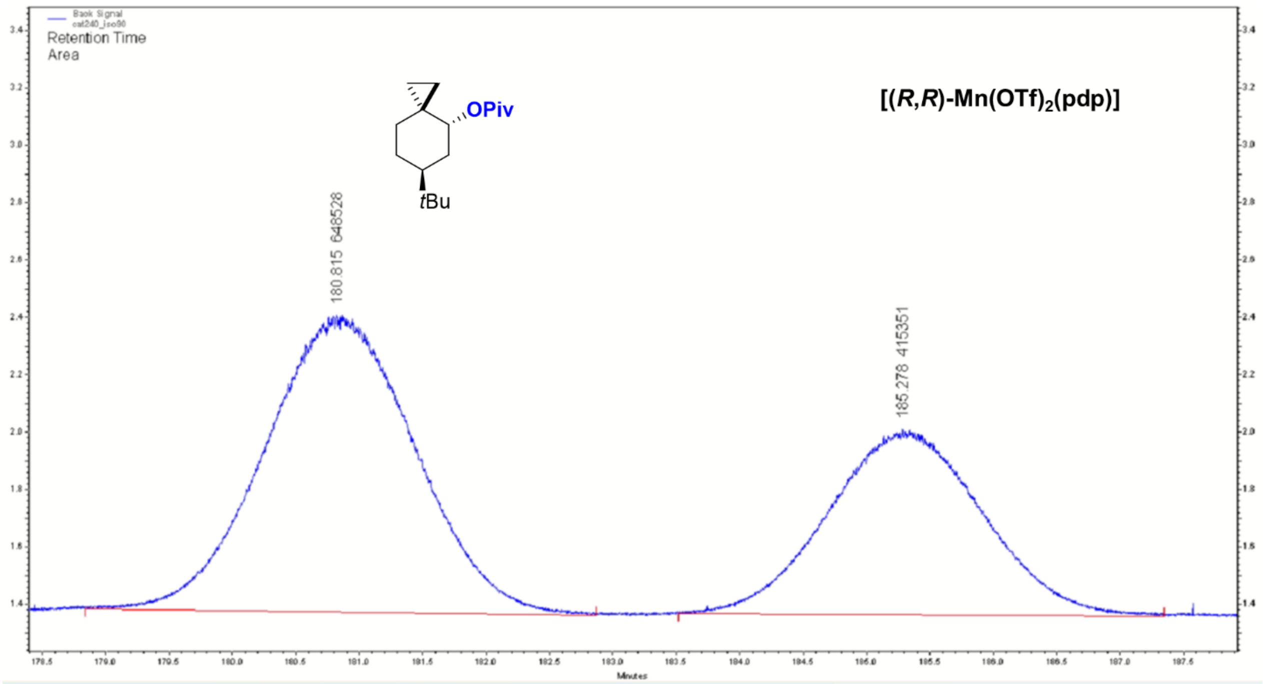

Figure S41: Chromatogram of chiral **P1a-OX3**.

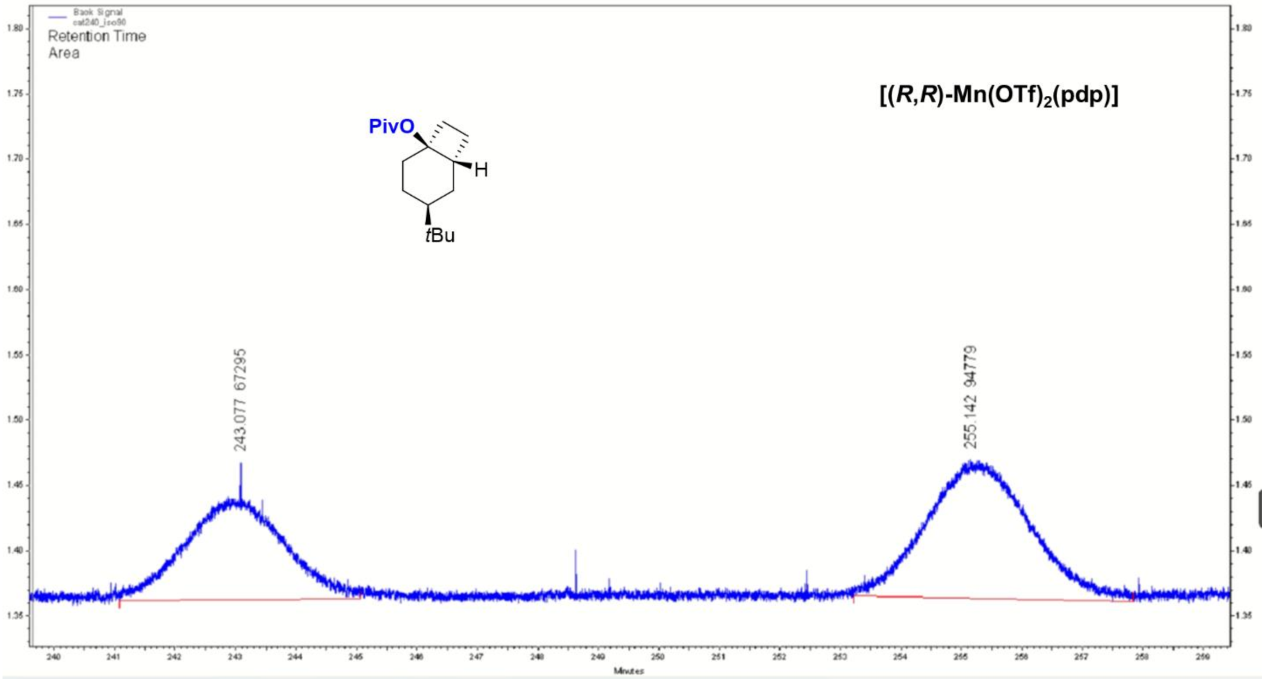

Figure S42: Chromatogram of chiral **P1b-OX3**.

- Oxidation of **S1** catalyzed by [(*S*,*S*)-Mn(OTf)$_2$(^{TIPS}pdp)] (entry 5, **Table S13**)

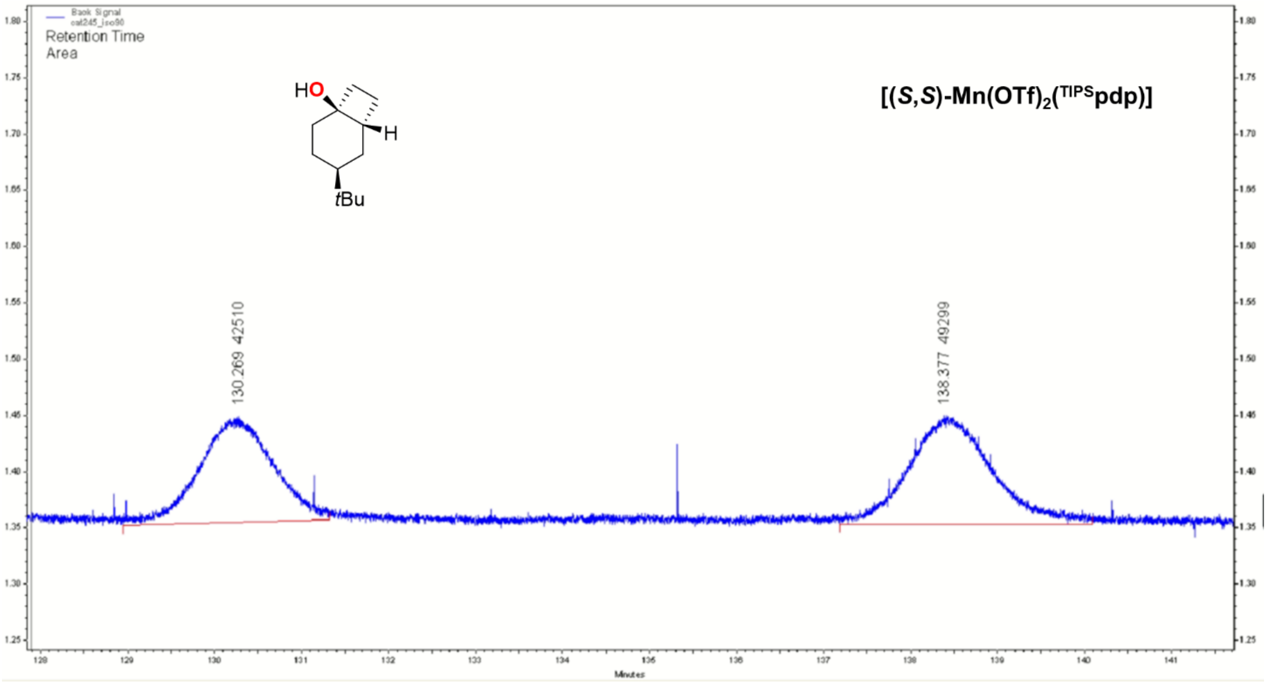

Figure S43: Chromatogram of chiral **P1b-OH**.

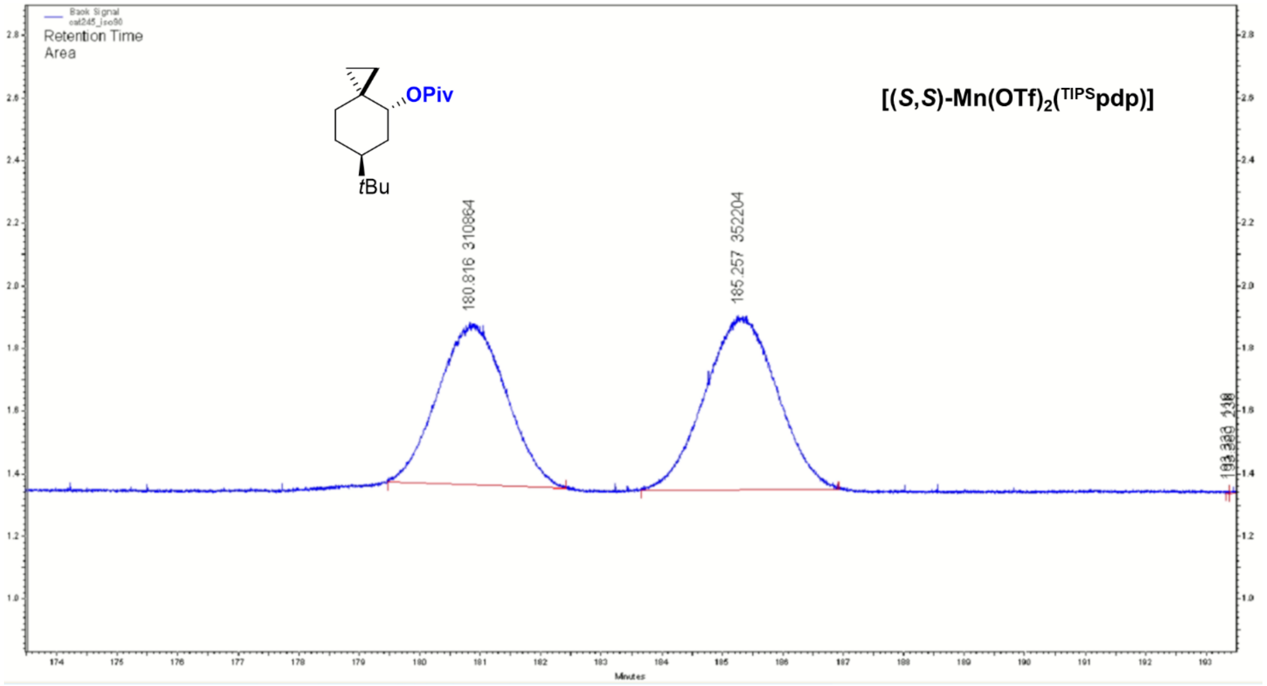

Figure S44: Chromatogram of chiral **P1a-OX$_3$**.

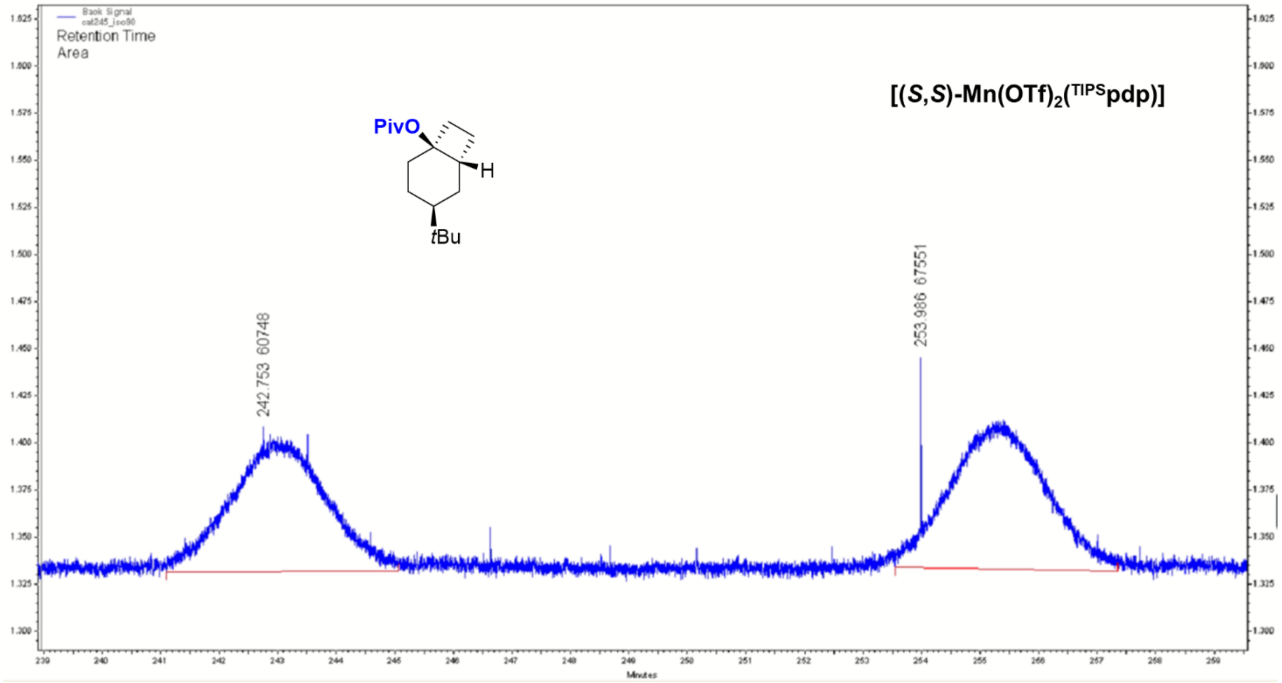

Figure S45: Chromatogram of chiral **P1b-OX3**.

- Oxidation of **S1** catalyzed by [(*S*,*S*)-Mn(OTf)$_2$(^{Bz}pdp)] (entry 6, **Table S13**)

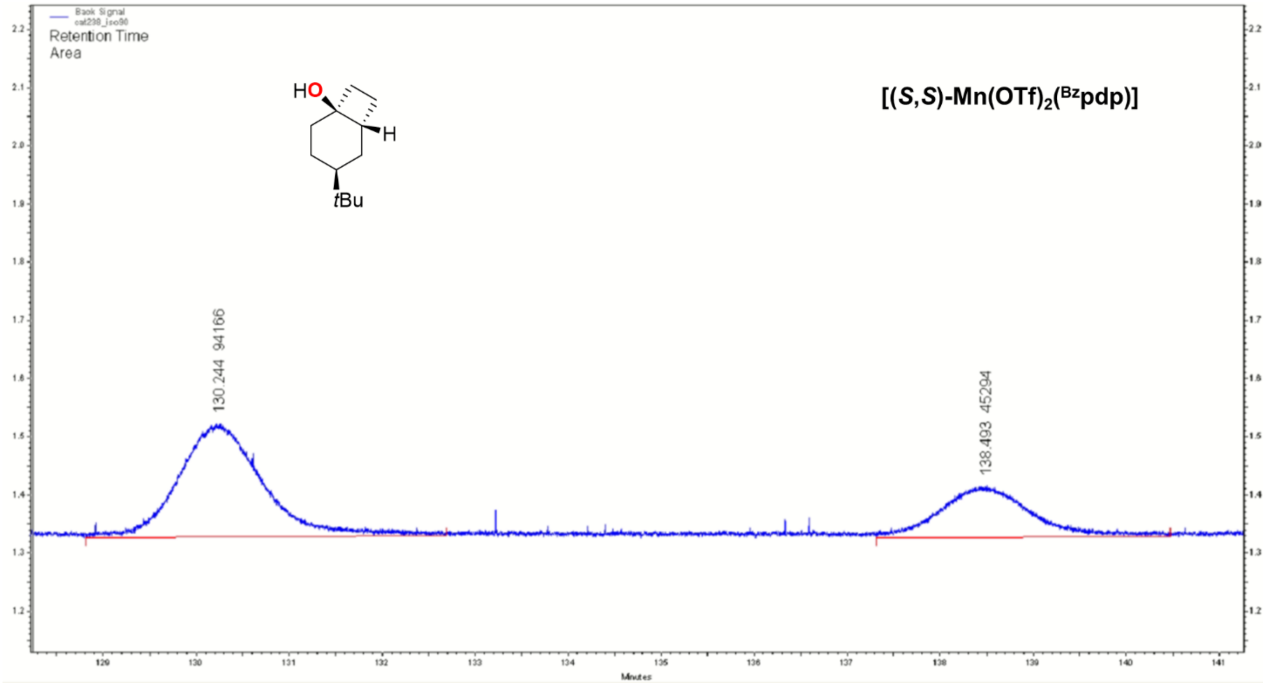

Figure S46: Chromatogram of chiral **P1b-OH**.

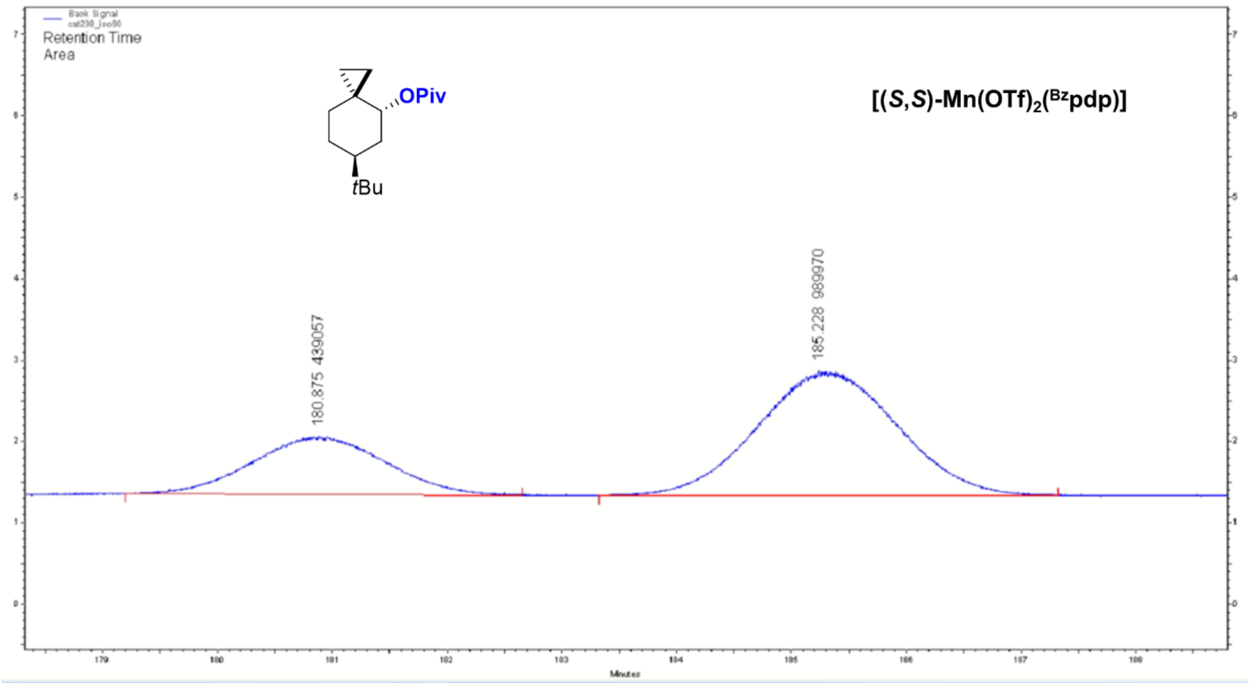

Figure S47: Chromatogram of chiral **P1a-OX3**.

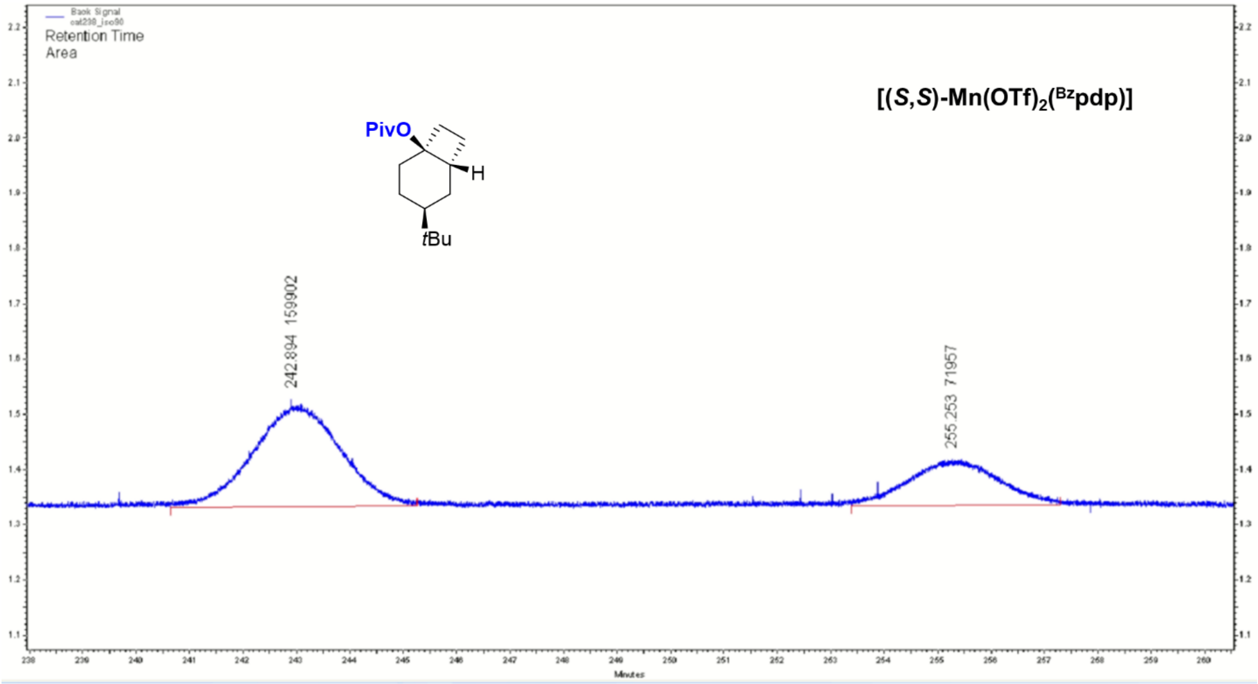

Figure S48: Chromatogram of chiral **P1b-OX3**.

- Oxidation of **S1** catalyzed by [(*S*,*S*)-Mn(OTf)$_2$(^{dMM}pdp)] (entry 7, **Table S13**)

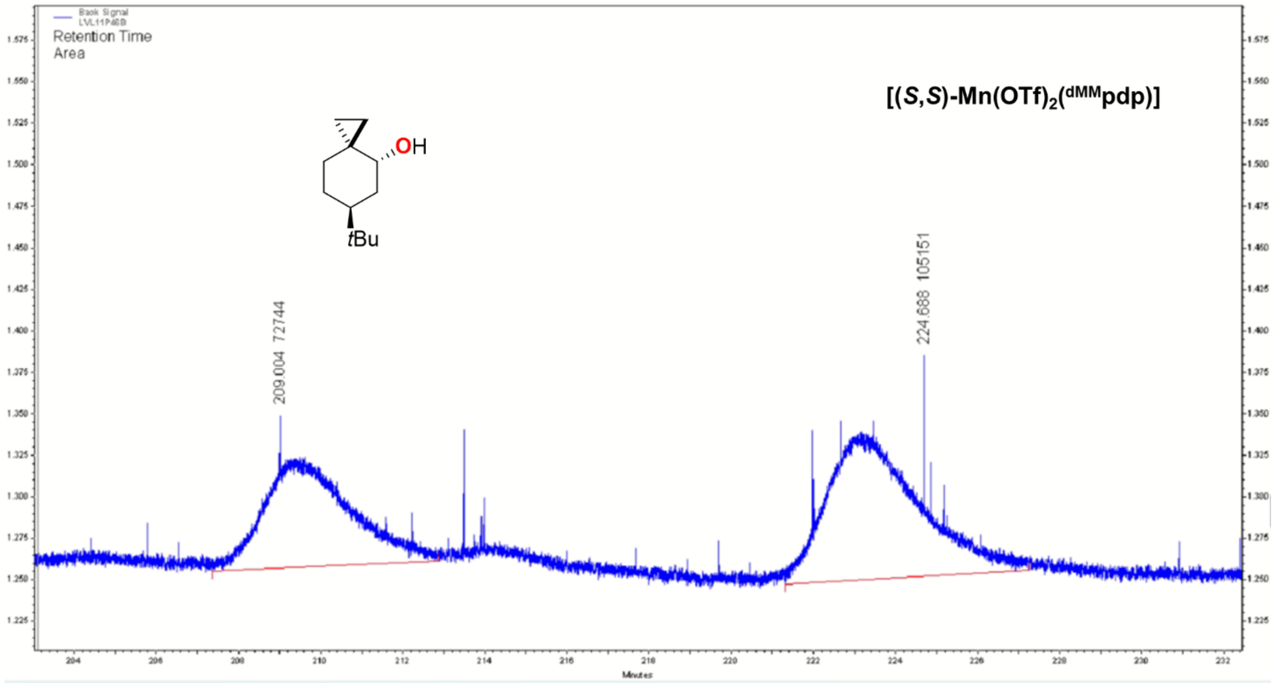

Figure S49: Chromatogram of chiral **P1a-OH**.

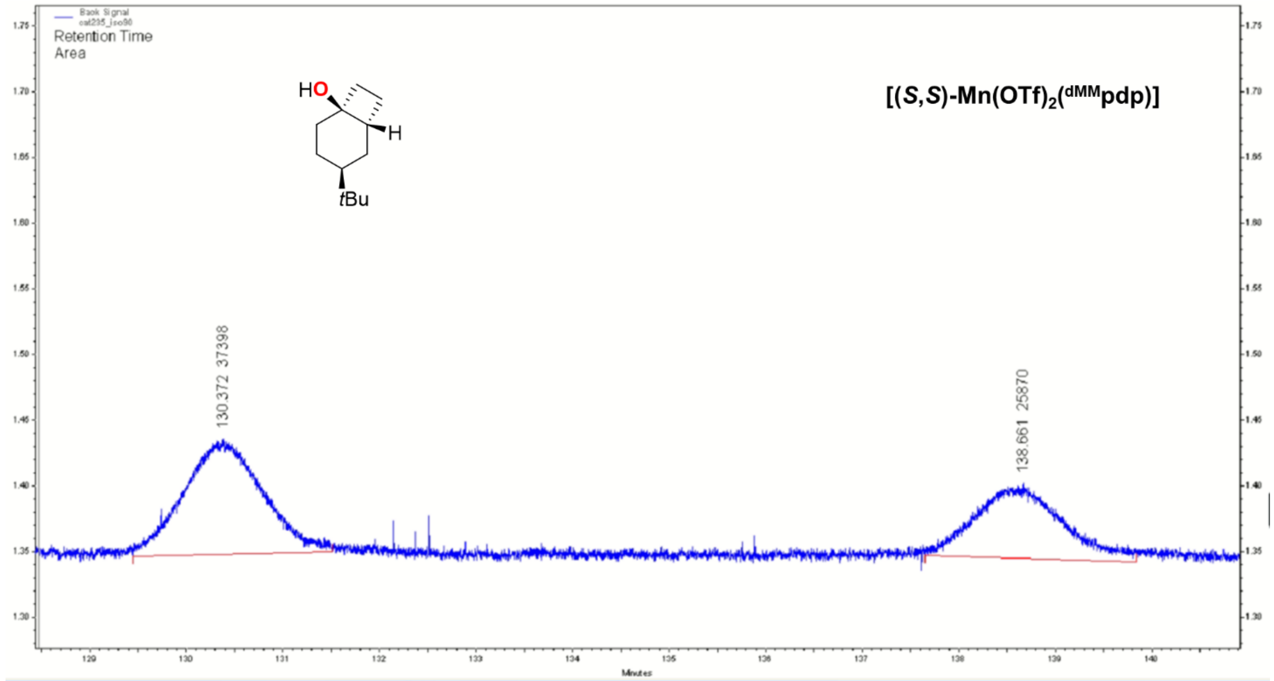

Figure S50: Chromatogram of chiral **P1b-OH**.

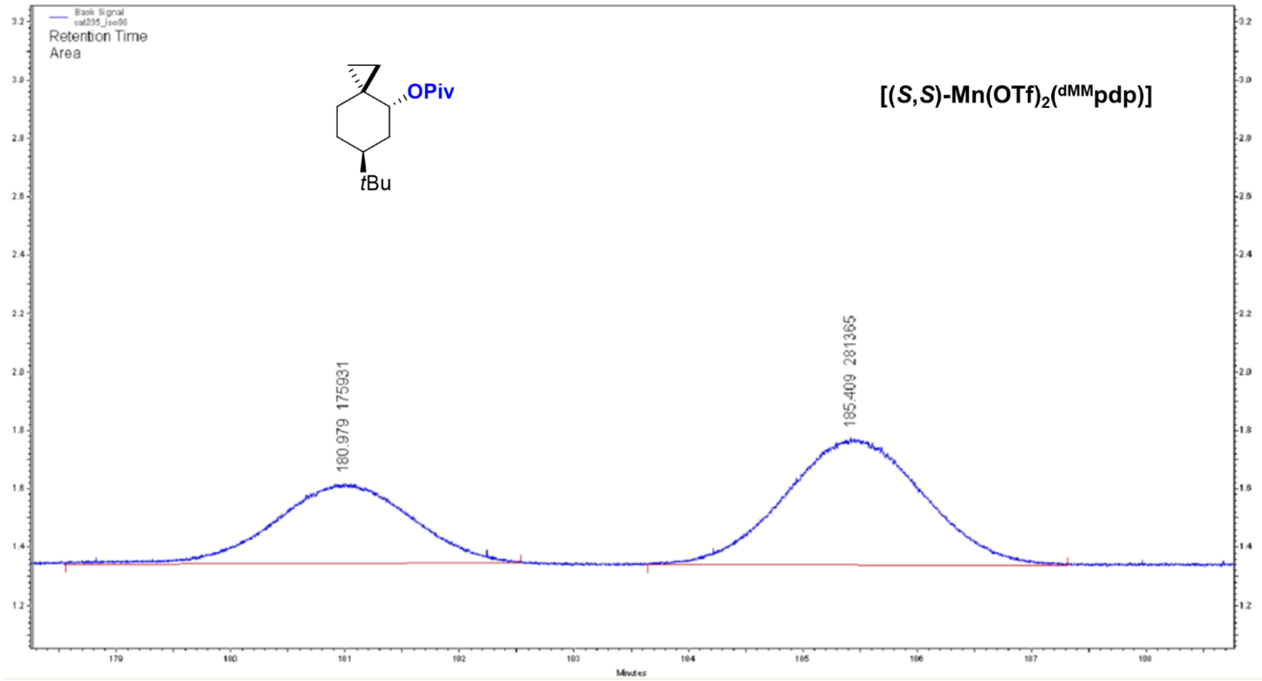

Figure S51: Chromatogram of chiral **P1a-OX3**.

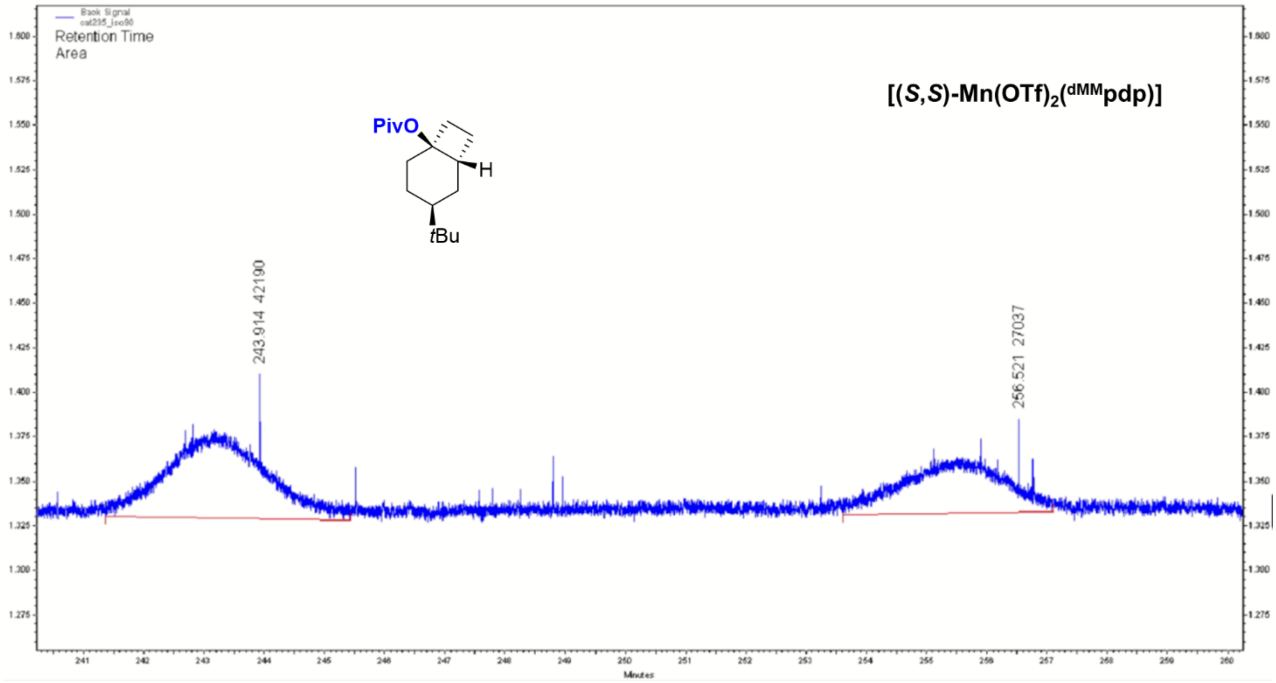

Figure S52: Chromatogram of chiral **P1b-OX3**.

- Oxidation of **S1** catalyzed by [(*S*,*S*)-Mn(OTf)$_2$(^{Me_2N}pdp)] (entry 8, **Table S13**)

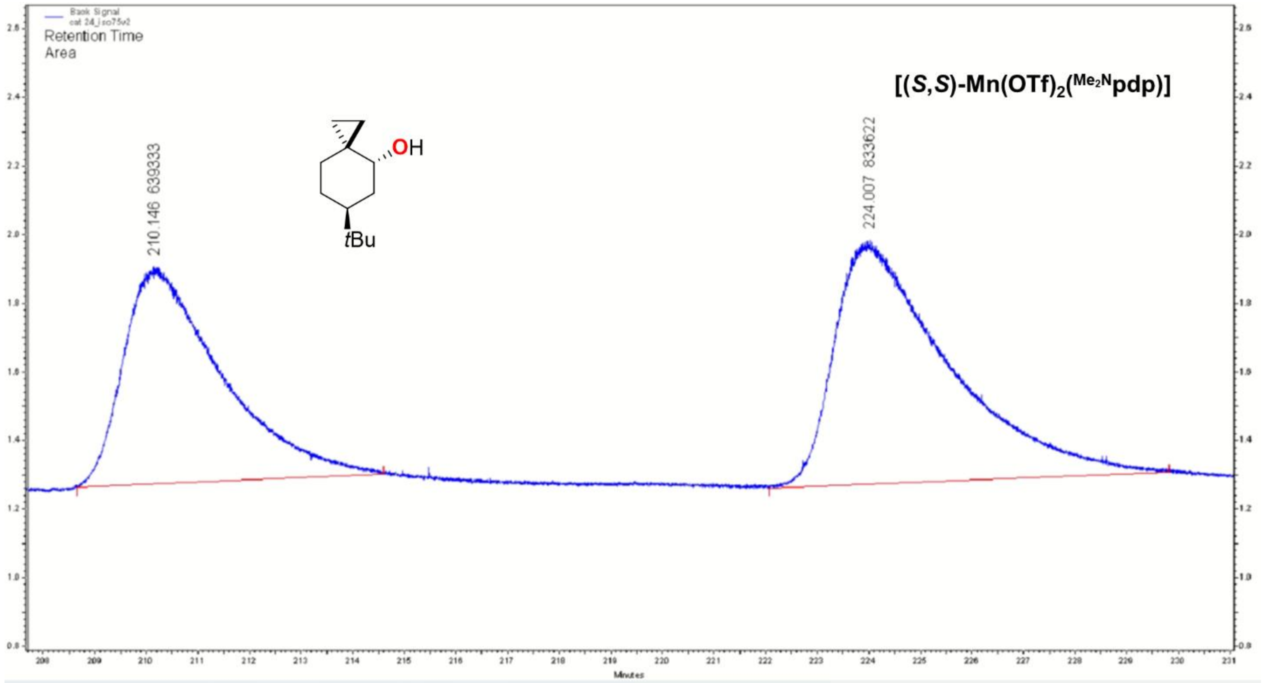

Figure S53: Chromatogram of chiral **P1a-OH**.

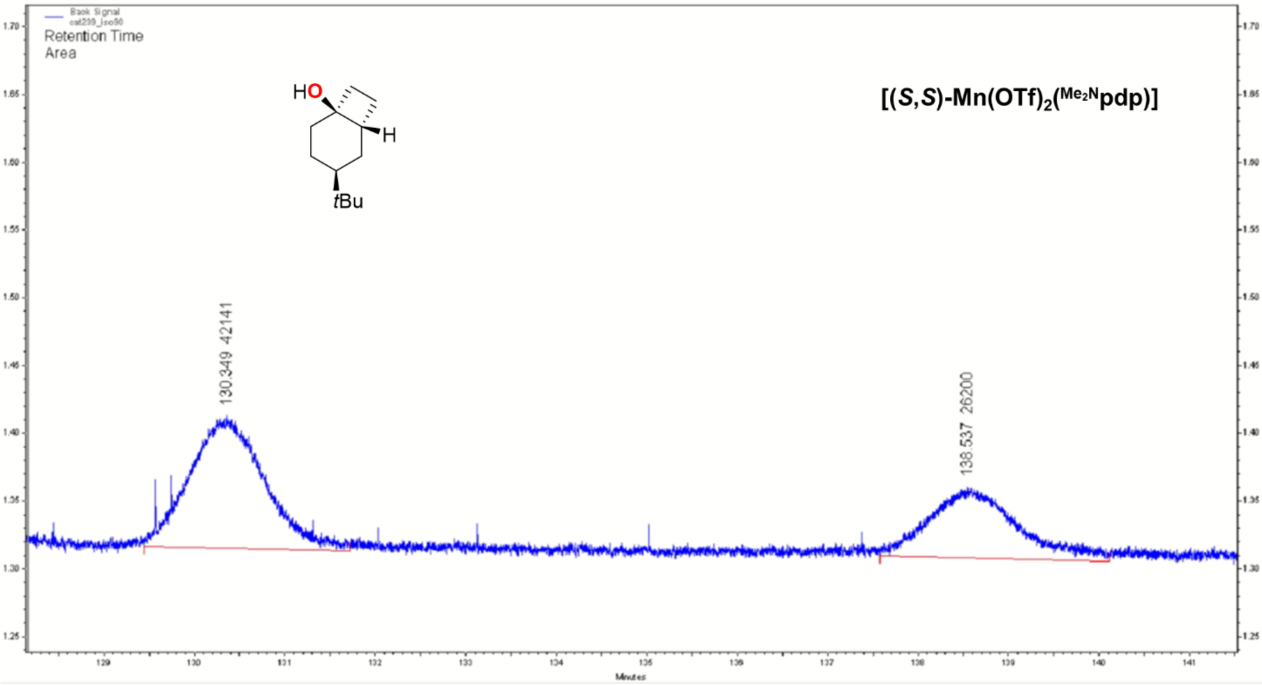

Figure S54: Chromatogram of chiral **P1b-OH**.

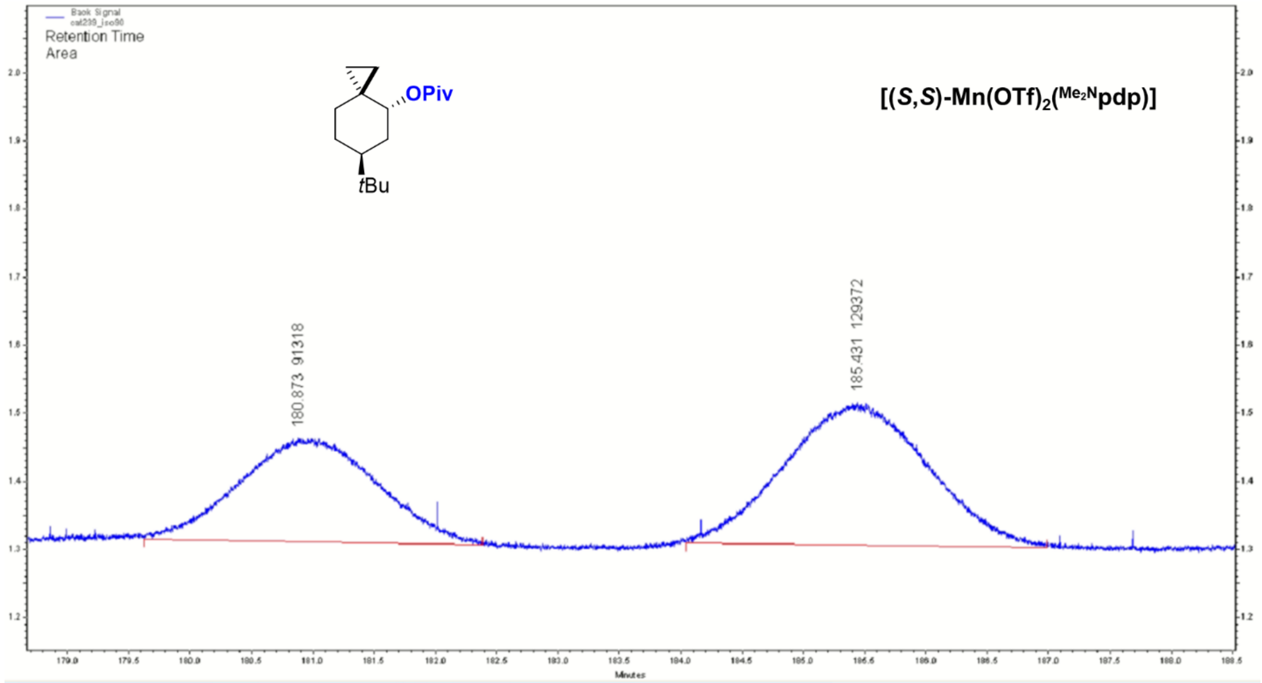

Figure S55: Chromatogram of chiral **P1a-OX3**.

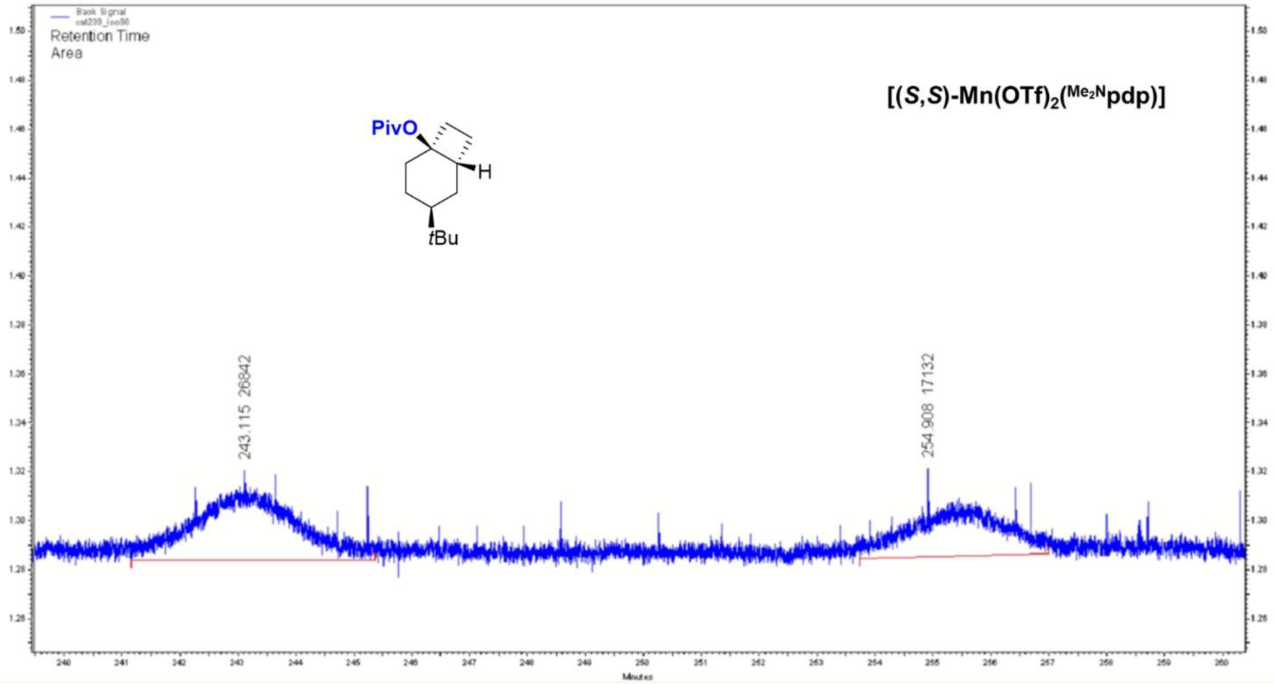

Figure S56: Chromatogram of chiral **P1b-OX3**.

Oxidation of **S1** catalyzed by [(*S*,*S*)-Mn(OTf)$_2$(^{Me_2N}pdp)] (entry 9, **Table S13**)

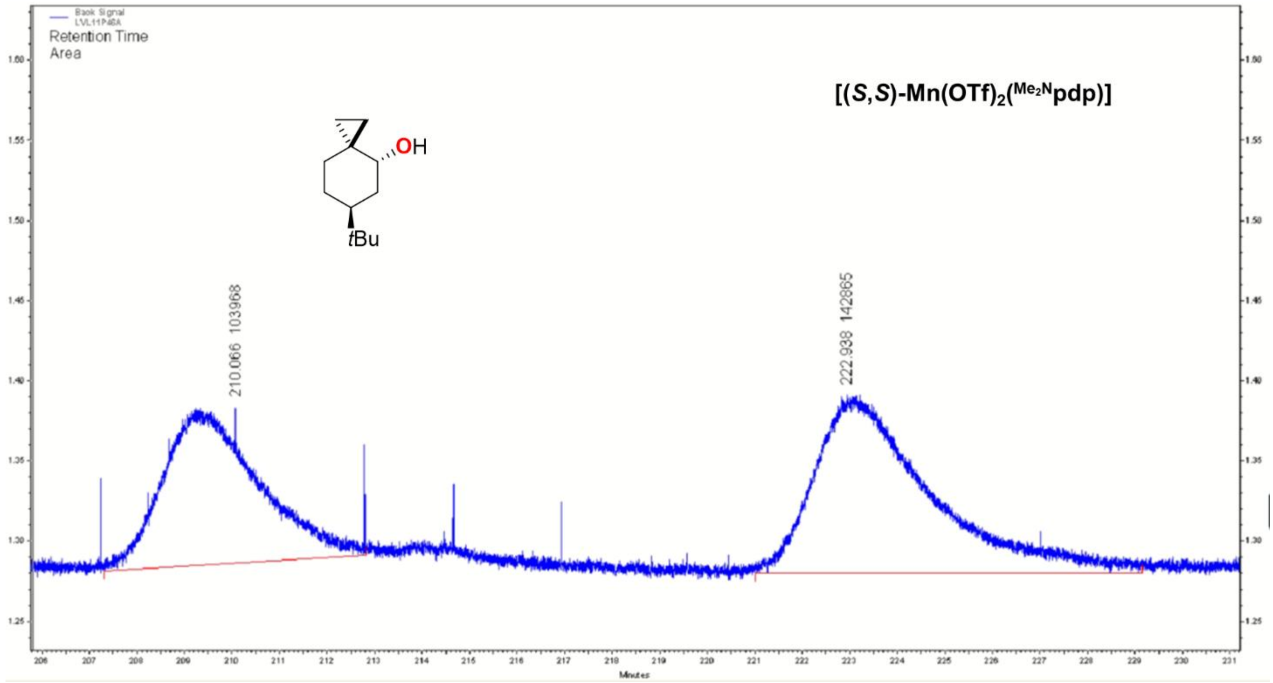

Figure S57: Chromatogram of chiral **P1a-OH**.

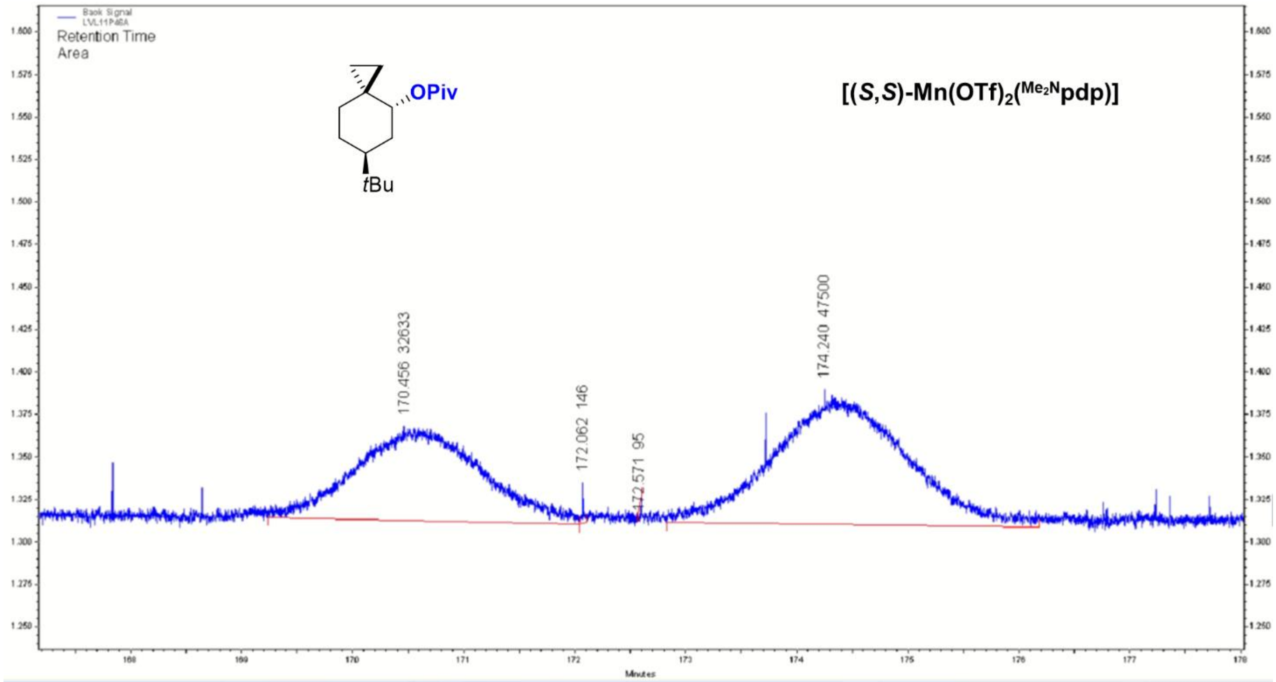

Figure S58: Chromatogram of chiral **P1a-OX$_3$**.

8. References

1. (a) Luo, W.; Lin, L.; Zhang, Y.; Liu, X.; Feng, X. *Org. Lett.* **2017**, *19*, 3374-3377. (b) Romanov-Michailidis, F.; Sedillo, K. F.; Neely, J. M.; Rovis, T. *J. Am. Chem. Soc.* **2015**, *137,* 8892-8895.

2. Friedrich, E. C.; Lewis, E. J. *J. Org. Chem.* **1990**, *55*, 2491-2494.

3. Jung, S. T.; Nickisch, R.; Reinsperger, T.; Luy, B.; Podlech, J. *J. Phys. Org. Chem.* **2021**, *34*, e4165.

4. Seyferth, D.; Turkel, R. L.; Eisert, M. A.; Todd, L. J. *J. Am. Chem. Soc.* **1969**, *91*, 5027-5033.

5 Murphy, A.; Dubois, G.; Stack, T. D. P. *J. Am. Chem. Soc.* **2003**, *125*, 5250-5251.

6. Milan, M.; Bietti, M.; Costas, M. *ACS Cent. Sci.* **2017**, *3*, 196-204.

7 Ottenbacher, R. V.; Bryliakov, K. P.; Talsi, E. P. *Adv. Synth. Catal.* **2011**, *353*, 885-889.

8 Wang, X.; Miao, C.; Wang, S.; Xia, C.; Sun, W. *ChemCatChem* **2013**, *5*, 2489-2494.

9. Cussó, O.; Garcia-Bosch, I.; Font, D.; Ribas, X.; Lloret-Fillol, J.; Costas, M. *Org. Lett.* **2013**, *15*, 6158-6161.

10. Lillie, T. S.; Ronald, R. C. *J. Org. Chem.* **1985**, *50*, 5084-5088.

11. Chen, M. S.; White, M. C. *Science* **2010**, *327*, 783-787.

12. Wubbolt, S.; Cheong, C. B.; Frost, J. R.; Christensen, K. E.; Donohoe, T. J. *Angew. Chem.* **2020**, *132*, 11435-11440.

13. Eisenbraun, E. J. *Org. Synth.* **1973**, *5*, 310-312.

14. (a) Hanack, v. M.; Schneider, H.-J.; *Liebigs Ann. Chem.* **1965**, *686*, 8-18. (b) Wiberg, K. B.; Hiatt, J. E.; Hseih, K. *J. Am. Chem. Soc.* **1970**, *92*, 544-553.

15. Lohr, T. R.; Li, Z.; Assary, R. S.; Curtiss, L. A.; Marks, T. J. *ACS catal.* **2015**, *5,* 3675-3679.
